# Spin-crossover and high-spin iron(ii) complexes as chemical shift ^19^F magnetic resonance thermometers†
†Electronic supplementary information (ESI) available: Experimental details, additional crystallographic, magnetic, and spectroscopic data, and crystallographic information files (CIFs) for **1a**·0.5CH_3_CN, **1b**, **2a**, **2b**, and **3a**. CCDC 1505471–1505473, 1505534, and 1505863. For ESI and crystallographic data in CIF or other electronic format see DOI: 10.1039/c6sc04287b



**DOI:** 10.1039/c6sc04287b

**Published:** 2017-01-06

**Authors:** Agnes E. Thorarinsdottir, Alexandra I. Gaudette, T. David Harris

**Affiliations:** a Department of Chemistry , Northwestern University , 2145 Sheridan Road , Evanston , IL 60208-3113 , USA . Email: dharris@northwestern.edu

## Abstract

The potential utility of paramagnetic transition metal complexes as chemical shift ^19^F magnetic resonance (MR) thermometers is demonstrated. Further, spin-crossover Fe^II^ complexes are shown to provide much higher temperature sensitivity than do the high-spin analogues, owing to the variation of spin state with temperature in the former complexes. This approach is illustrated through a series of Fe^II^ complexes supported by symmetrically and asymmetrically substituted 1,4,7-triazacyclononane ligand scaffolds bearing 3-fluoro-2-picolyl derivatives as pendent groups (L_*x*_). Variable-temperature magnetic susceptibility measurements, in conjunction with UV-vis and NMR data, show thermally-induced spin-crossover for [Fe(L_1_)]^2+^ in H_2_O, with *T*_1/2_ = 52(1) °C. Conversely, [Fe(L_2_)]^2+^ remains high-spin in the temperature range 4–61 °C. Variable-temperature ^19^F NMR spectra reveal the chemical shifts of the complexes to exhibit a linear temperature dependence, with the two peaks of the spin-crossover complex providing temperature sensitivities of +0.52(1) and +0.45(1) ppm per °C in H_2_O. These values represent more than two-fold higher sensitivity than that afforded by the high-spin analogue, and *ca.* 40-fold higher sensitivity than diamagnetic perfluorocarbon-based thermometers. Finally, these complexes exhibit excellent stability in a physiological environment, as evidenced by ^19^F NMR spectra collected in fetal bovine serum.

## Abstract 2

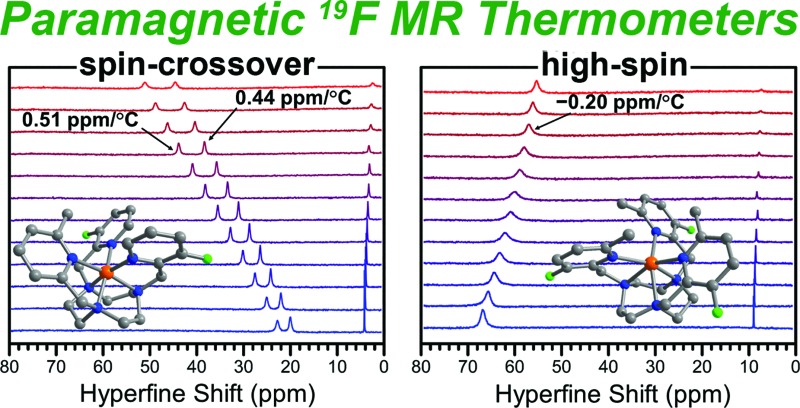
The potential utility of paramagnetic transition metal complexes as chemical shift ^19^F magnetic resonance (MR) thermometers is demonstrated.

## Introduction

The noninvasive measurement of temperature *in vivo* represents a growing area of research, largely due to its utility in medical applications such as low-temperature hyperthermia,^1,2^ high-temperature thermal ablation,^1,2^ and the treatment of heart arrhythmias.^3^ Here, thermometry may be used to discriminate normal from abnormal tissue, and also to ensure that thermal treatments are localized to prevent damage to healthy tissue.^1,2,4^ Magnetic resonance spectroscopy (MRS) and imaging (MRI) are particularly well-suited toward this end, owing to their use of non-ionizing radiation and ability to deeply penetrate tissue.^1,5^ Indeed, a number of temperature-sensitive MR parameters of water, including *T*_1_ and *T*_2_ relaxation times, proton resonance frequency (PRF), diffusion coefficient, and proton density, can be used to monitor tissue temperature.^1,4,6^ Currently, methods based on water PRF shift are the most widely used for imaging temperature in clinical studies due to their high-resolution and independence on tissue type.^7^ However, these techniques suffer from a low temperature sensitivity of *ca.* –0.01 ppm per °C, and their ability to accurately determine absolute temperature is limited.^1,7,8^

In order to overcome sensitivity limitations, paramagnetic lanthanide^9^ and transition metal complexes^10^ that function as MRS probes have been developed for thermometry. These complexes feature paramagnetically shifted proton resonances, thus minimizing the interference from background signal in biological tissue. In particular, proton resonances of Tm^3+^, Tb^3+^, Dy^3+^ and Yb^3+^ complexes have been shown to exhibit temperature sensitivities of up to 1.8 ppm per °C,^9*q*^ and have been employed for temperature mapping *in vitro* and *in vivo*.^9^ Additionally, transition metal MRS probes have been shown to exhibit similar sensitivity^10^ and may alleviate toxicity concerns associated with lanthanides.^11^

While paramagnetic MRS probes offer significant improvements in sensitivity over PRF thermometry, they are nevertheless limited to the inherent Curie temperature dependence of chemical shift in paramagnetic compounds.^12^ Alternatively, one can employ a strategy of tuning a physical parameter that itself depends on temperature and governs chemical shift. Since both contact (through-bond) and dipolar (through-space) hyperfine shift scale as *S*(*S* + 1), where *S* represents the electronic spin state, variation of *S* as a function of temperature can result in dramatic changes in chemical shift.^12^ As such, an ideal temperature-responsive chemical shift probe might feature a value of *S* that changes with temperature. Spin-crossover Fe^II^ complexes that undergo a thermally-induced electronic spin transition from a low-spin, *S* = 0 ground state to a high-spin, *S* = 2 excited state satisfy just such a criterion. Moreover, the ligand field in spin-crossover complexes can be chemically modulated to precisely tune the crossover temperature (*T*_1/2_), defined as the temperature at which the low-spin and high-spin states are equally populated,^13^ to near 37 °C. Indeed, the utility of spin-crossover in MR thermometry has been demonstrated through 
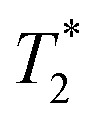
 modulation in Fe^II^-based nanoparticles^14^ and through paramagnetic chemical exchange saturation transfer (PARACEST) in molecular Fe^II^ complexes.^15^

While the vast majority of MRS thermometry probes exploit changes in the chemical shift of ^1^H NMR resonances, the employment of ^19^F MR offers several key advantages. First, the ^19^F nucleus features a 100% natural abundance, a nuclear spin of *I* = ^1^/_2_, and a gyromagnetic ratio and sensitivity close to that of ^1^H.^16^ Moreover, the near absence of endogenous fluorine signals in the body, the large spectral window of ^19^F resonances, and the remarkable sensitivity of ^19^F chemical shift to the local environment, give rise to NMR spectra with minimal peak overlap.^17^ Indeed, it has been demonstrated that ^19^F chemical shifts of transition metal porphyrin complexes are highly sensitive to their solution electronic structure, in particular to oxidation state and spin state.^18^ In addition, lanthanide-based ^19^F chemical shift probes for monitoring pH have been reported.^19^ However, despite the potential of *S* as a tunable parameter to increase the temperature sensitivity of ^19^F MR chemical shift, to our knowledge no paramagnetic ^19^F MR thermometers have been reported. In fact, diamagnetic perfluorocarbons represent the only examples of ^19^F MR thermometry, but the application of these compounds is limited by the small temperature dependence of their ^19^F chemical shifts that affords a maximum sensitivity of only 0.012 ppm per °C.^20^

Given the advantages of ^19^F over ^1^H MR, in conjunction with the temperature sensitivity of ^1^H MR chemical shifts of our previously reported spin-crossover Fe^II^ PARACEST probes^15^ and the high-spin Fe^II 1^H MR shift probes reported by Morrow and coworkers,^10^ we sought to develop fluorine-substituted spin-crossover and high-spin Fe^II^ complexes for chemical shift ^19^F MR thermometry. Herein, we report a series of complexes that feature new symmetrically and asymmetrically-substituted 1,4,7-triazacyclononane (tacn) derivatives with fluorinated 2-picolyl donors. The potential utility of spin-crossover and high-spin Fe^II^ complexes as chemical shift ^19^F MR thermometers is demonstrated through detailed analysis of their temperature-dependent spectroscopic and magnetic properties. Furthermore, these compounds exhibit excellent stability in a physiological environment, as revealed by variable-temperature ^19^F NMR spectra recorded in fetal bovine serum (FBS). To our knowledge, this work provides the first examples of paramagnetic chemical shift ^19^F MR thermometers.

## Results and discussion

### Syntheses and structures

With the goal to prepare air- and water-stable complexes, tacn-based ligands bearing three pendent pyridyl groups offer an ideal platform, as these hexadentate scaffolds have been shown to afford highly-stable Fe^II^ complexes.^10,21^ In addition, the ligand field can be readily tuned to obtain spin-crossover complexes within a physiologically relevant temperature range by chemical modulation of the electronic and steric properties of the pyridyl donors.^21e,22^ Toward this end, we sought to synthesize related ligands that support Fe^II^ complexes in selected spin states through controlled introduction of methyl groups into the 6-position of the pyridyl groups, which serves to weaken the ligand field by virtue of steric crowding at the Fe^II^ center. In addition, in order to enable utilization of these compounds in ^19^F MRS thermometry, we installed fluorine substituents onto the 3-positions of the pyridyl groups.

**Fig. 1:**
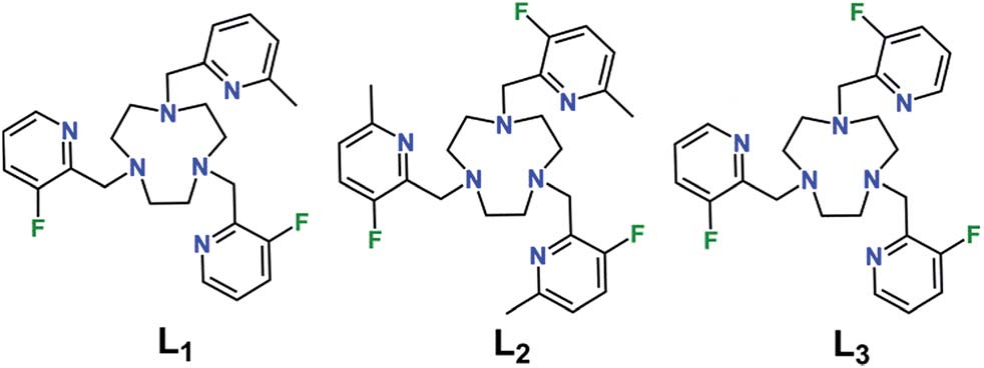
Molecular structures of ligands L_*x*_ (*x* = 1–3).

The preparation of ligands L_*x*_ (*x* = 1–3; see Fig. 1) was carried out through a five-step synthesis involving stepwise addition of 2-picolyl derivatives to the tacn backbone *via* reductive amination of the corresponding 2-pyridinecarboxaldehydes with tacn precursors (see Experimental section and Scheme S1†). Through judicious selection of the aldehyde reagent in each step, this synthetic route enabled the preparation of both symmetric and asymmetric tri-functionalized tacn-based ligands, appended with one or two types of 2-picolyl donors. Metalation of the ligands with Fe^II^ and Zn^II^ was effected through reaction of equimolar amounts of L_*x*_ and the corresponding divalent metal ion in CH_3_CN. Subsequent diffusion of Et_2_O into a concentrated CH_3_CN or CH_3_OH/CH_3_CN solution afforded crystalline [Fe(L_1_)][BF_4_]_2_·0.5CH_3_CN (**1a**·0.5CH_3_CN), [Zn(L_1_)][BF_4_]_2_ (**1b**), [Fe(L_2_)][BF_4_]_2_ (**2a**), [Zn(L_2_)][BF_4_]_2_ (**2b**), and [Fe(L_3_)][BF_4_]_2_ (**3a**).

Single-crystal X-ray diffraction analysis for **1a**·0.5CH_3_CN, **1b**, **2a**, **2b**, and **3a**, was carried out at 100 K (see Table S1†). Compound **1a**·0.5CH_3_CN crystallized in the triclinic space group *P*1, and features two [Fe(L_1_)]^2+^ cations in the asymmetric unit. Compound **1b** crystallized in the monoclinic space group *Pc*, with the asymmetric unit comprised of two [Zn(L_1_)]^2+^ cations. In contrast to the metal complexes of asymmetric L_1_, compounds **2a** and **2b** are isostructural and crystallized in the cubic space group *F*43*c*, with one third of the [M(L_2_)]^2+^ (M = Fe, Zn) cation in the asymmetric unit. In these two structures, the M^II^ metal center resides on a site of crystallographic three-fold symmetry. Finally, the asymmetric unit of the crystal structure of **3a**, which crystallized in the trigonal space group *P*3, features one-third of three unique [Fe(L_3_)]^2+^ cations, with the remainder of each complex related through a crystallographic three-fold axis (see Fig. S1†).

**Fig. 2:**
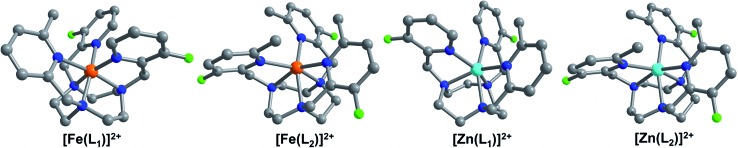
(Left–Right) Crystal structures of [Fe(L_*x*_)]^2+^ (*x* = 1, 2), as observed in **1a**·0.5CH_3_CN and **2a**, and [Zn(L_*x*_)]^2+^ (*x* = 1, 2), as observed in **1b** and **2b**. Turquoise, orange, green, blue and gray spheres represent Zn, Fe, F, N and C atoms, respectively; H atoms are omitted for clarity.

In the cationic complex of each compound, the M^II^ center resides in a distorted octahedral coordination environment, comprised of three facially bound tertiary amine nitrogen atoms from the tacn backbone and three picolyl nitrogen atoms (see Fig. 2). Examination of bond distances associated with the Fe^II^ cations reveals the spin state of these complexes in the solid-state at 100 K (see Table 1). The mean Fe–N bond distances for **1a**·0.5CH_3_CN and **3a** fall in the ranges 1.974(2)–2.088(2) and 1.969(3)–1.999(3) Å, respectively, indicative of low-spin Fe^II^.^15,22,23^ In **1a**·0.5CH_3_CN, the Fe–N_Me-pyr_ bond lengths of 2.085(2) and 2.090(2) Å are significantly longer than the Fe–N_F-pyr_ bond distances of 1.970(2)–1.978(2) Å, due to the steric effects imposed by the methyl substituent on one of the picolyl groups.^22^ In contrast, the average Fe–N_MeF-pyr_ and Fe–N_tacn_ bond distances for **2a** of 2.224(2) and 2.230(2) Å, respectively, are substantially longer and are characteristic of high-spin Fe^II^.^22,23a-c,24^ Finally, the mean Zn–N bond distances of 2.196(3) and 2.212(2) Å for **1b**, and **2b**, respectively, are consistent with reported distances for Zn^II^ ions in similar coordination environments.^25^

**Table 1:** Selected mean interatomic distances (Å) and angles (°) for **1a**·0.5CH_3_CN, **1b**, **2a**, **2b** and **3a** at 100 K

	**1a**·0.5CH_3_CN	**1b** ^*e*^	**2a**	**2b**	**3a**
M–N_tacn_	2.009(2)	2.206(3)	2.230(2)	2.217(2)	1.999(3)
M–N_Me-pyr_ ^*a*^	2.088(2)	2.225(4)	—	—	—
M–N_F-pyr_ ^*b*^	1.974(2)	2.167(4)	—	—	1.969(3)
M–N_MeF-pyr_ ^*c*^	—	—	2.224(2)	2.207(2)	—
N_tacn_–M–N_tacn_	85.07(6)	79.1(2)	78.40(8)	79.39(7)	86.3(2)
*cis* N_tacn_–M–N_Me-pyr_	90.38(6)	97.4(2)	—	—	—
*cis* N_tacn_–M–N_F-pyr_	89.08(6)	93.2(2)	—	—	90.0(1)
*cis* N_tacn_–M–N_MeF-pyr_	—	—	87.05(8)	87.34(7)	—
N_Me-pyr_–M–N_F-pyr_	96.79(7)	97.7(2)	—	—	—
N_F-pyr_–M–N_F-pyr_	94.59(6)	94.9(2)	—	—	94.07(9)
N_MeF-pyr_–M–N_MeF-pyr_	—	—	105.27(7)	104.21(6)	—
*trans* N_tacn_–M–N_Me-pyr_	166.76(7)	148.9(2)	—	—	—
*trans* N_tacn_–M–N_F-pyr_	168.02(7)	150.9(2)	—	—	169.7(1)
*trans* N_tacn_–M–N_MeF-pyr_	—	—	156.40(8)	157.85(7)	—
*Σ* ^*d*^	72.4(3)	159.7(5)	134.8(3)	127.7(2)	59.9(4)
M···F	5.102(2)	5.260(3)	5.277(2)	5.258(2)	5.094(2)

^*a*^N_Me-pyr_ corresponds to a N atom on a 6-methyl-2-picolyl group.

^*b*^N_F-pyr_ corresponds to a N atom on a 3-fluoro-2-picolyl group.

^*c*^N_MeF-pyr_ corresponds to a N atom on a 3-fluoro-6-methyl-2-picolyl group.

^*d*^Octahedral distortion parameter (*Σ*) = sum of the absolute deviations from 90° of the 12 *cis* angles in the MN_6_ coordination sphere.

^*e*^Data obtained from Zn1 due to severe crystallographic disorder associated with Zn2.

The presence of fluoro and methyl substituents on the 2-picolyl pendent groups of ligands L_1–3_ leads to a distortion from octahedral coordination at the metal centers. This deviation from perfect octahedral geometry can be quantified through the octahedral distortion parameter *Σ*, defined as the sum of the absolute deviations of the 12 *cis*-oriented N–M–N angles from 90°.^26^ Analysis of the Fe^II^ centers in **1a**·0.5CH_3_CN, **2a**, and **3a** gives values of *Σ* = 72.4(3), 134.8(3), and 59.9(4)°, respectively. The much larger value for **2a** than for **1a**·0.5CH_3_CN and **3a** reflects the significant steric crowding in **2a** and further corroborates the high-spin and low-spin assignments of these complexes.^27^ The larger distortion of the [Fe(L_1_)]^2+^ cation in **1a**·0.5CH_3_CN relative to [Fe(L_3_)]^2+^ in **3a** is attributed to presence of one *vs.* zero picolyl methyl substituents, respectively. The coordination environment of the Fe^II^ complex in **2a** and its isostructural Zn^II^ analogue in **2b** are similar, where **2b** is slightly less distorted than **2a**, evident from a smaller *Σ* value of 127.7(2)°. In contrast, the difference between the structures of **1a**·0.5CH_3_CN and **1b** is substantial. Upon moving from Fe to Zn, the mean N_tacn_–M–N_tacn_ angle decreases by 7.1%, from 85.07(6) to 79.1(2)°, and the mean *trans* N_tacn_–M–N_pyr_ angles decrease by 10.7 (N_Me-pyr_), and 10.2% (N_F-pyr_), respectively. Finally, a more than two-fold increase in *Σ* is observed for **1b** relative to **1a**·0.5CH_3_CN. These differences reflect a much greater degree of distortion at the Zn^II^ center in **1b** than at the Fe^II^ center in **1a**·0.5CH_3_CN, which likely stems from increased coordination flexibility at the d^10^ Zn^II^ ion due to lack of ligand field stabilization, and the larger six-coordinate ionic radius of Zn^II^ (0.88 Å) compared to low-spin Fe^II^ (0.75 Å).^27*a*^

Compounds **1a**·0.5CH_3_CN, **1b**, **2a**, **2b**, and **3a** feature intramolecular M···F distances in the range 5.094(2)–5.277(2) Å. The shortest M···F distances are observed between the 3-fluoro-2-picolyl pendent groups and the Fe^II^ centers in compounds **1a**·0.5CH_3_CN and **3a**, with slightly longer M···F distances of 5.26–5.28 Å in compounds **1b**, **2a**, and **2b**. The longer Zn···F distance in **1b**, compared to the corresponding Fe···F distance in **1a**·0.5CH_3_CN, can be attributed to the longer Zn–N bond distances relative to Fe. In the case of compounds **2a** and **2b**, the presence of bulky 3-fluoro-6-methyl-2-picolyl groups increase the M···F distances relative to **1a**·0.5CH_3_CN and **3a**. Importantly, the M···F distances of **1a** and **2a** are within the optimal range of 4.5–7.5 Å to balance the benefits of paramagnetic hyperfine shift with the decrease in sensitivity due to spectral broadening,^19d,e^ which demonstrates the potential of these complexes as candidates for ^19^F chemical shift MR probes.

### UV-vis spectroscopy

**Fig. 3:**
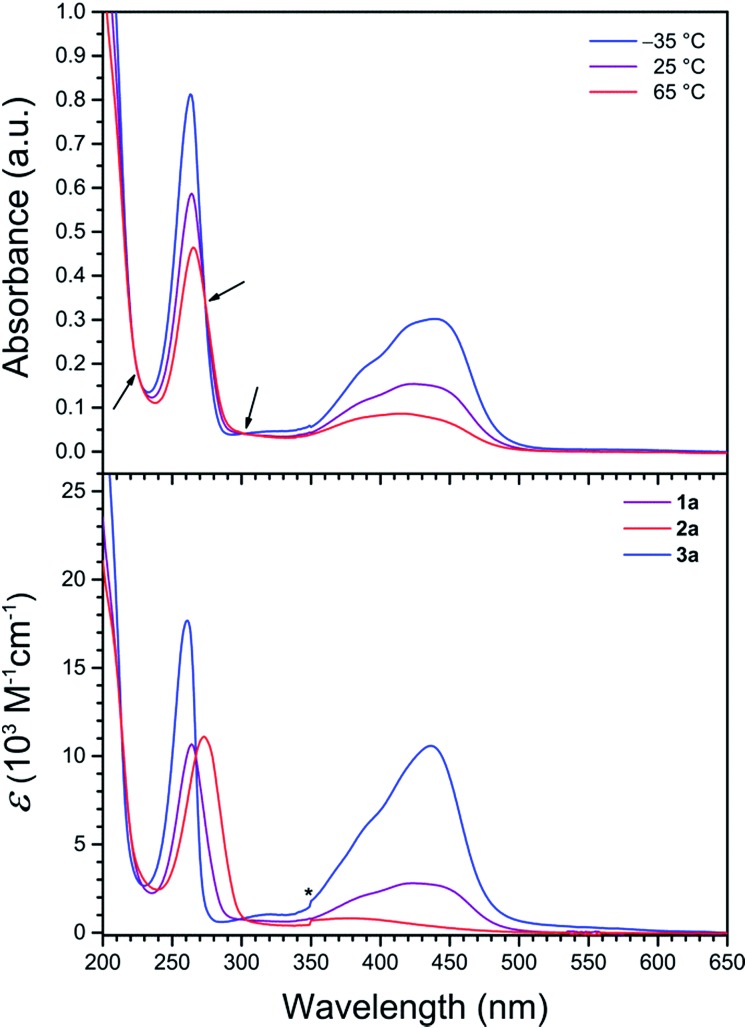
(Upper) UV-vis spectra of **1a** in CH_3_CN at selected temperatures. Arrows denote isosbestic points. (Lower) UV-vis spectra in CH_3_CN at 25 °C. The asterisk denotes an instrumental artifact.

To probe the solution electronic structures of the cationic complexes in **1a**, **1b**, **2a**, **2b**, and **3a**, UV-vis absorption spectra were collected for crystalline samples in CH_3_CN solution. The spectrum of **1a** obtained at 25 °C exhibits an intense band at 264 nm (*ε* = 10 700 M^–1^ cm^–1^), in addition to a weaker broad band at 424 nm (*ε* = 2800 M^–1^ cm^–1^) with a high-energy shoulder (see Fig. 3 and S2†). Based on literature precedent of Fe^II^ complexes in similar ligand environments, we assign these absorption bands as ligand-centered π–π* and metal–ligand charge transfer (MLCT) transitions, respectively.^22,28^ The UV-vis spectrum of **2a** at 25 °C is dominated by the intense π–π* band (*λ*_max_ = 273 nm, *ε*_max_ = 11 100 M^–1^ cm^–1^), and an additional broad feature of low intensity between 320 and 460 nm (*λ*_max_ = 375 nm) corresponds to a MLCT transition (see Fig. 3, lower, and S3†). The weak intensity and the small temperature dependence between –35 and 65 °C for the latter band (*ε*_max_ = 1000 *vs.* 700 M^–1^ cm^–1^, respectively) are characteristic of high-spin Fe^II^.^28c,29^ Compound **3a** is also relatively insensitive to temperature changes and at 25 °C displays a similar ligand-centered π–π* transition at 261 nm, but with a more intense MLCT band at 436 nm (*ε*_max_ = 10 600 M^–1^ cm^–1^), and as such is indicative of low-spin Fe^II^ (see Fig. 3, lower, and S4†).^22,30^ The variable-temperature UV-vis spectra of the Zn^II^ compounds **1b** and **2b** in CH_3_CN each exhibits a single intense band with *λ*_max_ = 268 and 278 nm, respectively (see Fig. S5 and S6†), consistent with ligand-centered π–π* transitions.^31^

The absorption spectra of **1a** demonstrate remarkable temperature dependence between –35 and 65 °C (see Fig. 3, upper). While the position of the π–π* band is relatively invariant to temperature, *ε*_max_ decreases significantly from 14 800 to 8400 M^–1^ cm^–1^ upon warming, as has been observed for related pyridyl complexes.^32^ At –35 °C, the MLCT band exhibits a *λ*_max_ value of 439 nm (*ε*_max_ = 5500 M^–1^ cm^–1^) with a shoulder at *ca.* 385 nm. Upon warming, the MLCT bands broaden and decrease in intensity, resulting in a single peak with *λ*_max_ = 385 nm (*ε*_max_ = 1600 M^–1^ cm^–1^) at 65 °C that corresponds to *ca.* 3.5-fold reduction in intensity from the –35 °C spectrum. This temperature dependence of the spectra is indicative of a thermally-induced spin state transition.^22,33^ Indeed, approximating a metal complex of *O*_h_ symmetry, the intensity of the MLCT band is directly correlated to the number of electrons in t_2g_ orbitals.^32c,d^ As such, moving from low-spin Fe^II^ (t62g) to high-spin Fe^II^ (t42ge2g) with increasing temperature results in a weaker absorption. Moreover, the presence of three isosbestic points at 222, 273, and 302 nm suggests an equilibrium between two spin states for the Fe^II^ centers in **1a**.

The temperature-dependent spin state of Fe^II^ in **1a** in CH_3_CN can be further examined by comparing the UV-vis spectra of **1a** with the corresponding spectra of the high-spin compound **2a** and the low-spin compound **3a** (see Fig. 3, lower). At lower temperature, the spectrum of **1a** strongly resembles that of **3a** (see Fig. S7†), whereas at higher temperature the broad spectrum resembles that of **2a** (see Fig. S8†). These temperature-dependent spectral changes demonstrate the thermally-induced spin-crossover of **1a** in CH_3_CN solution from primary population of a low-spin state at –35 °C to a high-spin state at 65 °C.

With an eye toward employing these complexes in MR thermometry, UV-vis spectra were collected for aqueous solutions of compounds **1a**, **1b**, **2a**, **2b**, and **3a** at ambient temperature. All compounds show similar characteristics in H_2_O as in CH_3_CN, giving comparable values of *λ*_max_ and *ε*_max_ (see Fig. S9–S13†). Nevertheless, the spectrum of **1a** in H_2_O reveals some key differences from the spectrum obtained in CH_3_CN at 25 °C. The absorption maximum of the MLCT band is shifted to a longer wavelength in H_2_O (*λ*_max_ = 436 nm), and the intensity of this band compared to the intensity of the analogous band for **3a** in the same solvent is considerably greater in H_2_O than in CH_3_CN (H_2_O: *ε*_max,_ **_3a_**/*ε*_max,_ **_1a_** = 1.5; CH_3_CN: *ε*_max,_ **_3a_**/*ε*_max,_ **_1a_** = 3.8). These observations indicate that moving from CH_3_CN to H_2_O serves to stabilize the low-spin state of [Fe(L_1_)]^2+^, leading to a higher *T*_1/2_. Similar trends have been reported for other spin-crossover Fe^II^ complexes and stem from the donor strength of the two solvents.^34^ Importantly, **1a** exhibits remarkable water and air stability, as the absorption spectra of this compound in deoxygenated water and after four weeks in oxygenated water are identical (see Fig. S9†).

### Magnetic properties

**Fig. 4:**
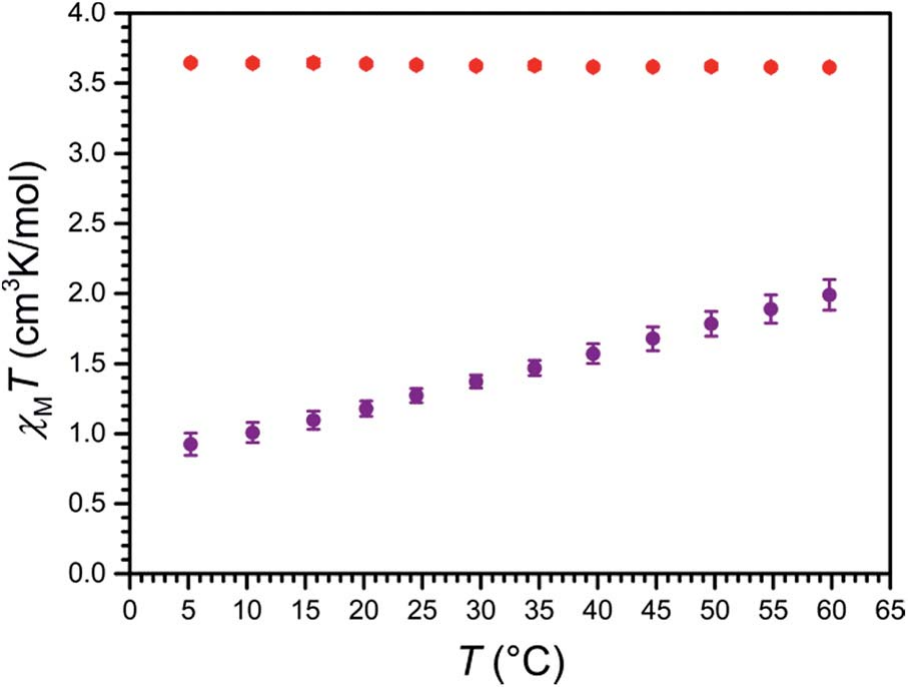
Variable-temperature magnetic susceptibility data for aqueous solutions of **1a** (purple) and **2a** (red), obtained in a 9.4 T NMR spectrometer using the Evans method. Error bars represent standard deviations of the measurements.

To probe the magnetic properties of compounds **1a** and **2a**, variable-temperature magnetic susceptibility data were collected in the temperature range 5–60 °C for aqueous solutions in a 9.4 T NMR spectrometer using the Evans method (see Fig. 4).^35^ For **2a**, *χ*_M_ *T* is constant over this temperature range, with an average value of *χ*_M_ *T* = 3.63 cm^3^ K mol^–1^ that corresponds to a high-spin, *S* = 2 Fe^II^ ion with *g* = 2.20. In stark contrast, for **1a**, *χ*_M_ *T* increases nearly linearly with increasing temperature, from a minimum value of 0.93 cm^3^ K mol^–1^ at 5 °C to a maximum value of 1.99 cm^3^ K mol^–1^ at 60 °C, indicative of thermally-induced spin-crossover. Note that the high-spin excited state contributes considerably to the overall magnetic moment of **1a** at 5 °C, as the observed value of *χ*_M_ *T* = 0.93 cm^3^ K mol^–1^ is significantly higher than the theoretical value of 0 cm^3^ K mol^–1^ for a solely populated *S* = 0 ground state. Analogously, a mixture of low-spin and high-spin Fe^II^ centers is present at 60 °C, as evident from the significant deviation of *χ*_M_ *T* = 1.99 cm^3^ K mol^–1^ from the average value of the high-spin analogue **2a**. Considering a value of *χ*_M_ *T* = 0 cm^3^ K mol^–1^ for a solely populated *S* = 0 low-spin state and *χ*_M_ *T* = 3.63 cm^3^ K mol^–1^ for a solely populated *S* = 2 high-spin state with *g* = 2.20, the high-spin molar fraction of Fe^II^ centers in **1a** was calculated as a function of temperature (see Fig. S14†). A linear fit to the data gives *T*_1/2_ = 325(1) K or 52(1) °C. Moreover, the data were simulated using the regular solution model^36,37^ to estimate thermodynamic parameters of Δ*H* = 18.0(3) kJ mol^–1^ and Δ*S* = 55.5(9) J K^–1^ mol^–1^, which are similar in magnitude to related mononuclear spin-crossover Fe^II^ complexes (see Fig. S15†).^15,28c,36,38^

To test our hypothesis that the low-spin state of [Fe(L_1_)]^2+^ in **1a** is stabilized in H_2_O relative to CH_3_CN, variable-temperature magnetic susceptibility data were collected for an acetonitrile solution of **1a**, using the same procedure as described above (see Fig. S16†). As observed in aqueous solution, *χ*_M_ *T* increases nearly linearly with increasing temperature, from 0.62 cm^3^ K mol^–1^ at –42 °C to 2.71 cm^3^ K mol^–1^ at 60 °C. Furthermore, a linear fit to the data affords *T*_1/2_ = 17(1) °C, which is 35 °C lower than observed in H_2_O, and demonstrates the different donor strengths of the H_2_O and CH_3_CN (see Fig. S17†).

### Variable-temperature NMR spectroscopy

To further investigate the solution properties of compounds **1a**, **1b**, **2a**, **2b**, and **3a**, variable-temperature ^1^H NMR spectra were collected in CD_3_CN at selected temperatures. The ^1^H NMR spectra of compounds **1b**, **2b**, and **3a** resemble those of their respective ligands (see Fig. S18–S20†) and show minimal changes in the temperature range 25–56 °C, confirming diamagnetic electronic structures (see Fig. S21–S23†). In contrast, the ^1^H NMR spectra of **2a** display nine paramagnetically shifted resonances, consistent with time-averaged *C*_3_ symmetry in CH_3_CN solution (see Fig. S24†). At –1 °C, these resonances span –18 to 225 ppm, typical for high-spin Fe^II^ complexes.^10,12,21b,d,e,g,h,28c^ As the temperature is increased to 56 °C, the peaks shift linearly toward the diamagnetic region. This Curie behavior (*δ* ∝ *T* ^–1^) is characteristic of high-spin complexes and confirms that **2a** remains *S* = 2 over the entire temperature range. In contrast, the ^1^H NMR resonances of **1a** show anti-Curie behavior, shifting away from the diamagnetic region with increasing temperature (see Fig. S25†). Specifically, at –38 °C, the proton resonances are dispersed between –2 and 13 ppm, barely beyond the diamagnetic region, suggesting primary population of an *S* = 0 ground state. Increasing the temperature to 56 °C results in an expansion of the chemical shift range to –25–150 ppm, indicative of thermal population of the high-spin excited state. An analogous trend is observed in the variable-temperature ^1^H NMR spectra of **1a** in D_2_O, though the resonances are broader and less shifted than in CD_3_CN at analogous temperatures, giving a chemical shift range from –17 to 107 ppm at 56 °C (see Fig. S26†). These observations are consistent with the higher *T*_1/2_ in H_2_O relative to CH_3_CN, as evident from solution magnetic measurements and UV-vis data.

**Fig. 5:**
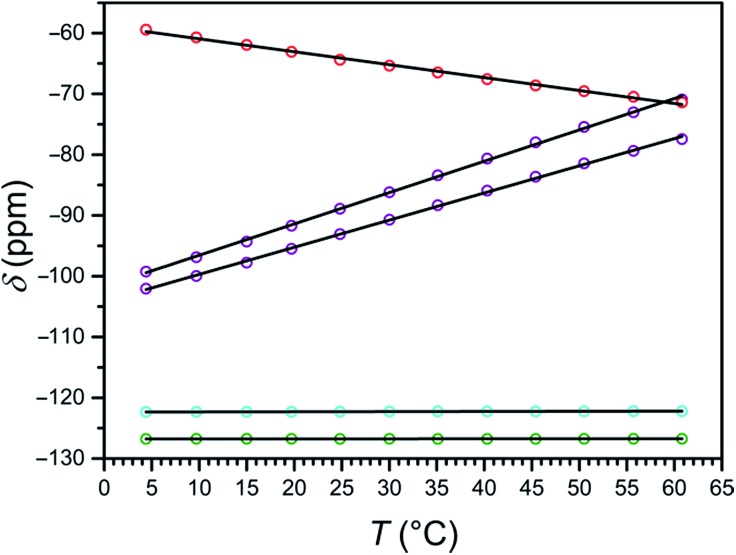
Plot of the temperature dependence of the ^19^F NMR chemical shift of **1a** (purple), **1b** (cyan), **2a** (red), and **2b** (green) in H_2_O. Chemical shift values are corrected with TFE internal standard and referenced to CFCl_3_. Solid black lines represent linear fits to the data.

In order to determine the effect of spin state on ^19^F resonances, and to assess these compounds as candidates for ^19^F MRS thermometry, we collected variable-temperature ^19^F NMR spectra for aqueous solutions of **1a** and **2a** from 4 to 61 °C, using trifluoroethanol (TFE) as an internal standard (see Experimental section, Fig. S27, and Table S2†). To better understand how the temperature dependence of ^19^F NMR chemical shifts is affected by the electronic spin state, and to quantify the hyperfine shifts of the paramagnetic Fe^II^ compounds **1a** and **2a**, their corresponding Zn^II^ analogues, **1b** and **2b**, were employed as diamagnetic references (see Table 2).^18*c*^ Importantly, the chemical shifts of the fluorine resonances of Zn^II^ compounds **1b** and **2b** are effectively invariant to temperature changes (see Fig. 5, S28, and S29†).

**Table 2:** Summary of ^19^F NMR properties for compounds **1a** and **2a** in CD_3_CN, H_2_O, and FBS solutions

	CD_3_CN			H_2_O			FBS		
	**1a**		**2a**	**1a**		**2a**	**1a**		**2a**
*δ* (ppm)^*a*^	59.4	52.6	55.9	41.6	36.3	59.2	40.7	35.5	59.0
Δ*δ* (ppm)	+40.9^*b*^	+36.2^*b*^	–13.6^*b*^	+28.3^*c*^	+24.6^*c*^	–12.0^*c*^	+28.8^*c*^	+25.1^*c*^	–11.7^*c*^
CT (ppm per °C)	+0.67(2)^*b*^	+0.59(2)^*b*^	–0.24(2)^*b*^	+0.52(1)^*c*^	+0.45(1)^*c*^	–0.21(1)^*c*^	+0.52(1)^*c*^	+0.45(1)^*c*^	–0.21(1)^*c*^
FWHM (Hz)^*d*^	287	270	105	282	243	868	251	241	872
|CT|/FWHM (per °C)	1.10	1.03	1.07	0.87	0.87	0.11	0.97	0.88	0.11

^*a*^Referenced to corresponding Zn^II^ analogues at 40 °C.

^*b*^Obtained from the temperature range –22–40 °C.

^*c*^Obtained from the temperature range 4–61 °C.

^*d*^Obtained from data at 40 °C.

At 4 °C, the ^19^F NMR spectrum of the high-spin compound **2a** displays a single resonance at –59.4 ppm *vs.* CFCl_3_ that is shifted +67.3 ppm from its diamagnetic Zn^II^ analogue **2b**. As the temperature is raised to 61 °C, the chemical shift of the paramagnetic signal shifts upfield to –71.4 ppm, closer to the ^19^F resonance of its diamagnetic analogue, as expected for Curie behavior (see Fig. S30 and S31, and Tables S3 and S4†). The observation of a single signal for **2a** further supports the *C*_3_ symmetry of the [Fe(L_2_)]^2+^ cation in solution, as suggested by ^1^H NMR spectroscopy. Analysis of the temperature dependence of the ^19^F NMR chemical shift reveals a linear temperature dependence over 4–61 °C following the equation *δ*_ppm_ = –0.21 × *T* – 58.8, affording a temperature coefficient^39^ of CT = –0.21(1) ppm per °C (see Fig. 5, and Table 2). Since linewidth has a significant effect on the precision of MRS probes, the value |CT|/FWHM (FWHM = full width at half maximum) is also a useful measure of probe sensitivity. At 40 °C, the fluorine resonance of **2a** exhibits a FWHM of 868 Hz, giving a |CT|/FWHM = 0.11 per °C.

The ^19^F NMR spectrum of **1a** obtained at 4 °C exhibits two resonances of equal intensity at –99.3 and –102.1 ppm *vs.* CFCl_3_ (see Fig. S32, and Table S3†), suggesting that the two 3-fluoro-2-picolyl arms of L_1_ are inequivalent on the NMR timescale. These peaks are shifted +23.1 and +20.3 ppm from the diamagnetic Zn^II^ analogue **1b** (see Fig. S33, and Table S4†), which exhibits two overlapping resonances centered at –122.3 ppm (see Fig. S28†). Increasing the temperature to 61 °C results in a downfield shift of the resonances of **1a** to +51.3 and +44.8 ppm from **1b**, consistent with the anti-Curie behavior observed in the corresponding ^1^H NMR spectra. The ^19^F chemical shift of both resonances for **1a** vary linearly between 4 and 61 °C following the equations *δ*_ppm_ = 0.52 × *T* – 101.7 and *δ*_ppm_ = 0.45 × *T* – 104.2, providing temperature sensitivities of CT = +0.52(1) and +0.45(1) ppm per °C, respectively (see Fig. 5, and Table 2). Fluorine resonances with the narrowest linewidths are obtained at 20 °C, but the peaks broaden significantly above 55 °C (FWHM > 500 Hz). At 40 °C, the fluorine resonances each shows a value of |CT|/FWHM = 0.87 per °C.

The two ^19^F NMR resonances of **1a** exhibit 2.5- and 2.1-fold higher CT values than that of the high-spin **2a**. Furthermore, the narrower linewidths of the resonances of **1a** afford an 8-fold higher |CT|/FWHM value than **2a** at 40 °C. Remarkably, the two ^19^F resonances of **1a** represent 43- and 38-fold enhancement of temperature sensitivity compared to diamagnetic perfluorocarbons that have been employed for *in vivo* thermometry.^20^ Despite the much narrower peak widths of the diamagnetic fluorine resonances relative to those of **1a**, the |CT|/FWHM value of **1a** at 40 °C is 2.9-fold higher owing to the strong temperature dependence of the chemical shift of its two resonances. These observations demonstrate that the use of spin-crossover complexes may provide an excellent strategy for improving the sensitivity of ^19^F MR thermometers.

Furthermore, the separation between the two fluorine resonances of **1a** varies strongly with temperature, from 2.81 ppm at 4 °C to 6.52 ppm at 61 °C, following the linear relationship Δ*δ*_ppm_ = 0.069 × *T* + 2.47 (see Fig. S34†). This peak separation provides an internal method of correcting errors in the ^19^F chemical shift that arise from complicating physiological effects, such as motion, magnetic susceptibility changes, and varying oxygen tension.^20^ Overall, three temperature-dependent parameters of compound **1a** can be followed for MR thermometry, namely the ^19^F NMR chemical shifts of two inequivalent fluorine substituents, and the chemical shift difference between these signals.

**Fig. 6:**
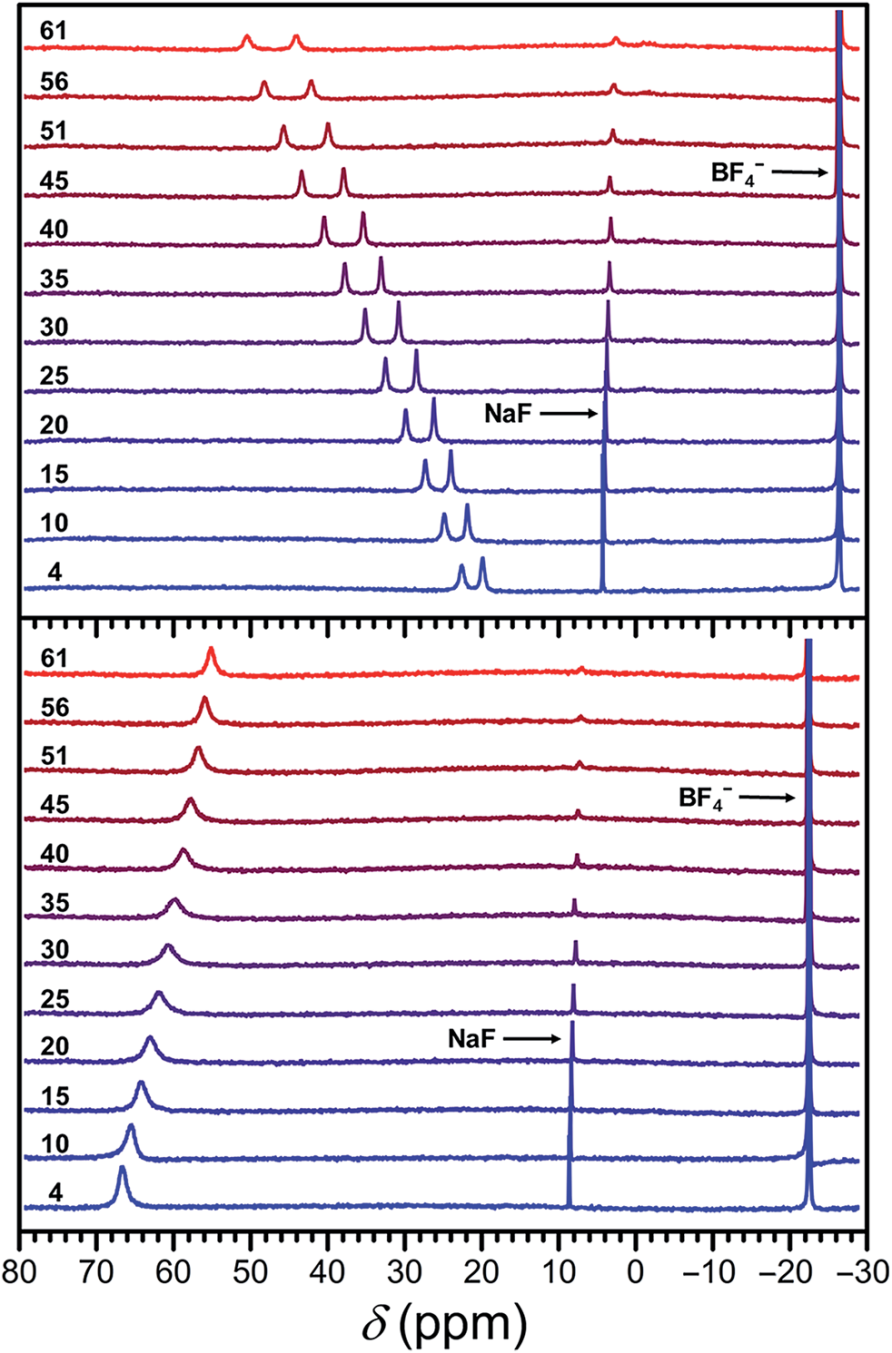
Variable-temperature ^19^F NMR spectra of **1a** (upper) and **2a** (lower) in FBS, using a NaF internal standard. The chemical shifts of the Fe^II^ compounds **1a** and **2a** are referenced to their corresponding Zn^II^ analogues **1b** and **2b**, set to 0 ppm. Black numbers correspond to temperature in °C.

To evaluate the efficacy of **1a** and **2a** in a physiological environment, ^19^F NMR spectra were collected from 4 to 61 °C on 13.4 and 15.0 mM solutions of **1a** and **2a**, respectively, in fetal bovine serum (FBS), using NaF as an internal standard (see Fig. S35†). The ^19^F NMR spectra in FBS are essentially identical to those recorded in H_2_O and provide the same CT values (see Fig. S36 and S37 and Tables 2 and S5†). Plots of the temperature dependence of fluorine chemical shifts of compounds **1a** and **2a** in FBS are depicted in Fig. 6, where the chemical shifts of the Fe^II^ complexes have been referenced to the corresponding shifts of Zn^II^ analogues **1b** and **2b** in water (see Table S6†). The linewidths for the resonance of **2a** are similar in FBS and H_2_O, while **1a** exhibits slightly narrower peaks in the high-temperature region (>30 °C) in FBS compared to those in H_2_O, resulting in higher |CT|/FWHM values in FBS. Furthermore, both complexes remain intact while incubated with FBS for over 24 h, as evidenced by identical ^19^F NMR spectra recorded at 25 °C initially and after 24 h (see Fig. S38 and S39†). Taken together, these results demonstrate the stability of compounds **1a** and **2a** in a physiological environment and indicate that temperature measurements with +0.52(1) and –0.21(1) ppm per °C sensitivity, respectively, can be achieved with these probes through chemical shift ^19^F MR thermometry. Moreover, the excellent stability and favorable ^19^F MR properties of **1a** under physiological conditions suggest that this compound is a viable candidate for *in vivo* studies.

A comparison of the ^19^F NMR properties of compounds **1a** and **2a** in CD_3_CN (see Fig. S40–S44†), H_2_O and FBS is summarized in Table 2. The hyperfine shift of the spin-crossover compound **1a** is significantly affected by the solvent, in contrast to high-spin **2a** (see Tables S3 and S7†). Along these lines, the resonances of **1a** display a 1.3-fold higher temperature sensitivity in CD_3_CN than in H_2_O, which is consistent with a lower *T*_1/2_ in CD_3_CN. These observations reflect the pronounced effects of spin state on ^19^F NMR chemical shift, as has been previously reported for transition metal porphyrin complexes.^18^ Nevertheless, the results presented here provide a rare examination of spin state effects on ^19^F NMR spectra across a series of metal complexes.

## Conclusions

The foregoing results demonstrate the potential utility of paramagnetic Fe^II^ complexes as chemical shift ^19^F MR thermometers. Most importantly, we show that the sensitivity of ^19^F MR thermometers can be improved by employing a temperature-dependent change in spin state, as illustrated in a series of Fe^II^ complexes. To our knowledge, these complexes represent the first examples of paramagnetic ^19^F MR chemical shift agents proposed for thermometry applications. Future efforts will focus on *in vitro* and *in vivo* MRS thermometry experiments on these compounds and the synthesis of spin-crossover complexes with higher sensitivity by exploiting the chemical tunability of the tacn-based ligand scaffold.
