# Supplementary material for: Spin-crossover and high-spin iron(ii) complexes as chemical shift 19F magnetic resonance thermometers
Source: Chem Sci. 2017 Jan 6;8(3):2448–56. doi: 10.1039/c6sc04287b (PMC5477811; doi:10.1039/c6sc04287b)
Supplement: Supplementary file 1 [file SC-008-C6SC04287B-s001.pdf]

Electronic Supplementary Material (ESI) for Chemical Science.
This journal is © The Royal Society of Chemistry 2017

Electronic Supplementary Information for:

Spin-Crossover and High-Spin Iron(II) Complexes as Chemical Shift ^{19}F Magnetic Resonance Thermometers

Agnes E. Thorarinsdottir, Alexandra I. Gaudette and T. David Harris*

Department of Chemistry, Northwestern University, 2145 Sheridan Road, Evanston IL USA 60208-3113
e-mail: dharris@northwestern.edu

Chemical Science

Table of Contents

Experimental Section **S3**
Scheme S1: Synthesis of ligands L_x (x = 1–3) **S11**
Table S1: Crystallographic data for **1a**·$0.5CH_3CN$, **1b**, **2a**, **2b**, and **3a** **S12**
Figure S1: Crystal structure of $[Fe(L_3)]^{2+}$ at 100 K **S13**
Figure S2: Variable-temperature UV/Visible spectra of **1a** in MeCN **S14**
Figure S3: Variable-temperature UV/Visible spectra of **2a** in MeCN **S15**
Figure S4: Variable-temperature UV/Visible spectra of **3a** in MeCN **S16**
Figure S5: Variable-temperature UV/Visible spectra of **1b** in MeCN **S17**
Figure S6: Variable-temperature UV/Visible spectra of **2b** in MeCN **S18**
Figure S7: Comparison of UV/Visible spectra of **1a**, **2a**, and **3a** in MeCN at −35 °C **S19**
Figure S8: Comparison of UV/Visible spectra of **1a**, **2a**, and **3a** in MeCN at 65 °C **S20**
Figure S9: UV/Visible spectra of **1a** in H_2O **S21**
Figure S10: UV/Visible spectra of **2a** in H_2O **S22**
Figure S11: UV/Visible spectra of **3a** in H_2O **S23**
Figure S12: UV/Visible spectra of **1b** in H_2O **S24**
Figure S13: UV/Visible spectra of **2b** in H_2O **S25**
Figure S14: Plot of high-spin molar fraction of **1a** vs temperature in H_2O **S26**
Figure S15: Thermodynamic data for the spin transition of **1a** in H_2O **S27**
Figure S16: Comparison of magnetic susceptibility of **1a** vs temperature in H_2O and MeCN **S28**
Figure S17: Comparison of high-spin molar fraction of **1a** vs temperature in H_2O and MeCN **S29**
Figure S18: ^1H NMR spectrum of L_1 in $CDCl_3$ at 25 °C **S30**
Figure S19: ^1H NMR spectrum of L_2 in $CDCl_3$ at 25 °C **S31**
Figure S20: ^1H NMR spectrum of L_3 in $CDCl_3$ at 25 °C **S32**

Figure S21: Variable-temperature ^{1}H NMR spectra of **1b** in CD_3CN S33
Figure S22: Variable-temperature ^{1}H NMR spectra of **2b** in CD_3CN S34
Figure S23: Variable-temperature ^{1}H NMR spectra of **3a** in CD_3CN S35
Figure S24: Variable-temperature ^{1}H NMR spectra of **2a** in CD_3CN S36
Figure S25: Variable-temperature ^{1}H NMR spectra of **1a** in CD_3CN S37
Figure S26: Variable-temperature ^{1}H NMR spectra of **1a** in D_2O S38
Figure S27: Plot of the temperature dependence of ^{19}F NMR chemical shift of TFE in H_2O S39
Table S2: ^{19}F NMR chemical shifts of the internal standards TFE and NaF in H_2O S40
Figure S28: Variable-temperature ^{19}F NMR spectra of **1b** in 2.1 mM TFE(aq) S41
Figure S29: Variable-temperature ^{19}F NMR spectra of **2b** in 2.1 mM TFE(aq) S42
Figure S30: Variable-temperature ^{19}F NMR spectra of **2a** in 2.1 mM TFE(aq) S43
Figure S31: Variable-temperature ^{19}F NMR spectra of **2a** in 2.1 mM TFE(aq) referenced to **2b** S44
Figure S32: Variable-temperature ^{19}F NMR spectra of **1a** in 2.1 mM TFE(aq) S45
Figure S33: Variable-temperature ^{19}F NMR spectra of **1a** in 2.1 mM TFE(aq) referenced to **1b** S46
Table S3: Summary of ^{19}F NMR properties of **1a**, **1b**, **2a**, and **2b** in 2.1 mM TFE(aq) S47
Table S4: ^{19}F NMR chemical shifts of **1a** and **2a** in 2.1 mM TFE(aq) referenced to **1b** and **2b** S48
Figure S34: Plot of ^{19}F NMR chemical shift separation vs temperature for **1a** in 2.1 mM TFE(aq) S49
Figure S35: Variable-temperature ^{19}F NMR spectra of a solution of TFE and NaF in H_2O S50
Figure S36: Variable-temperature ^{19}F NMR spectra of **1a** in FBS S51
Figure S37: Variable-temperature ^{19}F NMR spectra of **2a** in FBS S52
Table S5: Summary of ^{19}F NMR properties of **1a** and **2a** in FBS S53
Table S6: ^{19}F NMR chemical shifts of **1a** and **2a** in FBS referenced to **1b** and **2b** S54
Figure S38: Comparison of ^{19}F NMR spectra of **1a** in FBS at 25 °C at different times S55
Figure S39: Comparison of ^{19}F NMR spectra of **2a** in FBS at 25 °C at different times S56
Figure S40: Variable-temperature ^{19}F NMR spectra of **1a** in CD_3CN S57
Figure S41: Variable-temperature ^{19}F NMR spectra of **2a** in CD_3CN S58
Figure S42: Variable-temperature ^{19}F NMR spectra of **1b** in CD_3CN S59
Figure S43: Variable-temperature ^{19}F NMR spectra of **2b** in CD_3CN S60
Table S7: Summary of ^{19}F NMR properties of **1a**, **1b**, **2a**, and **2b** in CD_3CN S61
Figure S44: Plot of ^{19}F NMR chemical shift separation vs temperature for **1a** in CD_3CN S62
References S63

Experimental Section

General Considerations. Unless otherwise specified, the manipulations described below were carried out at ambient atmosphere and temperature. Air- and water-free manipulations were performed under a dinitrogen atmosphere in a Vacuum Atmospheres Nexus II glovebox or using a standard Schlenk line. Glassware was oven-dried at 150 °C for at least 4 h and allowed to cool in an evacuated antechamber prior to use in the glovebox. Acetonitrile (MeCN), dichloromethane (CH_2Cl_2), diethyl ether (Et_2O) and methanol (MeOH) were dried using a commercial solvent purification system from Pure Process Technology and stored over 3 or 4 Å molecular sieves prior to use. Water was obtained from a purification system from EMD Millipore. Elemental analysis was conducted by Midwest Microlab Inc. Deuterated solvents were purchased from Cambridge Isotope Laboratories and stored over 3 or 4 Å molecular sieves prior to use. The compounds Ts_3-dient (*N,N′,N″*-tri(p-toluenesulfonyl)-diethylenetriamine) and Ts_2-glycol (1,2-di(p-toluenesulfonyloxy)ethane) were synthesized following literature procedures.[1] All other chemicals and solvents were purchased from commercial vendors and used without further purification.

Synthesis of *N,N′,N″*-tritosyl-1,4,7-triazacyclononane (Ts_3-tacn).

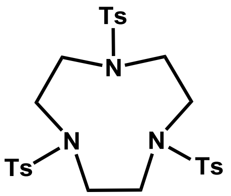

This compound was synthesized following a modified literature procedure.[2] Ts_3-dient (14.2 g, 25.2 mmol) and cesium carbonate (17.2 g, 52.8 mmol) were stirred vigorously in DMF (135 mL) at 25 °C for 1 h. To this white suspension, a solution of Ts_2-glycol (9.30 g, 25.2 mmol) in DMF (60 mL) was added dropwise. The resulting pale yellow mixture was stirred at ambient temperature for 12 h. After that time the color of the reaction mixture had turned orange. The mixture was filtered and the reddish orange filtrate added slowly to deionized water (800 mL) to give the product as a white precipitate. The solid was collected by vacuum filtration and suspended in DMF/H_2O 1:1 mixture (90 mL) and stirred for 3 h to wash. The resulting white solid was collected by vacuum filtration, washed thoroughly with deionized water and dried under vacuum for 6 h. The crude product was recrystallized from CH_2Cl_2/ethanol to give the title compound as colorless needles (12.9 g, 87 %). ^{1}H NMR (400 MHz, $CDCl_3$, 25 °C): δ 7.70 (d, J = 6.6 Hz, 6H), 7.33 (d, J = 6.8 Hz, 6H), 3.42 (s, 12 H), 2.43 (s, 9 H).

Synthesis of *N*-monotosyl-1,4,7-triazacyclononane (H_2Ts-tacn).

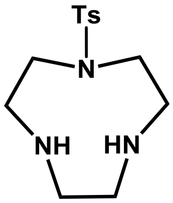

This compound was synthesized following a modified literature procedure.[2] Ts_3-tacn (3.51 g, 5.91 mmol) and phenol (4.56 g, 47.3 mmol) were introduced into a 250 mL round bottom flask connected to a reflux condenser. To this, a solution of 33 % HBr in acetic acid (36 mL) was slowly added. The orange-colored reaction mixture was carefully heated to 90 °C under a dinitrogen atmosphere and stirred for 16 h. During that time a white precipitate of H_2Ts-tacn·2HBr was formed. The orange suspension was cooled to ambient temperature and then filtered through a fritted glass funnel. The white precipitate was washed with glacial acetic acid (10 mL), dissolved in deionized water (50 mL) and basified with 1 M aqueous NaOH solution (50 mL) to give a clear solution. This solution was extracted with $CHCl_3$ (3 × 50 mL) and the colorless extracts were combined, dried over $MgSO_4$(s) and filtered. The product was collected as a white powder after evaporating the chloroform solvent to dryness (1.11 g, 66 %). ^{1}H NMR (400 MHz, $CDCl_3$, 25 °C): δ 7.63 (d, J = 7.9 Hz, 2H), 7.25 (d, J = 8.0 Hz, 2H), 3.13 (br s, 4 H), 3.02 (br s, 4 H), 2.83 (s, 4H), 2.36 (s, 3 H), 1.91 (s, 2H).

Synthesis of 3-fluoro-2-formyl-6-methylpyridine.

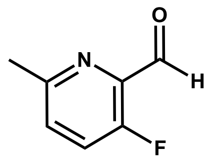

This compound was synthesized following a modified literature procedure.[3] Under a dinitrogen atmosphere, 2-bromo-3-fluoro-6-methylpyridine (3.73 g, 19.6 mmol) was dissolved in dry toluene (30 mL) and that colorless solution was added dropwise to a stirred solution of *n*-BuLi (1.6 M in hexanes, 12.3 mL, 19.6 mmol) in dry toluene (30 mL) at −78 °C. The resulting reddish-orange solution was stirred at −78 °C for 1 h and then anhydrous DMF (4.30 g, 58.9 mmol) was slowly added to give a bright red solution. That solution was stirred at −78 °C for additional 2 h, then warmed to ambient temperature and quenched with water (50 mL). The bright yellow aqueous layer was extracted with EtOAc (2 × 200 mL), the combined organic layer was washed with saturated aqueous NaCl solution (100 mL), dried over $MgSO_4$(s) and filtered. Removing the solvent under reduced pressure afforded a red oil which was dissolved in a minimum amount of EtOAc (15 mL) and eluted through a plug of celite. The red-colored solution was concentrated in vacuo and further dried under vacuum for 12 h to afford the product as a reddish crystalline solid (2.23 g, 80 %). ^{1}H NMR (400 MHz, $CDCl_3$, 25 °C): δ 10.21 (s, 1H), 7.48 (dd, J = 7.5, 7.2 Hz, 1H), 7.41 (dd, J = 6.9, 3.0 Hz, 1H), 2.66 (s, 3H). ^{19}F NMR (376 MHz, $CDCl_3$, 25 °C): δ −129.75 (dd, J = 9.7, 3.9 Hz).

General procedure for the synthesis of *N,N′*-di(2-picolyl)-*N″*-monotosyl-1,4,7-triazacyclononane.

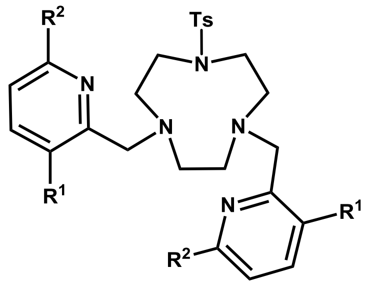

R[1] = F
R[2] = Me, H

A 50 mL Schlenk flask was charged with 1.0 equivalent of H_2Ts-tacn and a magnetic stir bar. To this, dry CH_2Cl_2 (20 mL) was added to solubilize the ligand precursor completely to give a light yellow solution. Under a dinitrogen atmosphere, 2.5 equivalents of a 2-pyridinecarboxaldehyde derivative was added as a solid in a single portion. After stirring the mixture at ambient temperature for 1 h, 5.0 equivalents of sodium triacetoxyborohydride was added and the resulting suspension was heated to 40 °C and refluxed under a dinitrogen atmosphere for 1–3 h. The reaction was monitored by ESI-MS in CH_2Cl_2 solvent. When no remaining starting material was observed, the reaction mixture was cooled to ambient temperature and evaporated to dryness under reduced pressure. The crude residue was partioned between 2 M aqueous NaOH solution (20 mL) and $CHCl_3$ (40 mL), and the organic phase was collected, dried over $MgSO_4$(s), filtered and concentrated in vacuo. The obtained oil was washed with 6 M aqueous NaOH solution (15 mL) by stirring for 1–2 h at ambient temperature, and then extracted into $CHCl_3$ (25 mL). The organic phase was dried over $MgSO_4$(s) and filtered, and the $CHCl_3$ solvent was removed under reduced pressure to afford the product as an oil or an oily solid which was used in a subsequent step after drying under vacuum for 6 hours.

Synthesis of *N,N′*-di(3-fluoro-2-picolyl)-*N″*-monotosyl-1,4,7-triazacyclononane.

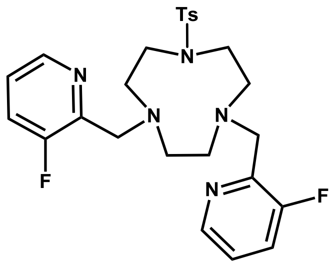

According to the general procedure, *N,N′*-di(3-fluoro-2-picolyl)-*N″*-monotosyl-1,4,7-triazacyclononane was prepared as a pale yellow oil in near quantitative yield from H_2Ts-tacn (0.361 g, 1.28 mmol), 3-fluoro-2-formylpyridine (0.399 g, 3.19 mmol) and sodium triacetoxyborohydride (1.36 g, 6.40 mmol). ESI-MS m/z: calcd for $C_{25}H_{30}F_2N_5O_2S$ $(M+H)^+$ 502.21, found 502.18. ^{1}H NMR (400 MHz, $CDCl_3$, 25 °C): δ 8.33 (dd, J = 3.7, 1.1 Hz, 2H), 7.63 (d, J = 6.6 Hz,

2H), 7.33 (ddd, J = 7.2, 7.2, 1.1 Hz, 2H), 7.26 (d, J = 6.2 Hz, 2H), 7.24–7.27 (m, 2 H), 7.17–7.20 (m, 2H), 3.88 (d, J = 1.8 Hz, 4H), 3.21–3.24 (m, 4H), 3.07–3.10 (m, 4H), 2.76 (s, 4H), 2.40 (s, 3H). ^{19}F NMR (376 MHz, $CDCl_3$, 25 °C): δ −123.83 (m).

Synthesis of *N,N′*-di(3-fluoro-6-methyl-2-picolyl)-*N″*-monotosyl-1,4,7-triazacyclononane.

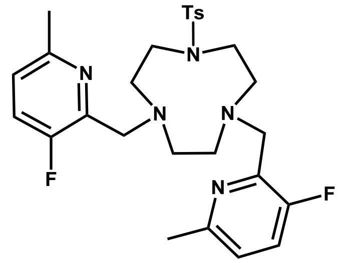

According to the general procedure, *N,N′*-di(3-fluoro-6-methyl-2-picolyl)-*N″*-monotosyl-1,4,7-triazacyclononane was prepared as a reddish-brown oil in near quantitative yield from H_2Ts-tacn (0.459 g, 1.64 mmol), 3-fluoro-2-formyl-6-methylpyridine (0.570 g, 4.10 mmol) and sodium triacetoxyborohydride (1.74 g, 8.20 mmol). ESI-MS m/z: calcd for $C_{27}H_{34}F_2N_5O_2S$ $(M+H)^+$ 530.24, found 530.21. ^{1}H NMR (400 MHz, $CDCl_3$, 25 °C): δ 7.64 (d, J = 8.2 Hz, 2H), 7.26 (dd, J = 7.8 Hz, 2H), 7.20 (dd, J = 8.4, 8.4 Hz, 2H), 7.00 (dd, J = 8.4, 3.7 Hz, 2H), 3.82 (d, J = 2.2 Hz, 4H), 3.23–3.27 (m, 4H), 3.05–3.08 (m, 4H), 2.72 (s, 4H), 2.48 (s, 6H), 2.40 (s, 3H). ^{19}F NMR (376 MHz, $CDCl_3$, 25 °C): δ −129.85 (d, J = 9.4 Hz).

General procedure for the synthesis of *N,N′*-di(2-picolyl)-1,4,7-triazacyclononane.

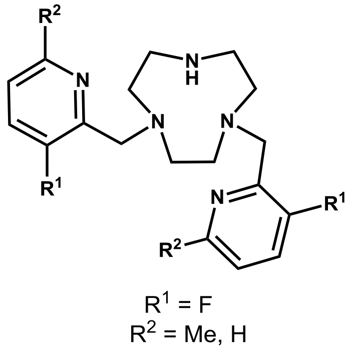

The monotosylated ligand precursor and a magnetic stir bar were introduced into a 25 mL round bottom flask. To this, concentrated sulfuric acid solution (10 mL) was slowly added and the resulting orangish mixture was purged under vacuum for 1 h. Afterwards, the reaction flask was connected to a reflux condenser and the homogeneous light orangish solution was heated to 120 °C under a dinitrogen atmosphere and left stirring at that temperature for 16 h. The resulting black reaction mixture was allowed to reach ambient temperature and then added to stirring cold ethanol (60 mL). Addition of Et_2O (300 mL) resulted in the formation of a whitish suspension, which was filtered to give a greyish oily solid. This compound was dissolved in 2 M aqueous NaOH solution (50 mL), yielding a faint brown-colored solution. The aqueous layer was extracted with $CHCl_3$ (3 × 100 mL) and the pale yellow extracts were combined, dried over $MgSO_4$(s), filtered and concentrated under reduced pressure to afford the product as an oil. The collected oil was dried under vacuum for 6 h before proceeding to the next step.

Synthesis of *N,N′*-di(3-fluoro-2-picolyl)-1,4,7-triazacyclononane.

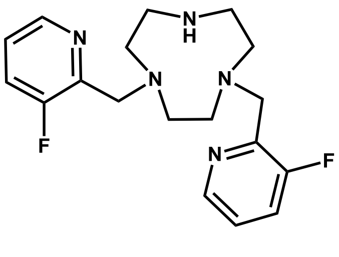

According to the general procedure, *N,N′*-di(3-fluoro-2-picolyl)-1,4,7-triazacyclononane was prepared as a yellow-orangish oil (0.328 g) from *N,N′*-di(3-fluoro-2-picolyl)-*N″*-monotosyl-1,4,7-triazacyclononane. ESI-MS m/z: calcd for $C_{18}H_{24}F_2N_5$ $(M+H)^+$ 348.20, found 348.18. ^{1}H NMR (400 MHz, $CDCl_3$, 25 °C): δ 8.35 (ddd, J = 5.0, 1.1 Hz, 2H), 7.36 (dd, J = 7.4, 7.4 Hz, 2H), 7.19–7.24 (m, 2 H), 3.91 (d, J = 1.6 Hz, 4H), 2.76–2.80 (m, 4H), 2.76 (s, 4H), 2.57–2.61 (m, 4H). ^{19}F NMR (376 MHz, $CDCl_3$, 25 °C): δ −123.93.

Synthesis of *N,N′*-di(3-fluoro-6-methyl-2-picolyl)-1,4,7-triazacyclononane.

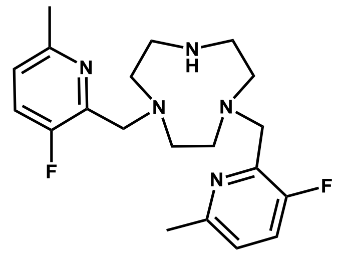

According to the general procedure, *N,N′*-di(3-fluoro-6-methyl-2-picolyl)-1,4,7-triazacyclononane was prepared as a reddish-brown oil (0.318 g, 59 %) from *N,N′*-di(3-fluoro-6-methyl-2-picolyl)-*N″*-monotosyl-1,4,7-triazacyclononane (0.762 g, 1.44 mmol). ESI-MS m/z: calcd for $C_{20}H_{28}F_2N_5$ $(M+H)^+$ 376.23, found 376.22. ^1H NMR (400 MHz, $CDCl_3$, 25 °C): δ 7.20 (dd, *J* = 8.9, 8.9 Hz, 2H), 7.00 (dd, *J* = 8.2, 3.5 Hz, 2 H), 3.84 (d, *J* = 2.0 Hz, 4H), 2.73–2.77 (m, 4H), 2.62 (s, 4H), 2.56–2.61 (m, 4H), 2.50 (s, 6H). ^{19}F NMR (376 MHz, $CDCl_3$, 25 °C): δ −129.93 (d, *J* = 9.0 Hz).

General procedure for the synthesis of *N,N′,N″*-tri(2-picolyl)-1,4,7-triazacyclononane.

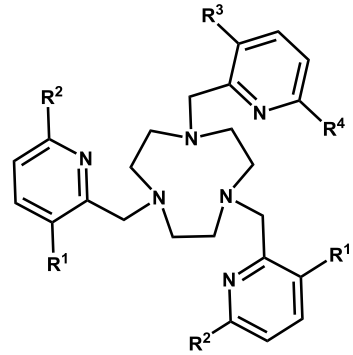

To a solution of 1.0 equivalent of a *N,N′*-di(2-picolyl)-1,4,7-triazacyclononane derivative in dry CH_2Cl_2 (15 mL), 1.2 equivalents of a 2-pyridinecarboxaldehyde derivative was added as a solid and the resulting mixture was stirred at ambient temperature under an atmosphere of dinitrogen for 1 h. After that time, 3.0 equivalents of sodium triacetoxyborohydride was added and the resulting suspension was heated to 40 °C and refluxed under dinitrogen atmosphere for 1–3 h. When the reaction was complete, as judged by ESI-MS analysis in CH_2Cl_2 solvent, the reaction mixture was allowed to reach ambient temperature and evaporated to dryness under vacuum. The crude residue was partioned between 2 M aqueous NaOH solution (20 mL) and $CHCl_3$ (40 mL), and the organic phase was collected, dried over $MgSO_4$(s) and filtered. Removal of the $CHCl_3$ solvent under reduced pressure afforded the product as an oil, which was stirred in 8 M aqueous NaOH solution (15 mL) for 1–2 h at ambient temperature to wash. The aqueous emulsion was then extracted with $CHCl_3$ (25 mL) and the organic phase was collected, dried over $MgSO_4$(s), filtered and concentrated. Drying under vacuum for 6 h afforded the ligands as oils or oily solids that were used in the metalation steps without further purification.

Synthesis of *N,N′*-di(3-fluoro-2-picolyl)-*N″*-mono(6-methyl-2-picolyl)-1,4,7-triazacyclononane (L_1).

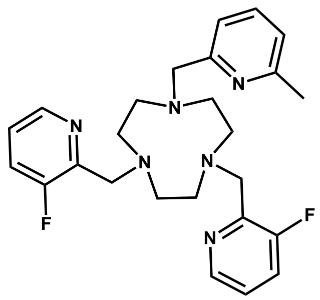

According to the general procedure, *N,N′*-di(3-fluoro-2-picolyl)-*N″*-mono(6-methyl-2-picolyl)-1,4,7-triazacyclononane was prepared as an yellow-orangish oil (0.232 g, 63 %) from *N,N′*-di(3-fluoro-2-picolyl)-1,4,7-triazacyclononane (0.192 g, 0.820 mmol), 2-formyl-6-methyl-pyridine (0.257 g, 2.05 mmol) and sodium triacetoxyborohydride (0.869 g, 4.10 mmol). ESI-MS m/z: calcd for $C_{25}H_{31}F_2N_6$ $(M+H)^+$ 453.26, found 453.25. ^1H NMR (400 MHz, $CDCl_3$, 25 °C): δ 8.34 (dd, *J* = 3.8, 1.1 Hz, 2H), 7.50 (dd, *J* = 6.1, 6.1 Hz, 1H), 7.33 (ddd, *J* = 7.1, 7.1, 1.1 Hz, 2H), 7.28 (d, *J* = 6.2 Hz, 1 H), 7.16–7.20 (m, 2H), 6.96 (d, *J* = 6.1 Hz, 1 H), 3.84 (d, *J* = 1.7 Hz, 4H), 3.73 (s, 2H), 2.91 (s, 4H), 2.86–2.89 (m, 4H), 2.72–2.76 (m, 4H), 2.50 (s, 3H). ^{19}F NMR (470 MHz, $CDCl_3$, 25 °C): δ −123.64 (d, *J* = 10.1 Hz). ^{19}F NMR (470 MHz, CD_3CN, 25 °C): δ −125.49 (s).

Synthesis of *N,N′,N″*-tri(3-fluoro-6-methyl-2-picolyl)-1,4,7-triazacyclononane (L_2).

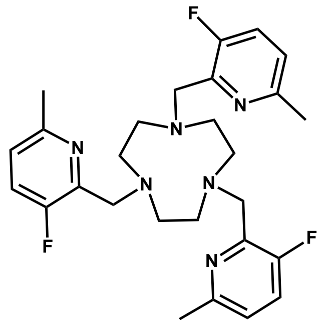

According to the general procedure, *N,N′,N″*-tri(3-fluoro-6-methyl-2-picolyl)-1,4,7-triazacyclononane was prepared as a reddish-brown oily solid (0.247 g, 58 %) from *N,N′*-di(3-fluoro-6-methyl-2-picolyl)-1,4,7-triazacyclononane (0.318 g, 0.850 mmol), 3-fluoro-2-formyl-6-methyl-pyridine (0.142 g, 1.02 mmol) and sodium triacetoxyborohydride (0.540 g, 2.55 mmol). ESI-MS m/z: calcd for $C_{27}H_{34}F_3N_6$ $(M+H)^+$ 499.28, found 499.25. ^1H NMR (500 MHz, $CDCl_3$, 25 °C): δ 7.20 (dd, J = 8.9, 8.9 Hz, 3H), 6.99 (dd, J = 8.4, 3.6 Hz, 3H), 3.76 (d, J = 2.1 Hz, 6H), 2.82 (s, 12H), 2.49 (s, 9H). ^{19}F NMR (470 MHz, $CDCl_3$, 25 °C): δ −129.59 (d, J = 9.4 Hz). ^{19}F NMR (470 MHz, CD_3CN, 25 °C): δ −131.53 (d, J = 9.2 Hz).

Synthesis of *N,N′,N″*-tri(3-fluoro-2-picolyl)-1,4,7-triazacyclononane (L_3).

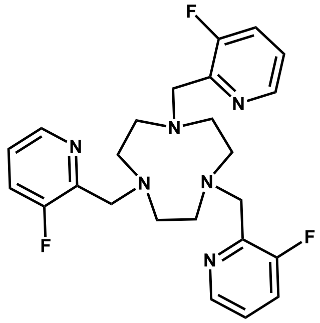

According to the general procedure, *N,N′,N″*-tri(3-fluoro-2-picolyl)-1,4,7-triazacyclononane was prepared as an orange oil (0.204 g, 47 %) from *N,N′*-di(3-fluoro-2-picolyl)-1,4,7-triazacyclononane (0.328 g, 0.944 mmol), 3-fluoro-2-formylpyridine (0.142 g, 1.13 mmol) and sodium triacetoxyborohydride (0.600 g, 2.83 mmol). ESI-MS m/z: calcd for $C_{24}H_{28}F_3N_6$ $(M+H)^+$ 457.23, found 457.18. ^1H NMR (400 MHz, $CDCl_3$, 25 °C): δ 8.34 (dd, J = 3.8, 1.1 Hz, 3H), 7.35 (ddd, J = 7.2, 7.2, 1.1 Hz, 3H), 7.17–7.23 (m, 3H), 3.82 (d, J = 1.7 Hz, 6H), 2.86 (s, 12H). ^{19}F NMR (376 MHz, $CDCl_3$, 25 °C): δ −123.70 (dd, J = 9.2, 3.4 Hz). ^{19}F NMR (470 MHz, CD_3CN, 25 °C): δ −125.56 (dd, J = 10.6, 1.4 Hz).

$[Fe(L_1)][BF_4]_2\cdot0.5CH_3CN$ (1a·0.5CH_3CN). Under an inert atmosphere of dinitrogen, a pale pink suspension of $Fe(BF_4)_2\cdot6H_2O$ (44.7 mg, 0.130 mmol) in MeCN (1.5 mL) was added dropwise to a stirring orange solution of L_1 (62.9 mg, 0.140 mmol) in MeCN (2.5 mL). During the addition, a color change to dark orange, then red, and finally to dark olive green was observed. The solution was stirred at ambient temperature for 1.5 h and then filtered. Vapor diffusion of Et_2O into the resulting dark olive green solution afforded dark red block-shaped crystals of **1a**·0.5CH_3CN (0.036 g, 39%) suitable for single-crystal X-ray diffraction analysis. The compound was placed under vacuum at ambient temperature for 24 h to give desolvated **1a**. Anal. Calcd. for $C_{25}H_{30}B_2F_{10}FeN_6$: C, 44.03%; H, 4.43%; N, 12.32%. Found: C, 43.90%; H, 4.60%; N, 12.39%. UV/Vis absorption spectra (5.9×10^{-5} M; H_2O, 25 °C): 263 nm (ε = 12400 M^{-1} cm^{-1}), 436 nm (ε = 5400 M^{-1} cm^{-1}); (5.5×10^{-5} M; MeCN, 25 °C): 264 nm (ε = 10700 M^{-1} cm^{-1}), 424 nm (ε = 2800 M^{-1} cm^{-1}). ESI-MS m/z: calcd for $C_{25}H_{30}F_2FeN_6$ $(M)^{2+}$ 254.09, found 253.96.

$[Zn(L_1)][BF_4]_2$ (1b). Under an inert atmosphere of dinitrogen, a colorless solution of $Zn(BF_4)_2\cdot5.3H_2O$ (11.8 mg, 0.035 mmol) in MeCN (1.5 mL) was added dropwise to a stirring dark orange solution of L_1 (16.7 mg, 0.037 mmol) in MeCN (2.5 mL). No distinct color change was observed during the addition of the metal salt, and the light orange solution was stirred at ambient temperature for 3 h and then filtered. Removal of the solvent under reduced pressure afforded a red-brown oily residue which was dissolved in a MeOH/MeCN 3:1 mixture (0.8 mL). Vapor diffusion of Et_2O into this dark red solution afforded a mixture of a polycrystalline colorless solid and a light orange solid that was washed with Et_2O. Colorless plate-shaped crystals of **1b** (0.011 g, 45%) suitable for single-crystal X-ray diffraction analysis were

grown from a concentrated dark orange solution of the polycrystalline solid (10 mg/mL) in MeOH/MeCN (2:1). Anal. Calcd. for $C_{25}H_{30}B_2F_{10}N_6Zn$: C, 43.42%; H, 4.37%; N, 12.15%. Found: C, 43.67%; H%, 4.52; N, 12.13%. UV/Vis absorption spectra (7.2 × 10^{-5} M; H_2O, 25 °C): 268 nm (ε = 11200 M^{-1} cm^{-1}); (8.8 × 10^{-6} M; MeCN, 25 °C): 268 nm (ε = 30500 M^{-1} cm^{-1}). ESI-MS m/z: calcd for $C_{25}H_{30}F_2N_6Zn$ $(M)^{2+}$ 258.09, found 257.98.

$[Fe(L_2)][BF_4]_2$ (2a). Under an inert atmosphere of dinitrogen, a pale pink suspension of $Fe(BF_4)_2$·$6H_2O$ (41.3 mg, 0.120 mmol) in MeCN (1.5 mL) was added dropwise to a stirring light orange solution of L_2 (64.1 mg, 0.130 mmol) in MeCN (3.5 mL). The resulting dark orange solution was stirred at ambient temperature for 3 h and then filtered. The dark orange filtrate was concentrated under reduced pressure to give a dark yellow solid. Vapor diffusion of Et_2O into a concentrated dark orange solution (10 mg/mL) of this solid in MeOH/MeCN (1:1) gave light yellow plate-shaped crystals of **2a** (0.042 g, 48%) suitable for single-crystal X-ray diffraction analysis. Anal. Calcd. for $C_{27}H_{36.2}B_2F_{11}FeN_6O_{1.6}$ (**2a**·$1.6H_2O$): C, 42.84%; H, 4.82%; N, 11.10%. Found: C, 43.00%; H, 4.76%; N, 11.00%. UV/Vis absorption spectra (6.9 × 10^{-5} M; H_2O, 25 °C): 276 nm (ε = 18800 M^{-1} cm^{-1}), 369 nm (ε = 1600 M^{-1} cm^{-1}); (5.5 × 10^{-5} M; MeCN, 25 °C): 273 nm (ε = 11100 M^{-1} cm^{-1}), 371 nm (ε = 800 M^{-1} cm^{-1}). ESI-MS m/z: calcd for $C_{27}H_{33}F_3FeN_6$ $(M)^{2+}$ 277.10, found 276.99.

$[Zn(L_2)][BF_4]_2$ (2b). Under an inert atmosphere of dinitrogen, a colorless solution of $Zn(BF_4)_2$·$5.3H_2O$ (20 mg, 0.059 mmol) in MeCN (1.5 mL) was added dropwise to a stirring light orange solution of L_2 (31 mg, 0.062 mmol) in MeCN (2.5 mL). The resulting light orange solution was stirred at ambient temperature for 3 h, then filtered and concentrated under vacuum to give a light brown oily solid. Colorless block-shaped crystals of **2b** (0.022 g, 51 %) suitable for single-crystal X-ray diffraction analysis were grown from a concentrated light orange solution of the solid (15 mg/mL) in MeOH/MeCN (1:1). Anal. Calcd. for $C_{27}H_{34.4}B_2F_{11}N_6O_{0.7}Zn$ (**2b**·$0.7H_2O$): C, 43.22%; H, 4.62%; N, 11.20%. Found: C, 43.26%; H, 4.76%; N, 11.20%. UV/Vis absorption spectra (3.0 × 10^{-5} M; H_2O, 25 °C): 278 nm (ε = 13400 M^{-1} cm^{-1}); (2.5 × 10^{-5} M; MeCN, 25 °C): 278 nm (ε = 35700 M^{-1} cm^{-1}). ESI-MS m/z: calcd for $C_{27}H_{33}F_3N_6Zn$ $(M)^{2+}$ 281.10, found 280.97.

$[Fe(L_3)][BF_4]_2$ (3a). Under an inert atmosphere of dinitrogen, a pale pink suspension of $Fe(BF_4)_2$·$6H_2O$ (42.6 mg, 0.126 mmol) in MeCN (1 mL) was added dropwise to a stirring yellow-orange solution of L_3 (63.4 mg, 0.139 mmol) in MeCN (2 mL) to give a dark red solution. This solution was stirred at ambient temperature for 1.5 h and then filtered. Removal of the solvent under reduced pressure yielded a red oily residue. Vapor diffusion of Et_2O into a concentrated dark red solution (10 mg/mL) of this compound in MeOH/MeCN (1:1) gave dark red hexagonal prism-shaped crystals of **3a** (0.046 g, 53%) suitable for single-crystal X-ray diffraction analysis. Anal. Calcd. for $C_{24}H_{27}B_2F_{11}FeN_6$: C, 42.02%; H, 3.97%; N, 12.25%. Found: C, 41.52%; H, 4.13%; N, 12.39%. UV/Vis absorption spectra (5.1 × 10^{-5} M; H_2O, 25 °C): 260 nm (ε = 13900 M^{-1} cm^{-1}), 437 nm (ε = 7900 M^{-1} cm^{-1}); (4.0 × 10^{-5} M; MeCN, 25 °C): 261 nm (ε = 17700 M^{-1} cm^{-1}), 436 nm (ε = 10600 M^{-1} cm^{-1}). ESI-MS m/z: calcd for $C_{24}H_{27}F_3FeN_6$ $(M)^{2+}$ 256.08, found 255.95.

X-ray structure determination. Single crystals of **1a**·$0.5CH_3CN$, **1b**, **2a**, **2b**, and **3a** were directly coated with Paratone-N oil, mounted on a MicroMounts™ rod and frozen under a stream of dinitrogen during data collection. The crystallographic data were collected at 100 K on a Bruker Kappa Apex II diffractometer equipped with an APEX-II detector and MoKα sealed tube source. Raw data were integrated and corrected for Lorentz and polarization effects with *SAINT* v8.34A.[4] Absorption corrections

were applied using the program SADABS.[5] Space group assignments were determined by examining systematic absences, E-statistics, and successive refinement of the structures. Structures were solved using direct methods in SHELXT and refined by SHELXL[6] operated within the OLEX2 interface.[7] All hydrogen atoms were placed at calculated positions using suitable riding models and refined using isotropic displacement parameters derived from their parent atoms. Thermal parameters for all non-hydrogen atoms were refined anisotropically. Crystallographic data for these compounds at 100 K and the details of data collection are listed in Table S1. Significant disorder of BF_4^- counterions was modelled in the crystal structures of **1a**·0.5CH_3CN and **3a**. However, only partial modelling of the disorder was achieved for **1a**·0.5CH_3CN, where the disordered BF_4^- ions accounted for the highest peaks in the difference Fourier map. Disordered lattice solvent molecules were present in the void space in the structures of compounds **2a** and **2b**, these species could not be identified and modelled properly. Therefore, they were treated as a diffuse contribution to the overall scattering without specific atom positions using the solvent masking procedure implemented in OLEX2. One of the two molecules in the asymmetric unit in the crystal structure of **1b** was severely disordered and had to be refined using restraints on all atoms. Positional disorder on the 2-picolyl groups was modelled with partial occupancies, where the sum of the occupancies of fluorine atoms was set equal to two, and the sum of methyl substituents was set equal to one.

^{1}H and ^{19}F NMR experiments. ^{1}H and ^{19}F NMR spectra of ligands L_x (x = 1–3) and ligand precursors were collected at 25 °C on either an Agilent DD2 500 MHz (11.7 T) system, at 500 and 470 MHz frequencies respectively, or on an automated Agilent DD MR 400 MHz (9.40 T) system equipped with Agilent 7600 96-sample autosampler, at 400 and 376 MHz frequencies respectively. Variable-temperature ^{1}H and ^{19}F NMR spectra of compounds **1a**, **1b**, **2a**, **2b** and **3a**, were collected on an Agilent DD2 500 MHz (11.7 T) system at 500 and 470 MHz frequencies respectively. A temperature calibration curve for the NMR spectrometer was used to convert the set temperatures on the thermostat to the actual temperatures of the measurements. NMR spectra of samples in 2.1 mM aqueous solutions of trifluoroethanol (TFE), and in 2.1 mM fetal bovine serum (FBS) solutions of NaF, were acquired using gradient autoshimming on the water proton resonance. Chemical shift values (δ) are reported in ppm and referenced to residual proton signals from the deuterated solvents for all ^{1}H NMR spectra (7.26 ppm for $CDCl_3$, 4.79 ppm for D_2O/H_2O, and 1.94 ppm for CD_3CN). ^{19}F NMR chemical shift values for spectra recorded in $CDCl_3$ and CD_3CN solvents were referenced to trichlorofluoromethane ($CFCl_3$) at 0 ppm. For spectra of compounds **1a**, **1b**, **2a**, **2b**, and **3a** in CD_3CN, the ^{19}F NMR chemical shift of the BF_4^- counterions was set to −151.44 ppm at all temperatures to make the comparison between compounds consistent. TFE was used as an internal standard for variable-temperature ^{19}F NMR measurements of compounds **1a**, **1b**, **2a**, **2b**, and **3a**, in aqueous solutions. The temperature dependence of the ^{19}F NMR chemical shift of TFE in a 2.1 mM water solution was determined relative to $CFCl_3$, and the resulting chemical shift values were used as reference points for the ^{19}F NMR chemical shifts of compounds **1a**, **1b**, **2a**, **2b**, and **3a**, at each measured temperature (4–61 °C). Variable-temperature ^{19}F NMR spectra of compounds **1a** and **2a** in FBS were collected under the same conditions as ^{19}F NMR spectra of these compounds in aqueous TFE solutions, except NaF (2.1 mM) was used as an internal standard because of potential reactivity of TFE with serum albumin. The ^{19}F NMR chemical shift of NaF at each recorded temperature was determined with respect to TFE in a separate experiment, conducted in an aqueous solution containing 1.1 mM TFE and 5.2 mM NaF. All coupling constants (J) were measured in Hertz (Hz). The MestReNova 10.0 NMR data processing software was used to analyze and process all recorded NMR spectra.

Magnetic measurements. The magnetic moments of compounds **1a** and **2a** in solutions, were determined using the Evans method[8] by collecting variable-temperature ^{1}H NMR spectra using a Varian Inova 400 MHz (9.40 T) spectrometer. For aqueous solution measurements, the compounds **1a** and **2a** were dissolved in a 2.1 mM solution of TFE in H_2O and dimethyl sulfoxide (DMSO) was added as a reference (2 v/v % of DMSO). The resulting solution was placed in a NMR tube containing a sealed capillary with a solution of H_2O and DMSO (2 v/v % of DMSO). For acetonitrile solution measurements, compound **1a** was dissolved in a mixture of CD_3CN and CH_2Cl_2 (2 v/v % of CH_2Cl_2), and the resulting solution was placed in a NMR tube containing a sealed capillary with a solution of CD_3CN and CH_2Cl_2 (2 v/v % of CH_2Cl_2). The average of three measurements afforded the resulting data. All data were corrected for diamagnetic contributions from the core diamagnetism of each sample (estimated using Pascal's constants).[9] The molar fraction of spin-crossover compound **1a** in high-spin state as a function of temperature was estimated by equation 1:

$$\gamma_{HS} = (\chi T_{obs} - \chi T_{LS})/(\chi T_{HS} - \chi T_{LS}) \approx \chi T_{obs} / \chi T_{HS} \quad (1)$$

χT for the fully populated $S = 0$ (low-spin) ground state (χT_{LS}) was approximated to be equal to 0 cm^3 K mol^{-1}. An average value of χT for the high-spin compound **2a** (3.63 cm^3 K mol^{-1}) in an aqueous solution, over the temperature range studied (278–333 K) was used as the fully populated $S = 2$ (high-spin) excited state limit (χT_{HS}). The thermodynamic parameters, ΔH and ΔS, associated with the spin transition in solution, as measured by the Evans method, were obtained using the regular solution model:[10]

$$R \cdot \ln(\gamma_{HS} / (1 - \gamma_{HS})) = -\Delta H/T + \Delta S \quad (2)$$

UV/Visible absorption measurements. UV/Visible experiments were carried out on an Agilent Cary 5000 UV/Vis/NIR spectrometer. UV/Visible spectra were collected in the 200–800 nm range in MeCN and H_2O solvents using 9–75 μM solutions of compounds **1a**, **1b**, **2a**, **2b** and **3a**.

Electrospray ionization mass spectrometry (ESI-MS) measurements. ESI-MS spectra were recorded on a LC-MS Bruker Amazon SL quadrupole ion trap instrument, equipped with a Compass Software 1.3 SR2. All measurements were carried out in a CH_2Cl_2/MeOH (4:1) carrier solvent using positive ionization mode.

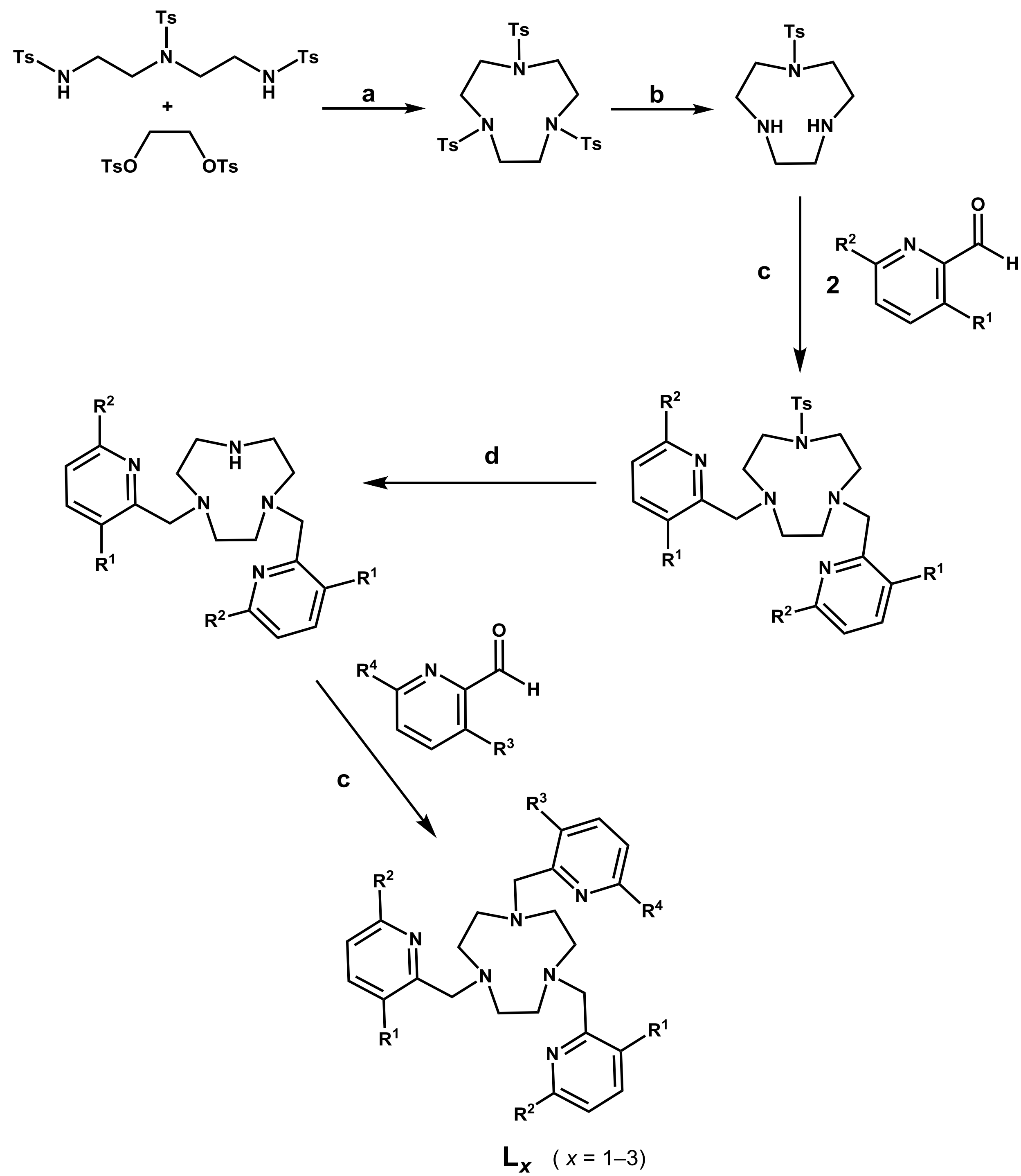

L*ₓ* (*x* = 1–3)

L₁: R¹ = F, R²/R³ = H, R⁴ = Me
L₂: R¹/R³ = F, R²/R⁴ = Me
L₃: R¹/R³ = F, R²/R⁴ = H

Scheme S1 | Synthesis of ligands L_x (x = 1–3). Reagents and solvents: (a) Cs_2CO_3, DMF; (b) HBr/HOAc; (c) $Na(OAc)_3BH$, CH_2Cl_2; (d) conc. H_2SO_4.

Table S1 | Crystallographic data for compounds **1a**·$0.5CH_3CN$, **1b**, **2a**, **2b**, and **3a**.

	**1a**·$0.5CH_3CN$	**1b**	**2a**	**2b**	**3a**
Empirical formula	$C_{26}H_{31.5}B_2F_{10}FeN_{6.5}$	$C_{25}H_{30}B_2F_{10}N_6Zn$	$C_{27}H_{33}B_2F_{11}FeN_6$	$C_{27}H_{33}B_2F_{11}N_6Zn$	$C_{24}H_{27}B_2F_{11}FeN_6$
Formula weight, g mol^{-1}	702.55	691.54	728.06	737.58	685.98
Crystal system	Triclinic	Monoclinic	Cubic	Cubic	Trigonal
Space group	$P-1$	Pc	$F-43c$	$F-43c$	$P3$
Wavelength, Å	0.71073	0.71073	0.71073	0.71073	0.71073
Temperature, K	100	100	100	100	100
a, Å	11.4603(7)	13.6092(4)	29.3374(7)	29.2820(8)	16.8376(5)
b, Å	14.9231(8)	14.5112(4)	29.3374(7)	29.2820(8)	16.8376(5)
c, Å	18.6992(11)	14.1222(4)	29.3374(7)	29.2820(8)	7.8829(3)
α, °	110.254(3)	90	90	90	90
β, °	106.434(3)	90.875(1)	90	90	90
γ, °	91.133(3)	90	90	90	120
V, $Å^3$	2853.0(3)	2788.61(14)	25250.2(18)	25107(2)	1935.43(14)
Z	4	4	32	32	3
ρ_{calcd}, g cm^{-3}	1.636	1.647	1.532	1.561	1.766
μ, mm^{-1}	0.626	0.977	0.573	0.878	0.694
Reflections coll./unique	126647/16997	80246/16461	448528/5143	274422/4026	115769/12595
R(int)	0.0360	0.0355	0.0574	0.0396	0.0390
$R_1\ (I > 2\sigma(I))^a$	0.0448	0.0429	0.0486	0.0355	0.0365
$wR_2\ (all)^b$	0.1153	0.1088	0.1548	0.1030	0.0958
GoF	1.059	1.025	1.049	1.073	1.026

^{a} $R_1 = \Sigma||F_0| - |F_C||/\Sigma|F_0|$, ^{b} $wR_2 = [\Sigma w(F_0^2 - F_C^2)^2/\Sigma w(F_0^2)^2]^{1/2}$.

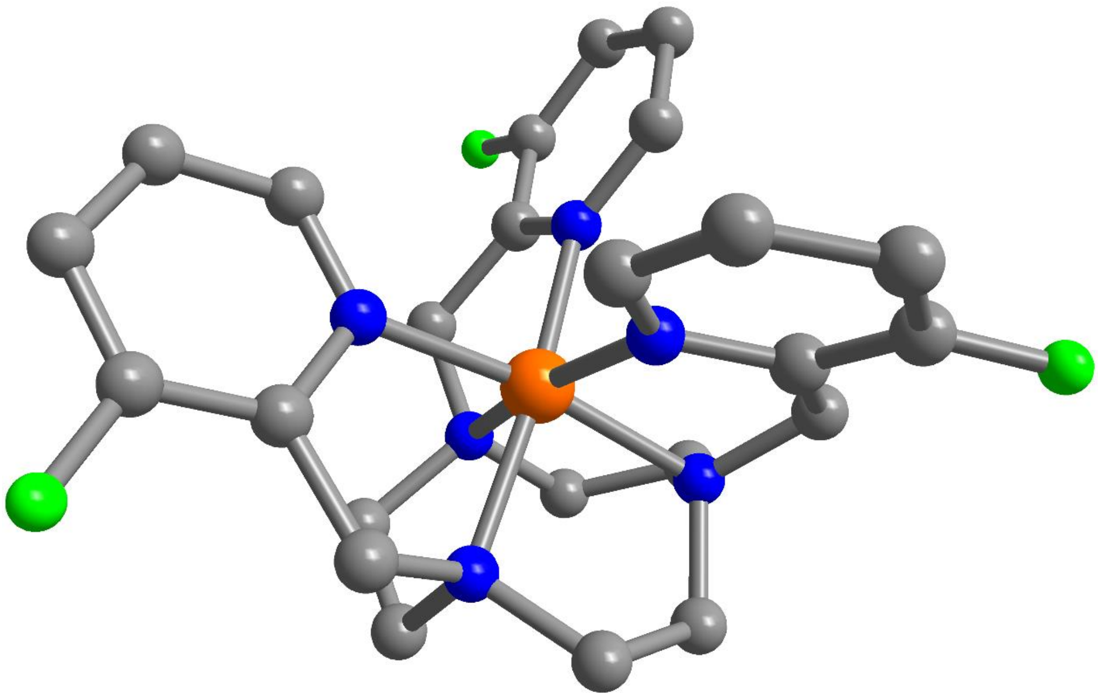

Figure S1 | Crystal structure of $[Fe(L_3)]^{2+}$, as observed in **3a**. Orange, green, blue and gray spheres represent Fe, F, N and C atoms, respectively; H atoms are omitted for clarity.

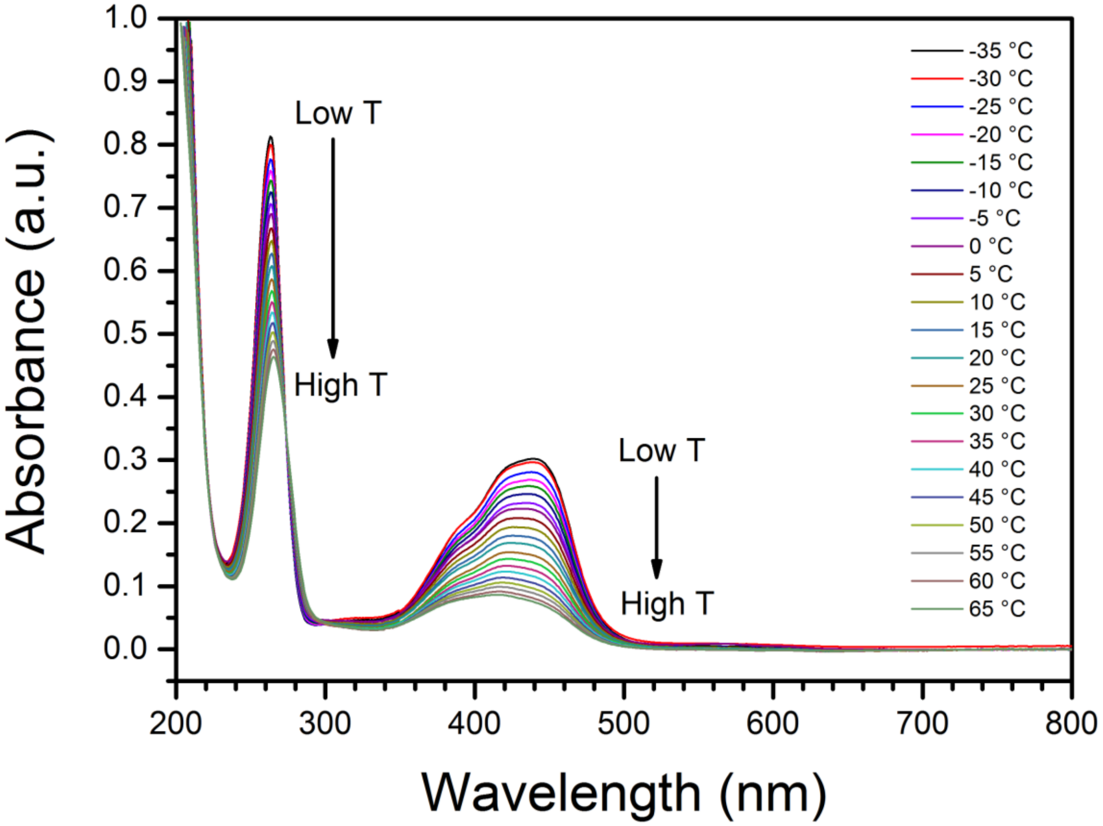

Figure S2 | Variable-temperature UV/Visible spectra of a 55 μM solution of **1a** in dry MeCN. Spectra were measured in the temperature range −35–65 °C with 5 °C increments. The temperature of each recorded spectrum is given with the color assignment in the legend.

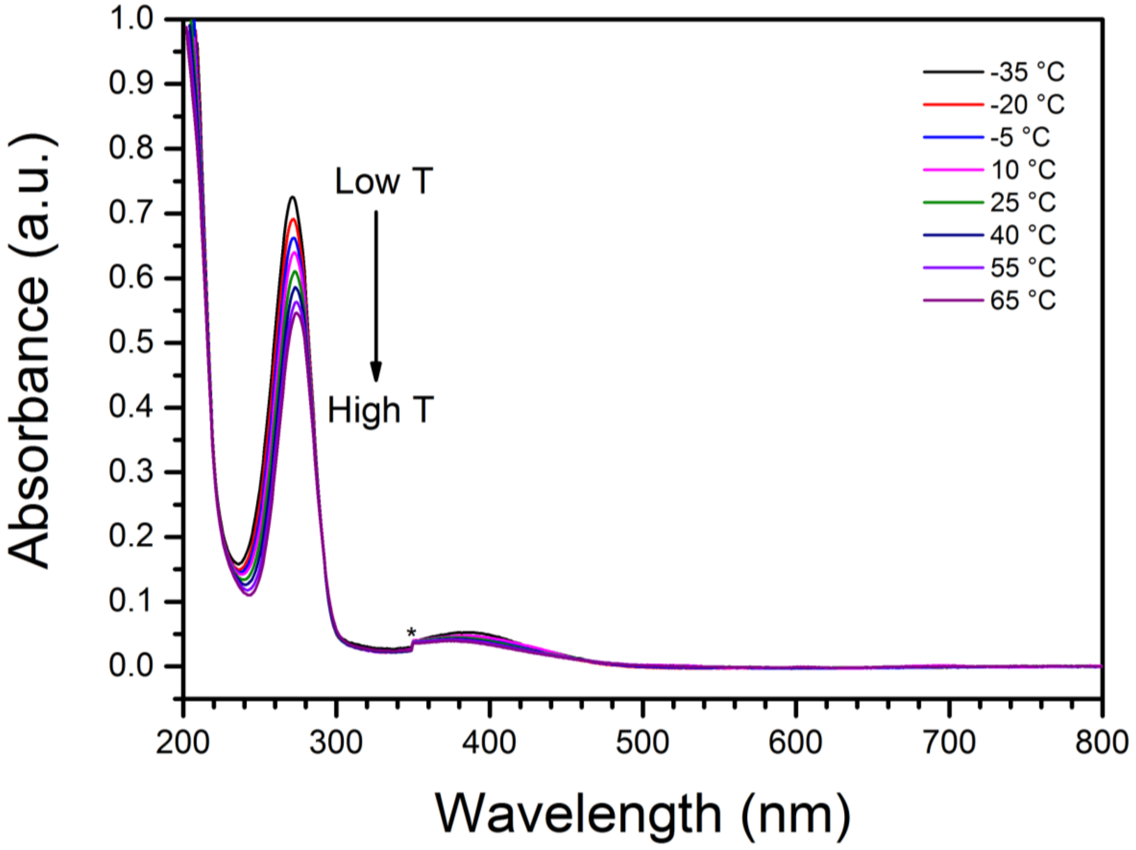

Figure S3 | Variable-temperature UV/Visible spectra of a 55 μM solution of **2a** in dry MeCN. Spectra were measured in the temperature range −35–65 °C with either 10 or 15 °C increments. The temperature of each recorded spectrum is given with the color assignment in the legend. The asterisk denotes an instrument derived artifact.

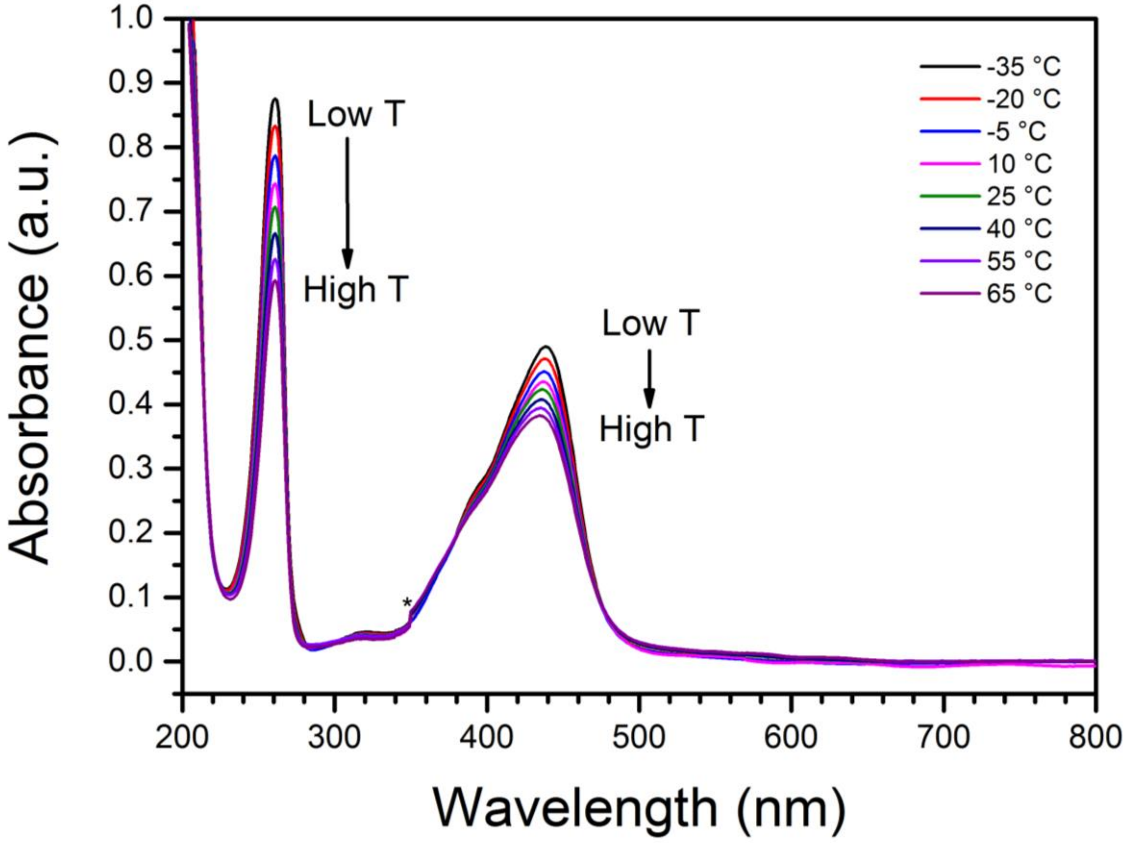

Figure S4 | Variable-temperature UV/Visible spectra of a 40 μM solution of **3a** in dry MeCN. Spectra were measured in the temperature range −35–65 °C with either 10 or 15 °C increments. The temperature of each recorded spectrum is given with the color assignment in the legend. The asterisk denotes an instrument derived artifact.

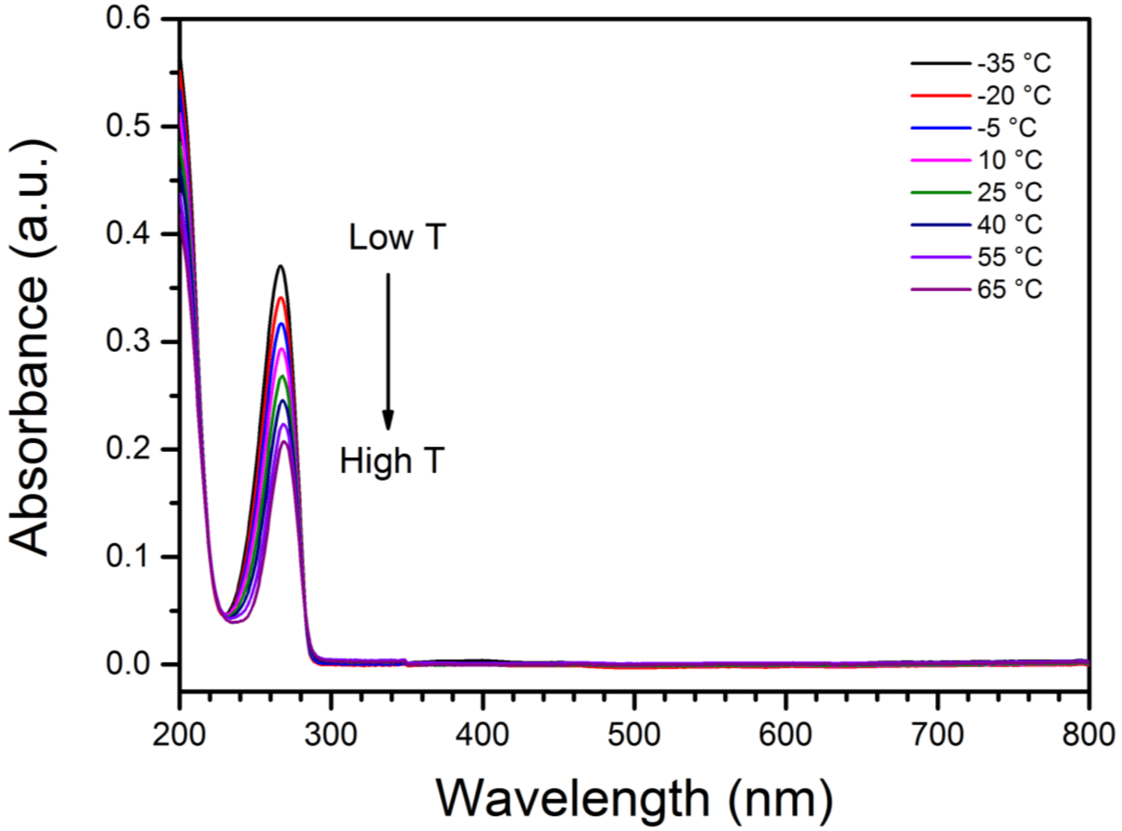

Figure S5 | Variable-temperature UV/Visible spectra of an 8.8 μM solution of **1b** in dry MeCN. Spectra were measured in the temperature range −35–65 °C with either 10 or 15 °C increments. The temperature of each recorded spectrum is given with the color assignment in the legend.

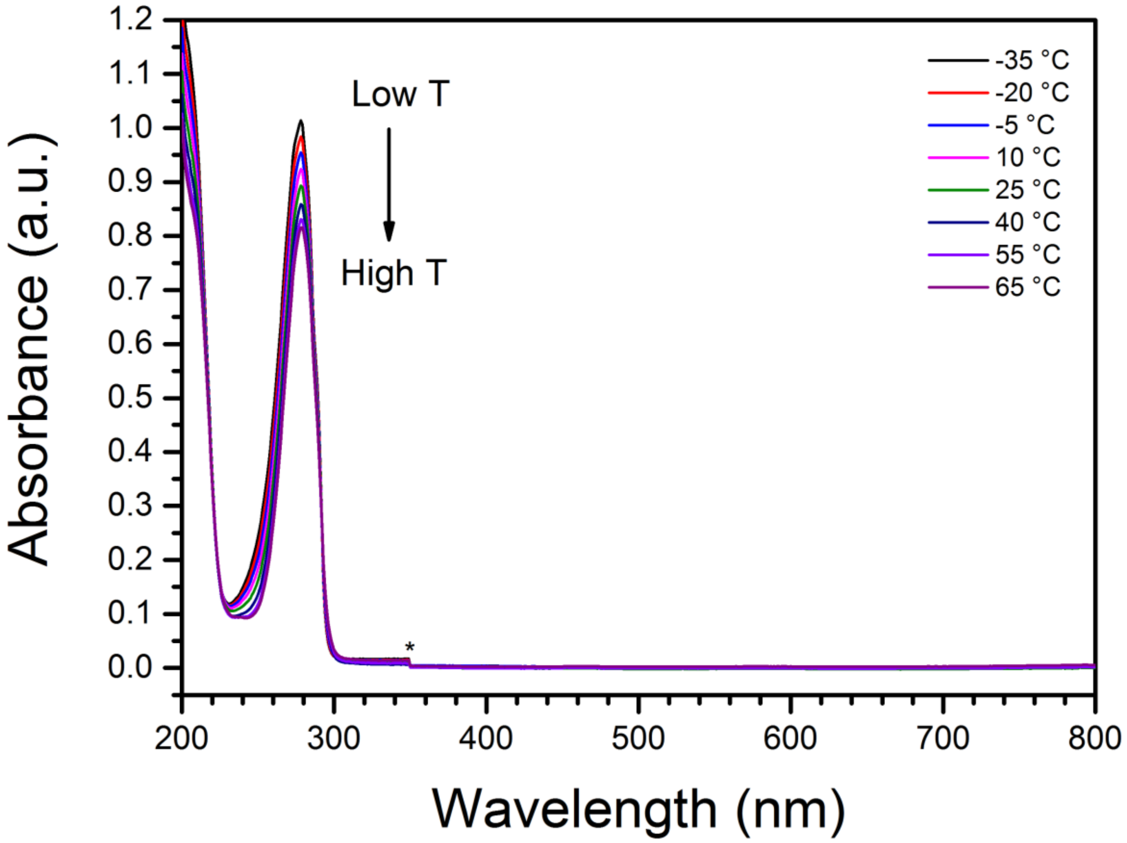

Figure S6 | Variable-temperature UV/Visible spectra of a 25 μM solution of **2b** in dry MeCN. Spectra were measured in the temperature range −35–65 °C with either 10 or 15 °C increments. The temperature of each recorded spectrum is given with the color assignment in the legend. The asterisk denotes an instrument derived artifact.

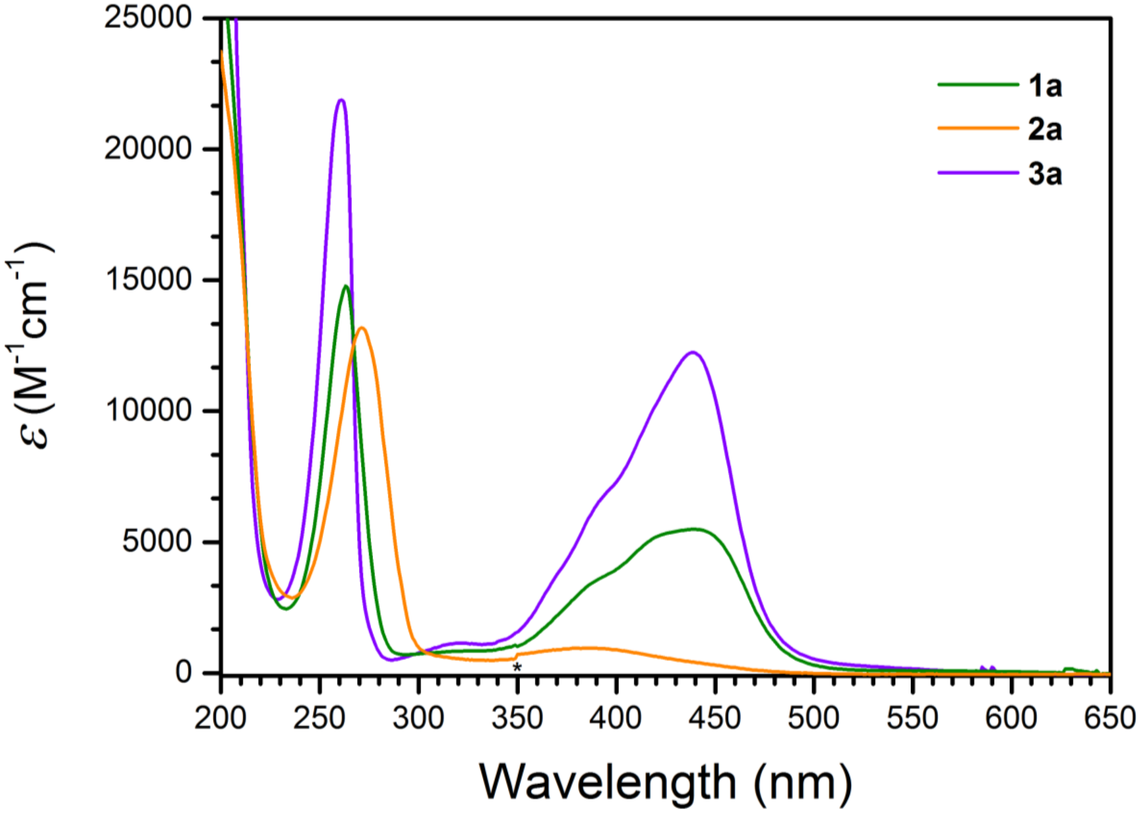

Figure S7 | Comparison of UV/Visible spectra of compounds **1a**, **2a** and **3a** in dry MeCN at −35 °C. Note that the molar absorption coefficient (ε), instead of absorbance, is plotted as a function of wavelength to correct for the different concentrations of samples of **1a**, **2a** and **3a**. The asterisk denotes an instrument derived artifact.

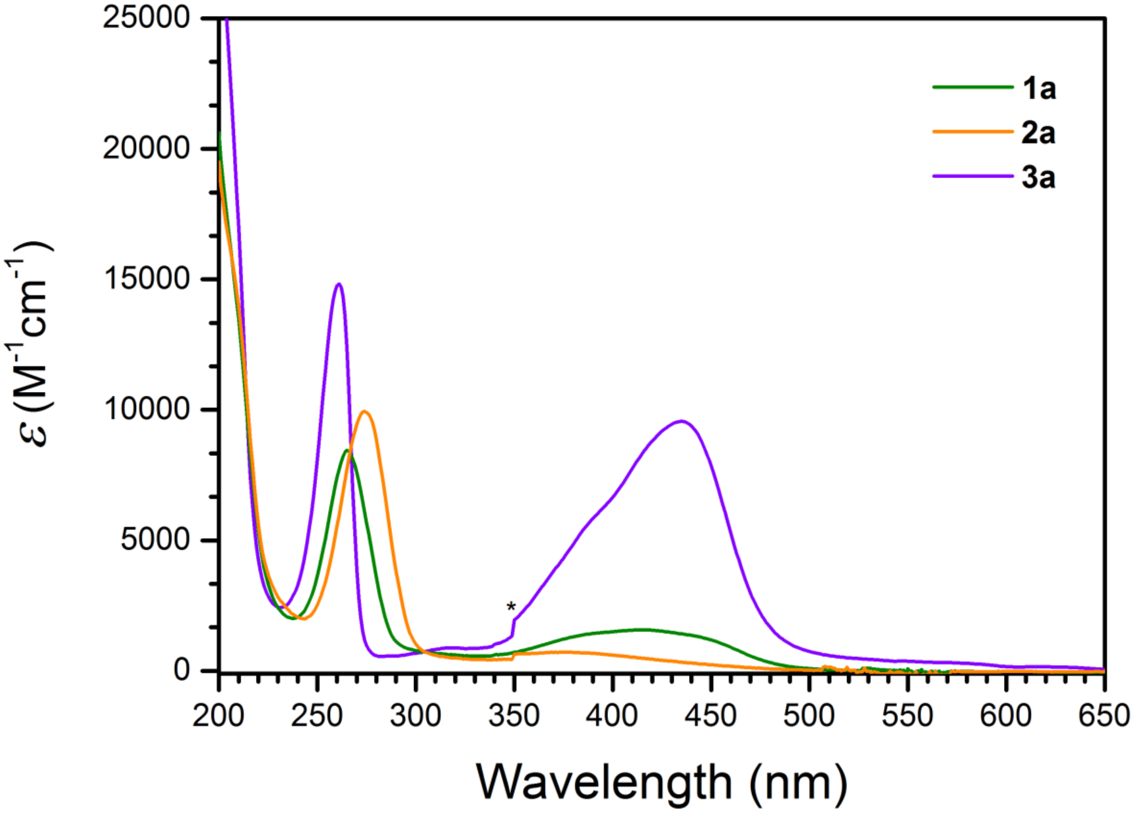

Figure S8 | Comparison of UV/Visible spectra of compounds **1a**, **2a** and **3a** in dry MeCN at 65 °C. Note that the molar absorption coefficient (ε), instead of absorbance, is plotted as a function of wavelength to correct for the different concentrations of samples of **1a**, **2a** and **3a**. The asterisk denotes an instrument derived artifact.

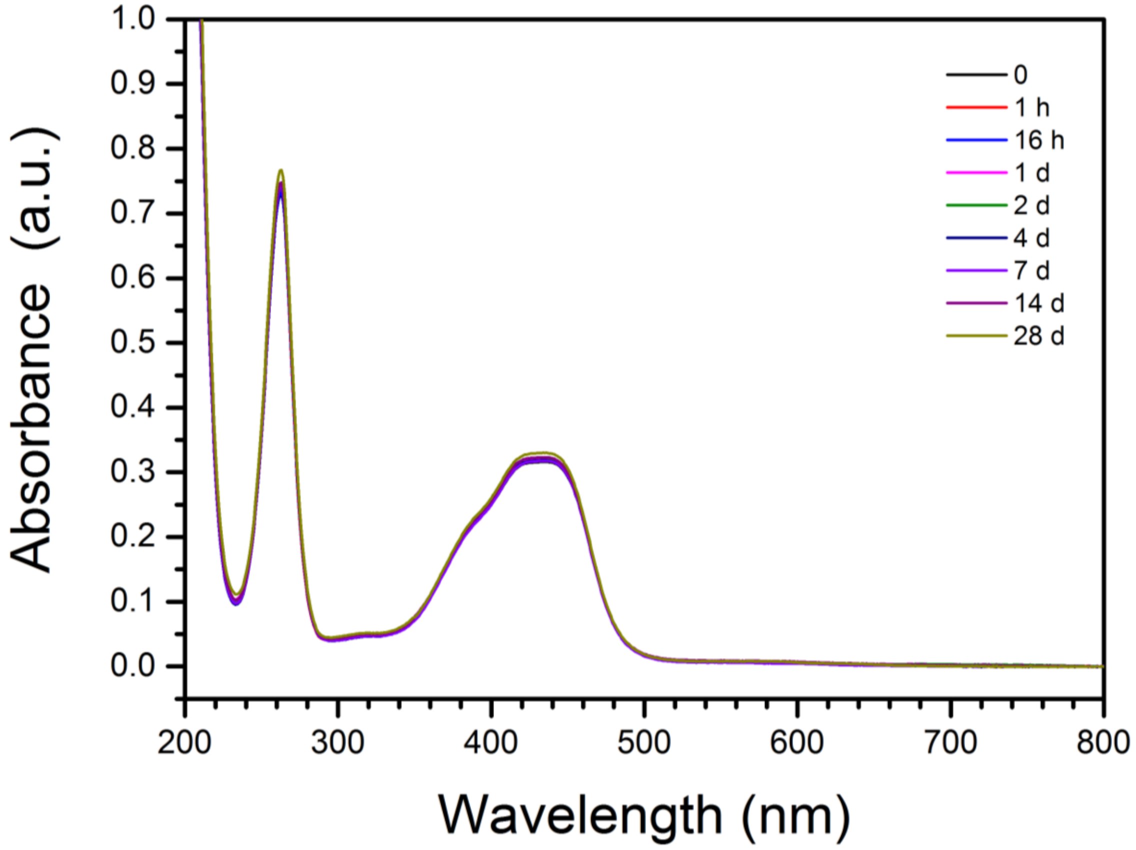

Figure S9 | UV/Visible spectra of a 59 μM solution of **1a** in deoxygenated water and under aerobic conditions, respectively, at ambient temperature. The color assignment in the legend indicates the time that the sample solution was open to air prior to the measurement was performed.

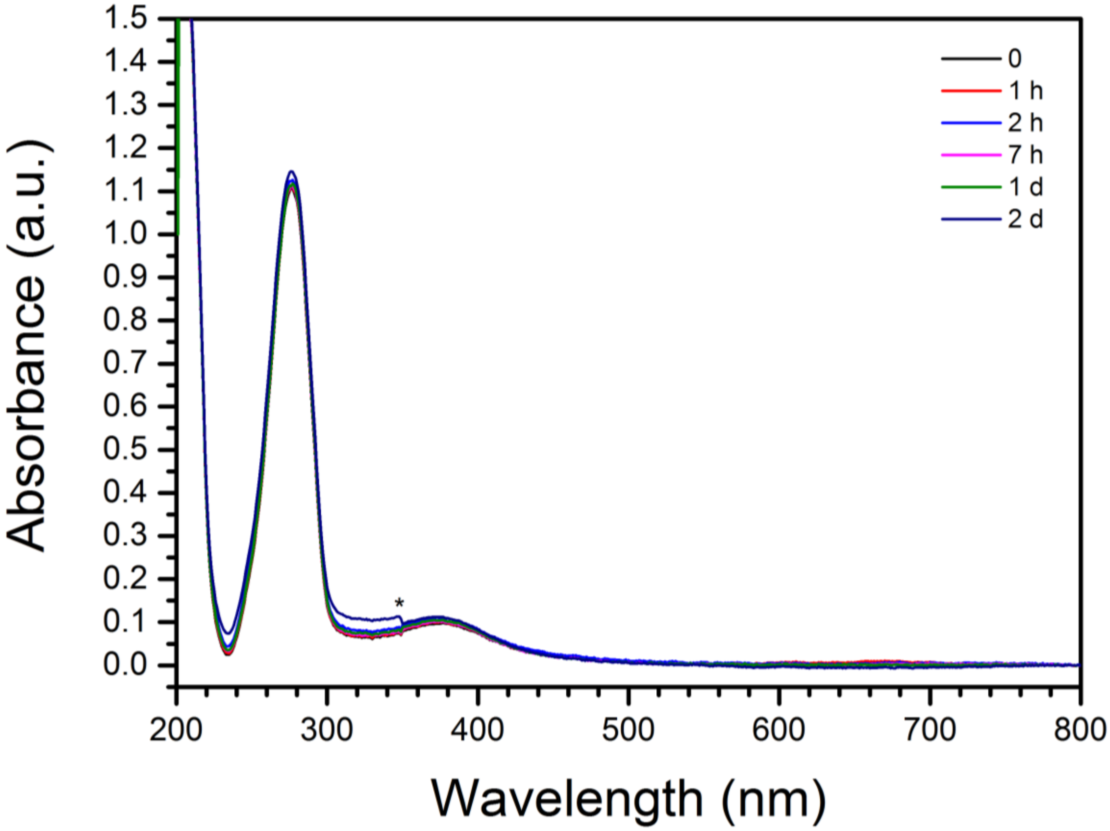

Figure S10 | UV/Visible spectra of a 69 μM solution of **2a** in deoxygenated water and under aerobic conditions, respectively, at ambient temperature. The color assignment in the legend indicates the time that the sample solution was open to air prior to the measurement was performed. The asterisk denotes an instrument derived artifact.

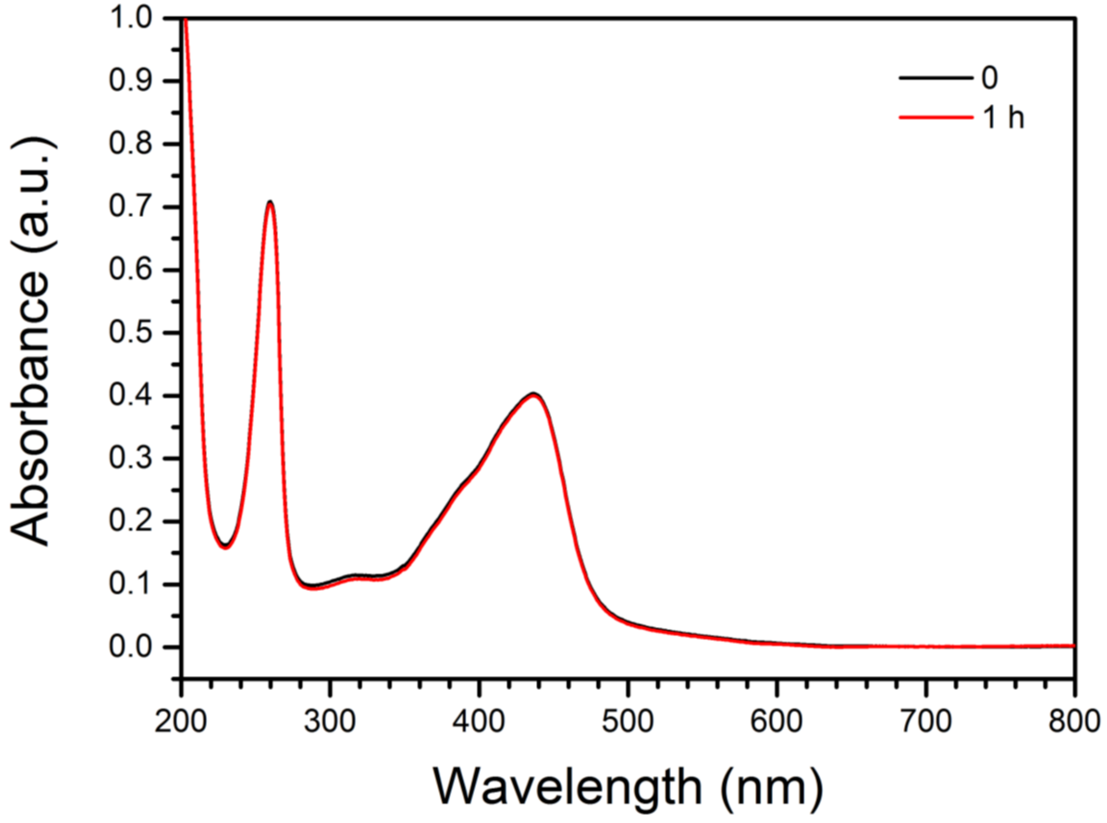

Figure S11 | UV/Visible spectra of a 51 μM solution of **3a** in deoxygenated water (black curve) and under aerobic conditions (red curve) at ambient temperature. The color assignment in the legend indicates the time that the sample solution was open to air prior to the measurement was performed.

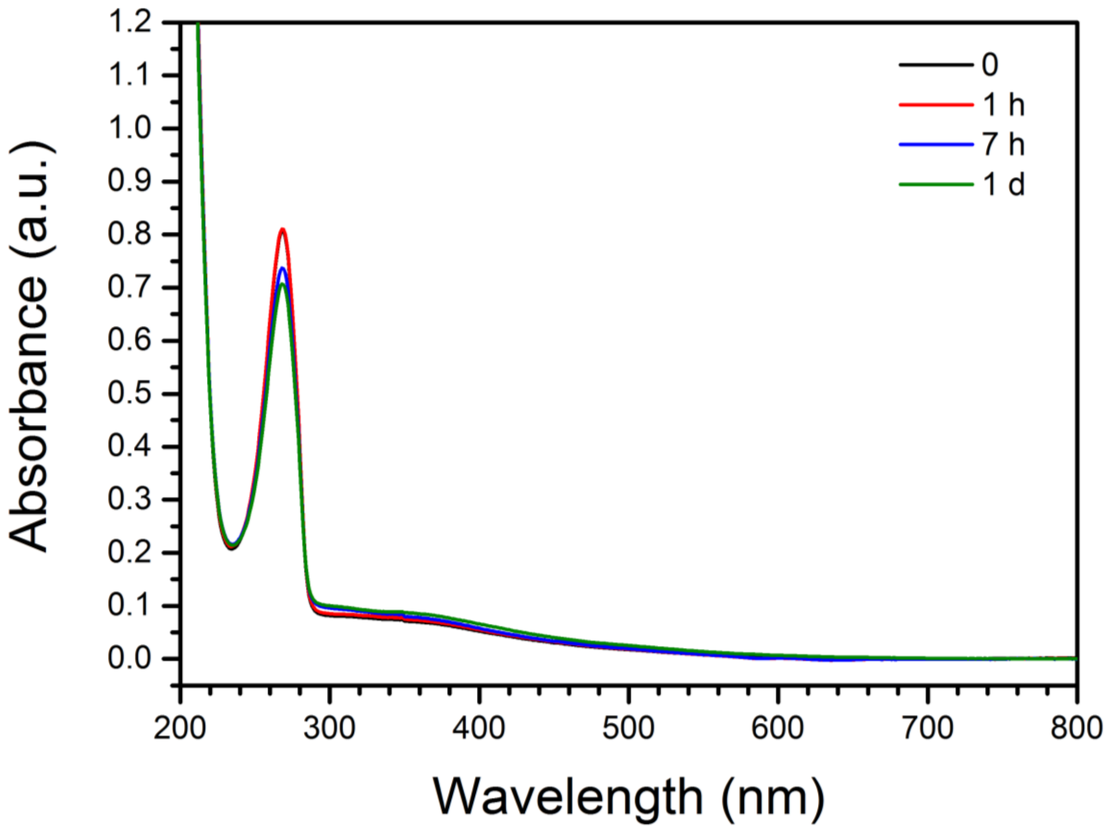

Figure S12 | UV/Visible spectra of a 72 μM solution of **1b** in deoxygenated water and under aerobic conditions, respectively, at ambient temperature. The color assignment in the legend indicates the time that the sample solution was open to air prior to the measurement was performed. Note that the slight decrease in intensity observed with time in air is attributed to a dilution of the sample solution that took place when the sample was moved back and forth between the cuvette and a sample container.

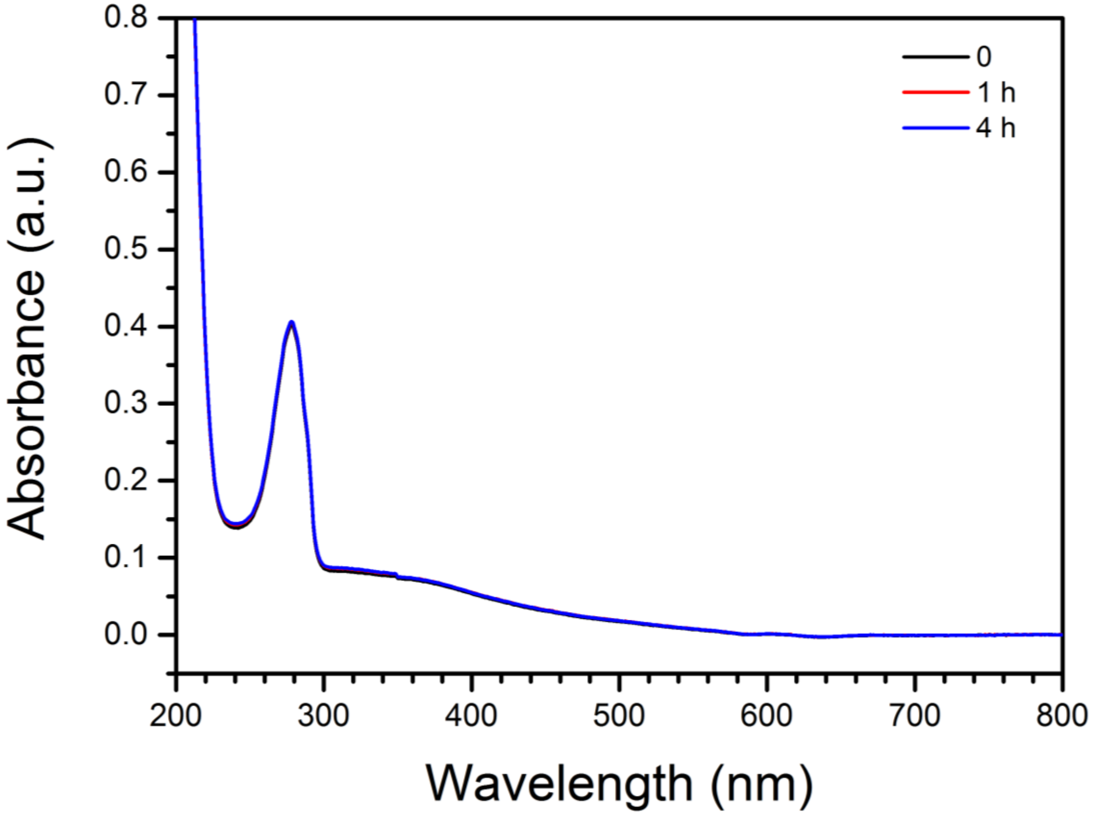

Figure S13 | UV/Visible spectra of a 30 μM solution of **2b** in deoxygenated water and under aerobic conditions, respectively, at ambient temperature. The color assignment in the legend indicates the time that the sample solution was open to air prior to the measurement was performed.

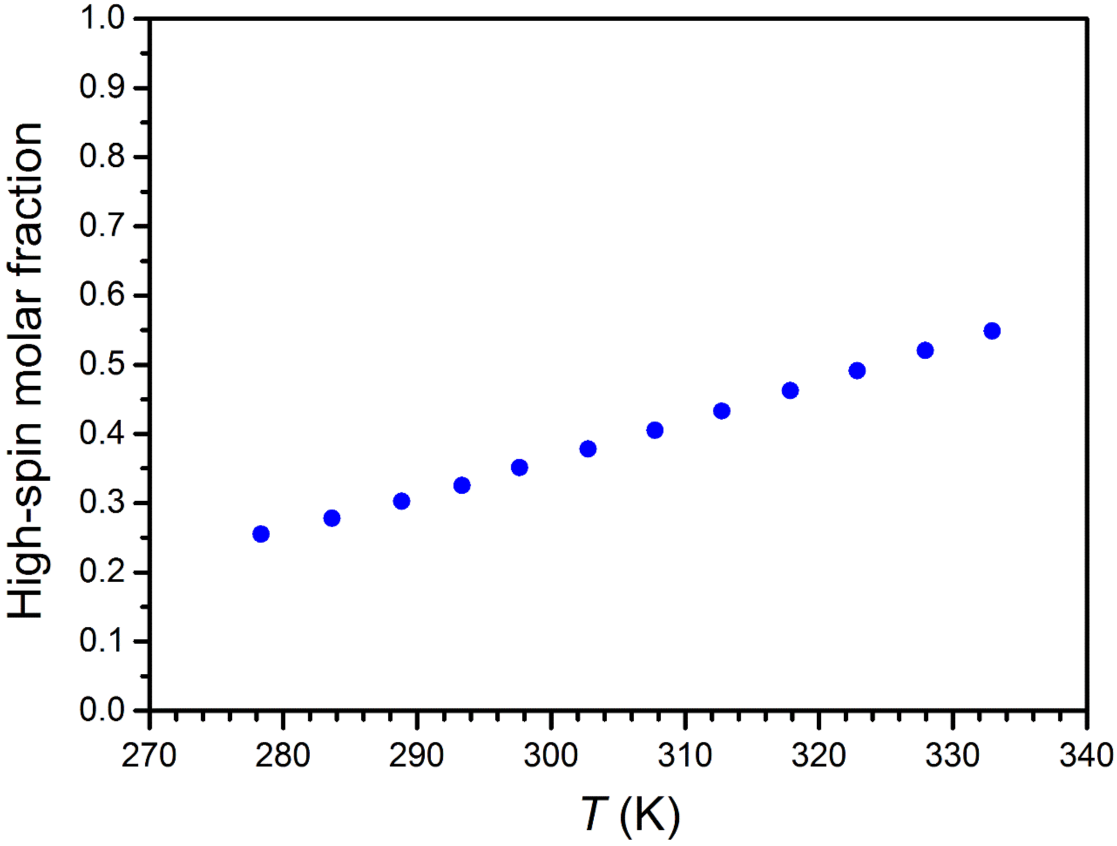

Figure S14 | Plot of the molar fraction of **1a** in high-spin state as a function of temperature, obtained using equation 1. A linear fit to the data gives a spin-crossover temperature ($T_{1/2}$) of 325(1) K.

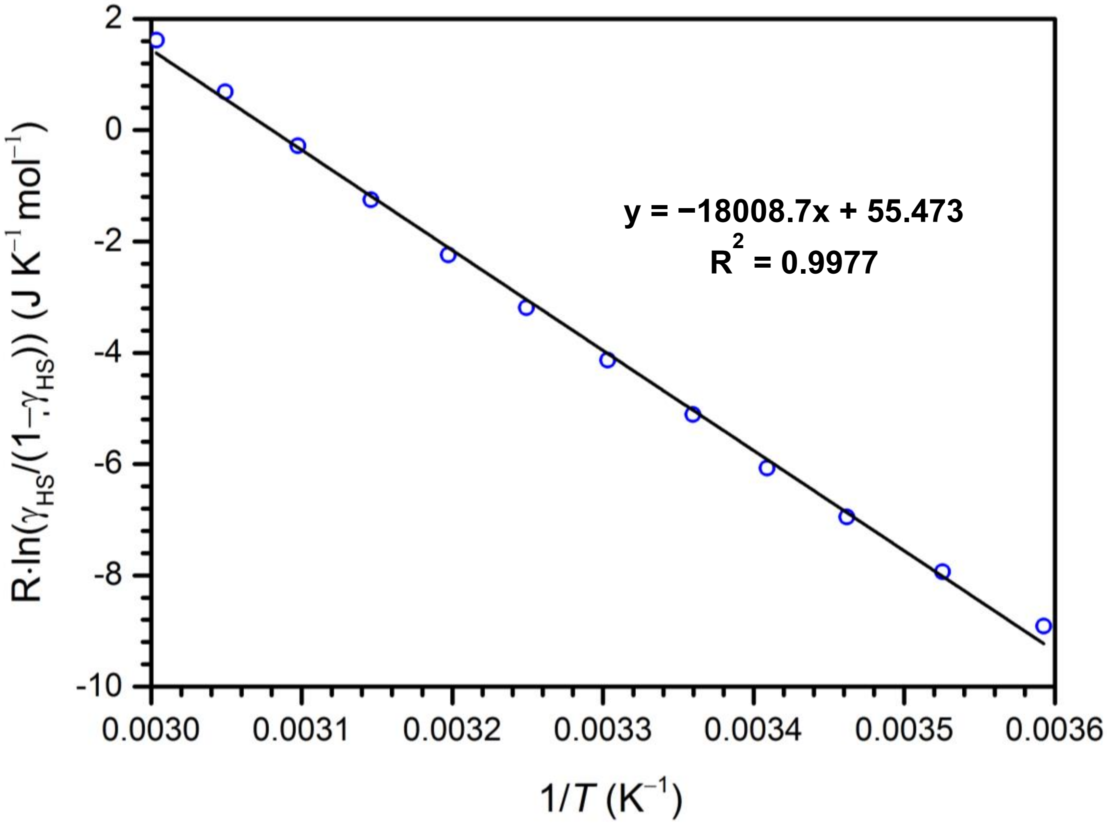

Figure S15 | Thermodynamic data for the spin transition of **1a** in an aqueous solution, as measured by the Evans method, obtained using the regular solution model (equation 2). The solid black line indicates a linear fit to the data, giving the following thermodynamic parameters: ΔH = 18.0(3) kJ mol^{-1} and ΔS = 55.5(9) J K^{-1} mol^{-1}.

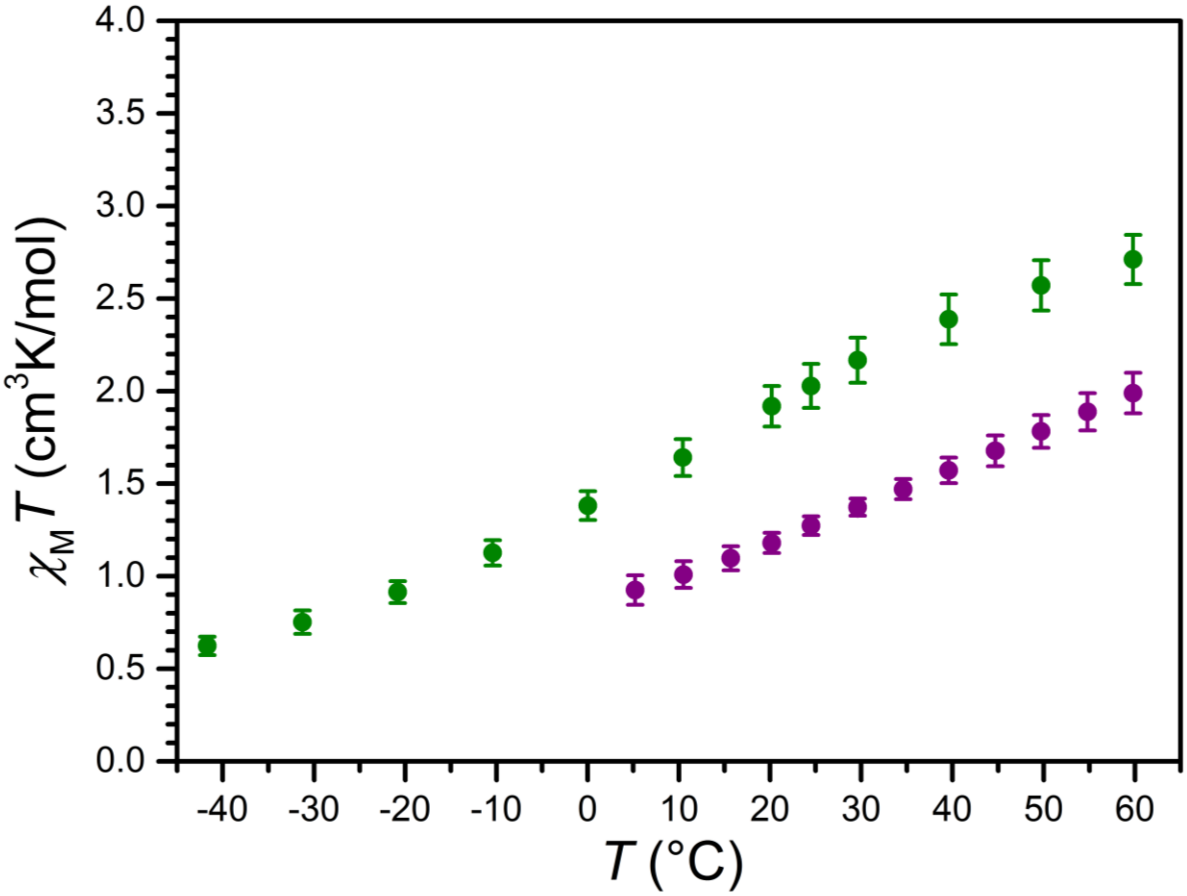

Figure S16 | Variable-temperature solution magnetic susceptibility data for **1a** in aqueous (purple) and acetonitrile (green) solutions, obtained using the Evans method. Error bars represent standard deviations of the measurements.

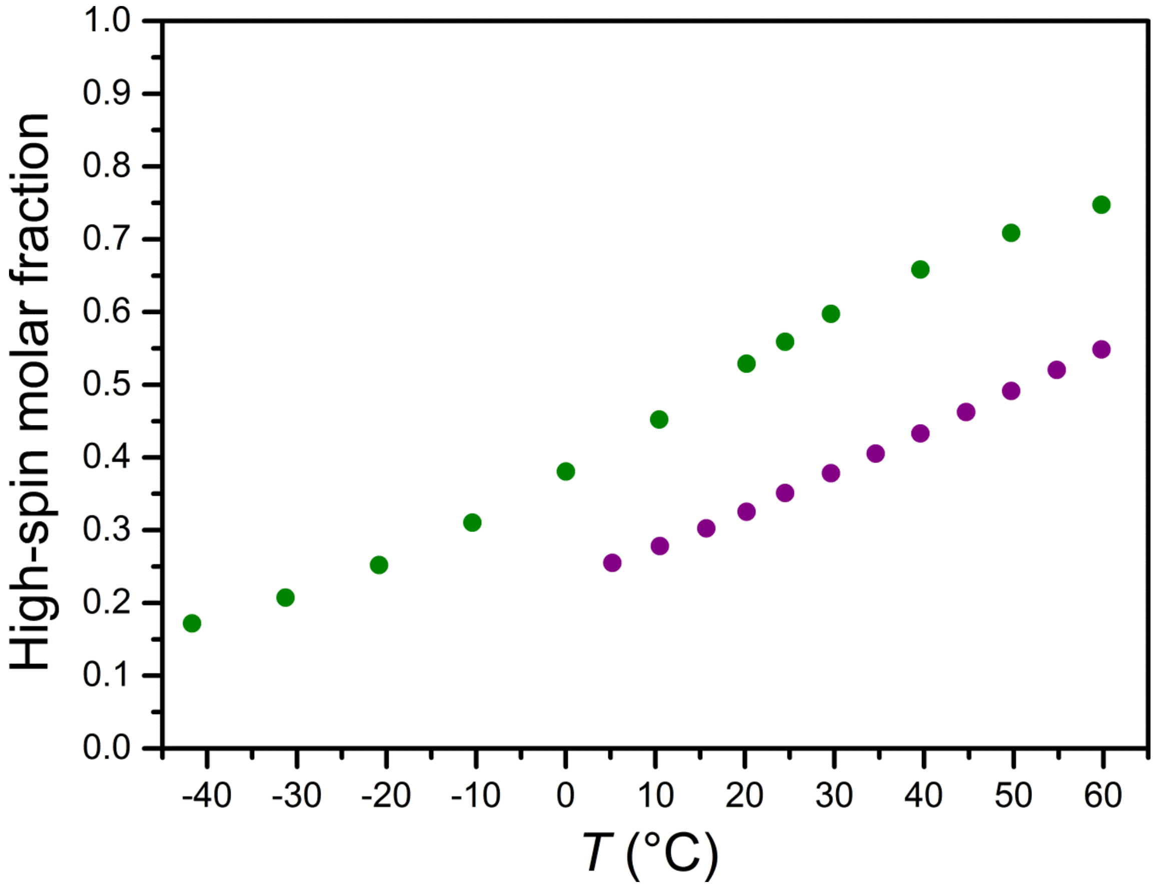

Figure S17 | Comparison of the molar fraction of **1a** in high-spin state as a function of temperature in aqueous (purple) and acetonitrile (green) solutions, obtained using equation 1. Linear fits to the data give a spin-crossover temperature ($T_{1/2}$) of 52(1) and 17(1) °C in aqueous and acetonitrile solutions, respectively.

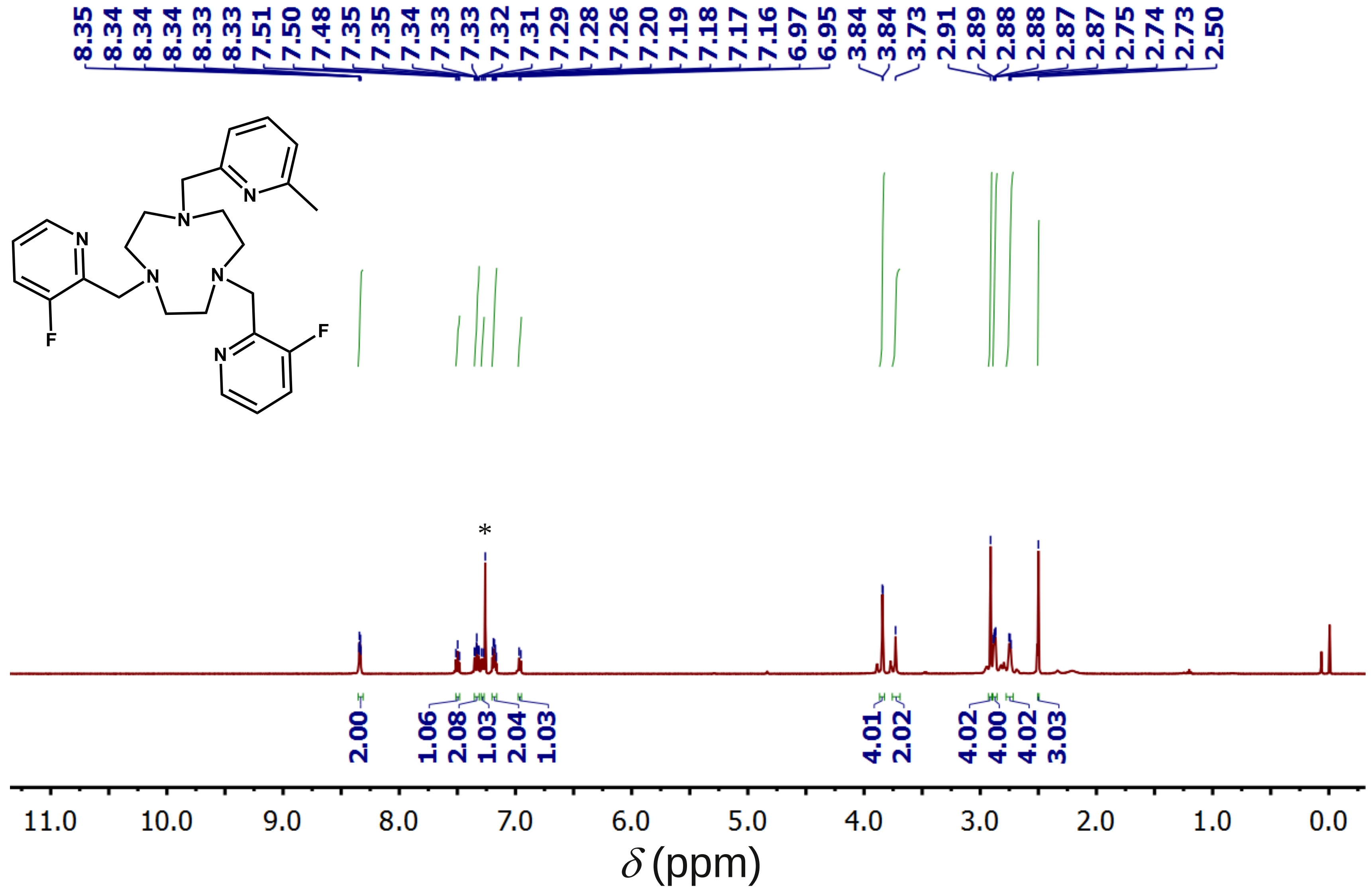

Figure S18 | ^{1}H NMR spectrum of *N*,*N′*-di(3-fluoro-2-picolyl)-*N″*-mono(6-methyl-2-picolyl)-1,4,7-triazacyclononane (L_1) in $CDCl_3$ at 25 °C. The asterisk denotes the residual $CHCl_3$ solvent peak.

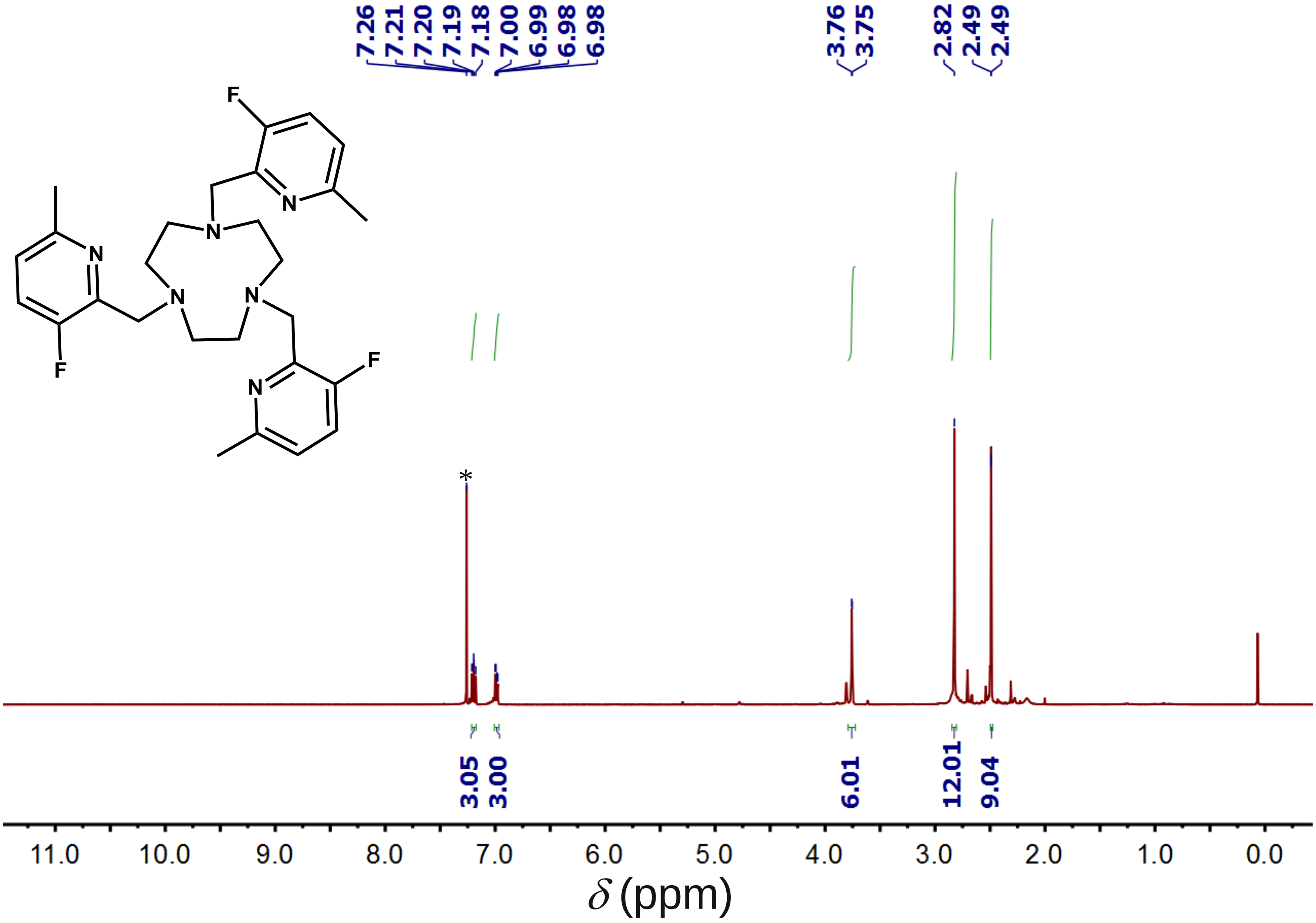

Figure S19 | ^{1}H NMR spectrum of *N,N′,N″*-tri(3-fluoro-6-methyl-2-picolyl)-1,4,7-triazacyclononane (L_2) in $CDCl_3$ at 25 °C. The asterisk denotes the residual $CHCl_3$ solvent peak.

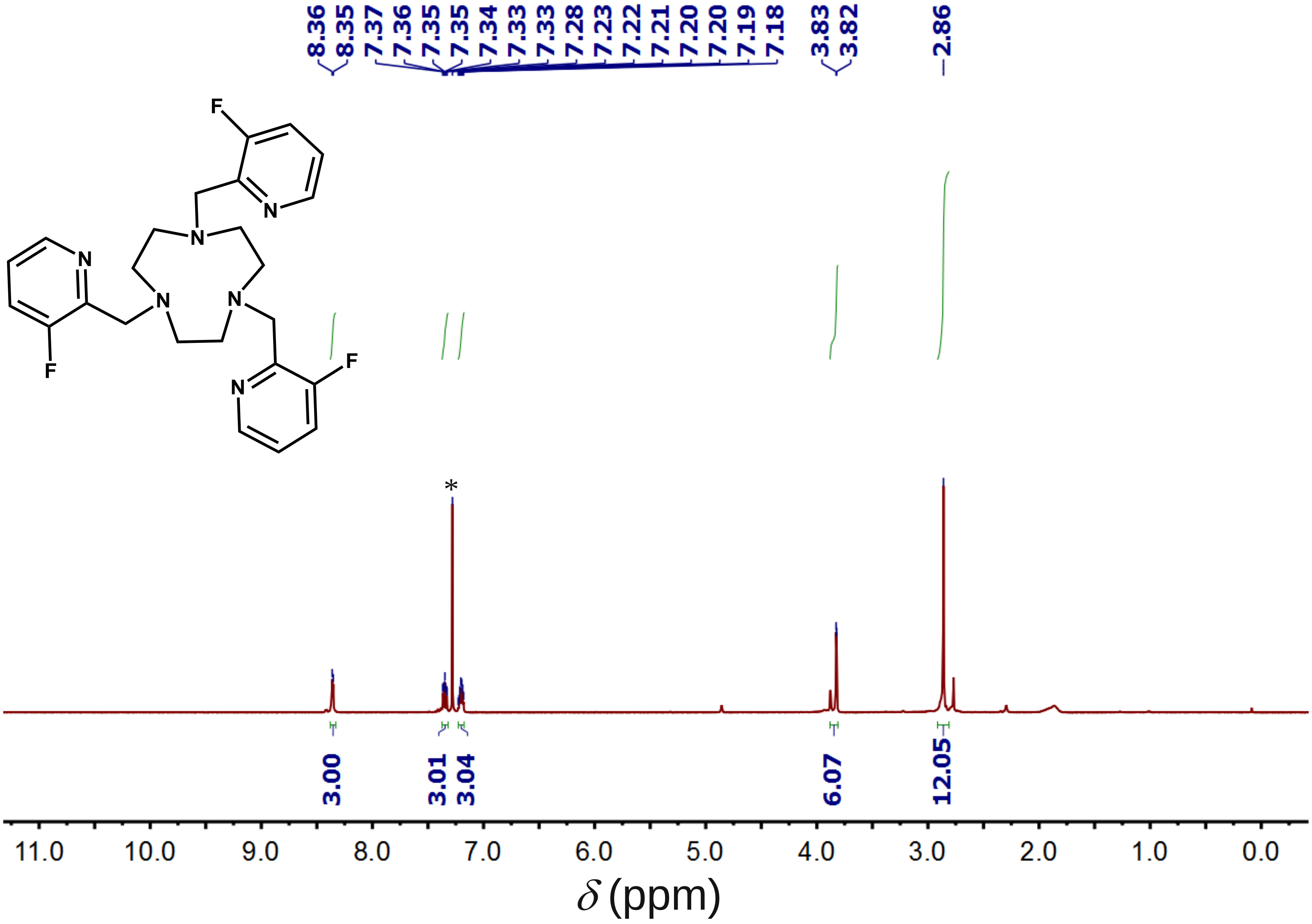

Figure S20 | ^{1}H NMR spectrum of *N,N′,N″*-tri(3-fluoro-2-picolyl)-1,4,7-triazacyclononane (L_3) in $CDCl_3$ at 25 °C. The asterisk denotes the residual $CHCl_3$ solvent peak

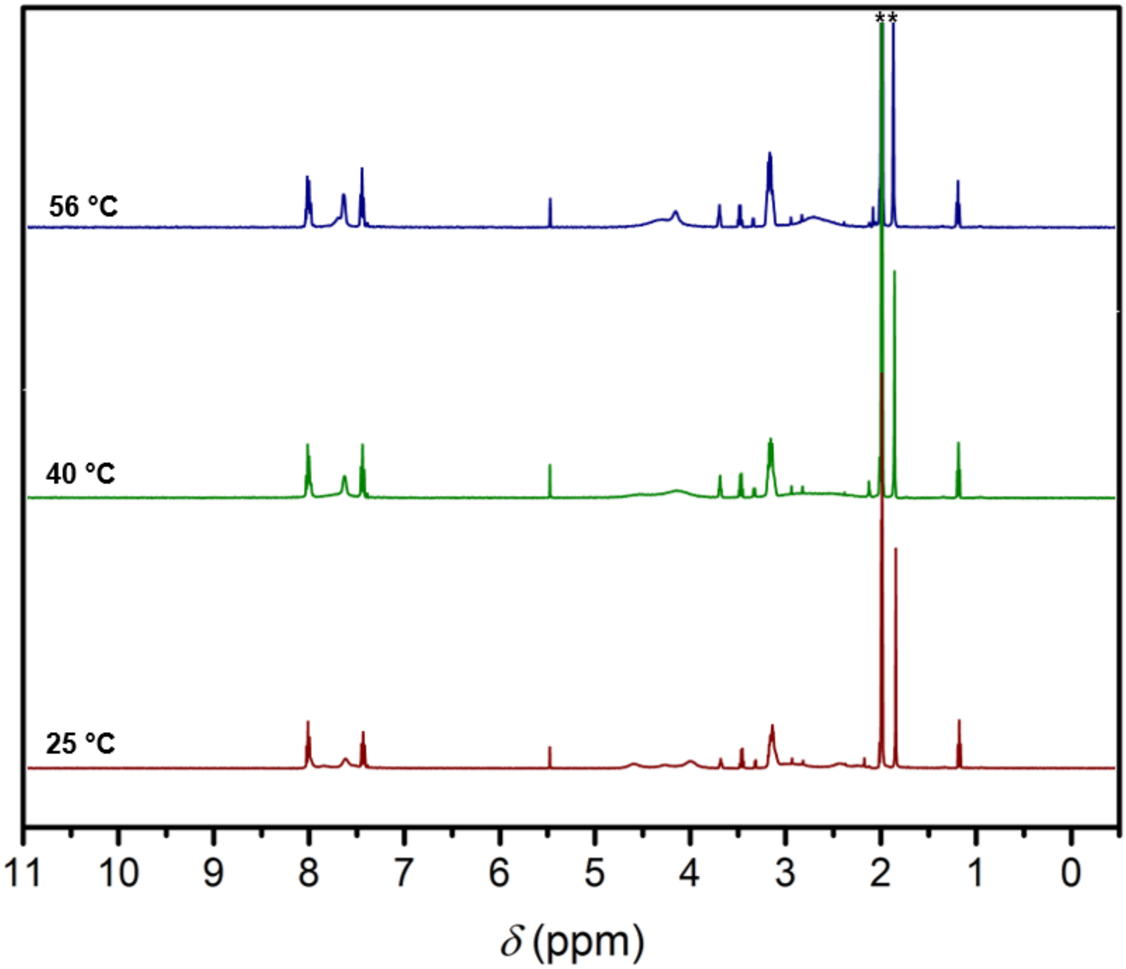

Figure S21 | Variable-temperature ^{1}H NMR spectra of **1b** in CD_3CN at 25 to 56 °C. The asterisks denote residual solvent peaks.

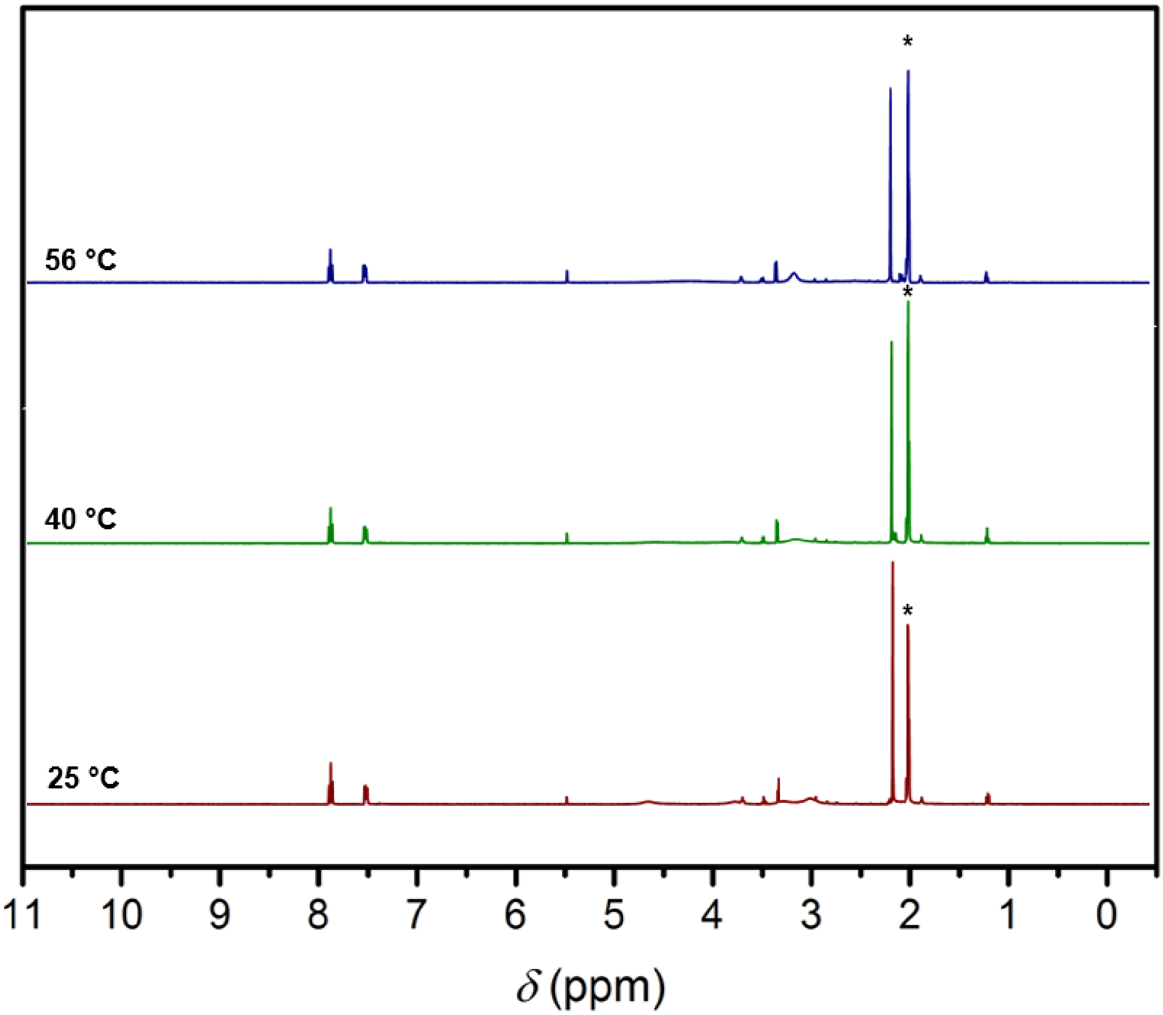

Figure S22 | Variable-temperature ^{1}H NMR spectra of **2b** in CD_3CN at 25 to 56 °C. The asterisk denotes the residual solvent peak.

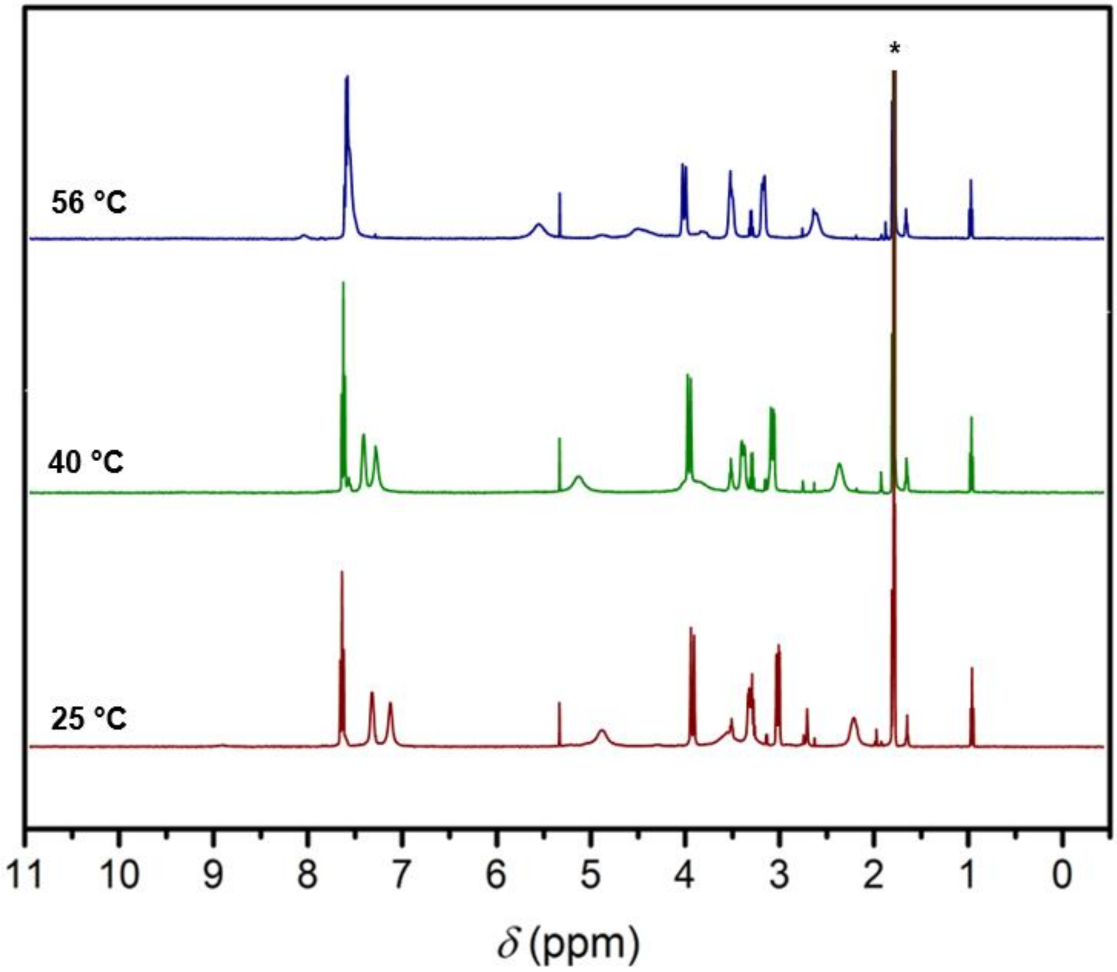

Figure S23 | Variable-temperature ^{1}H NMR spectra of **3a** in CD_3CN at 25 to 56 °C. The asterisk denotes the residual solvent peak.

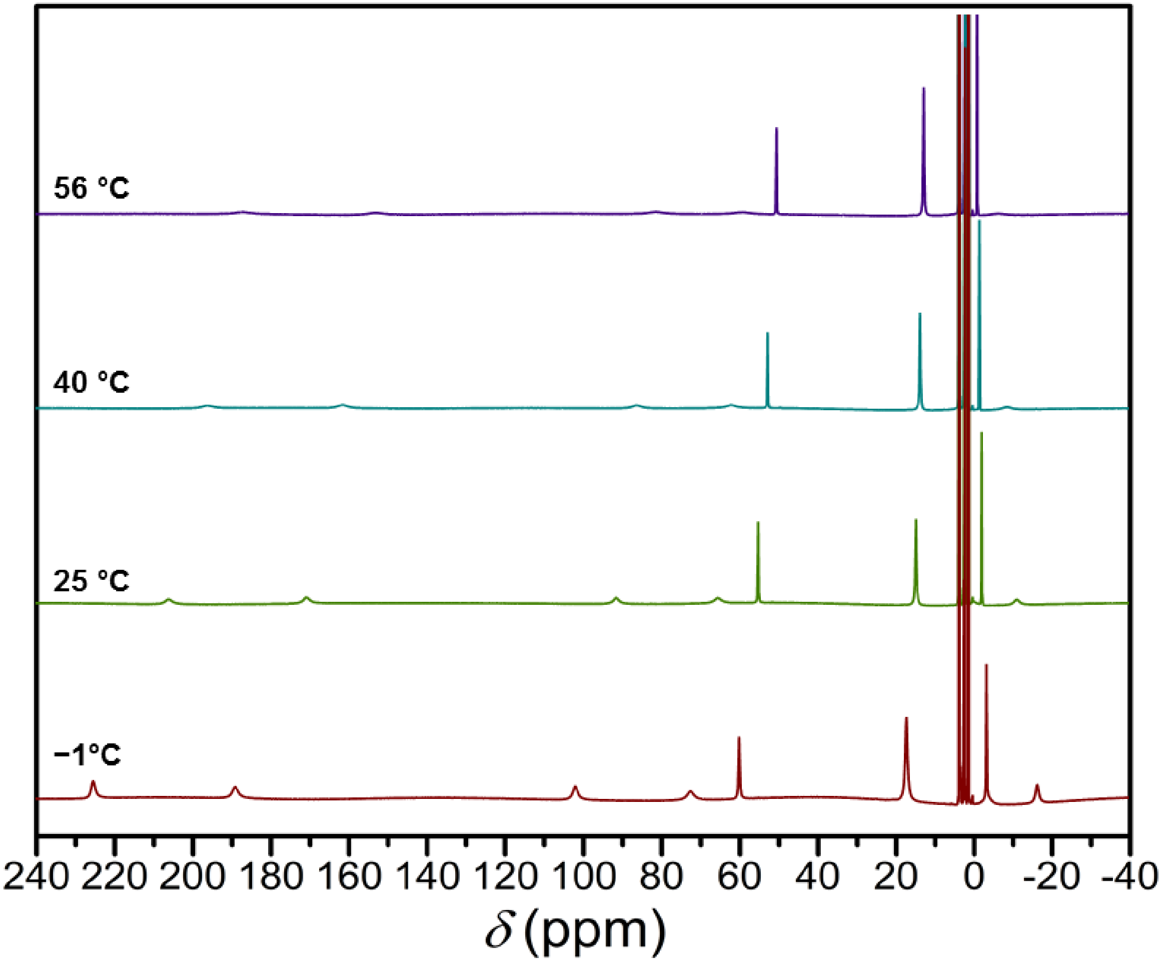

Figure S24 | Variable-temperature ^{1}H NMR spectra of **2a** in CD_3CN at −1 to 56 °C.

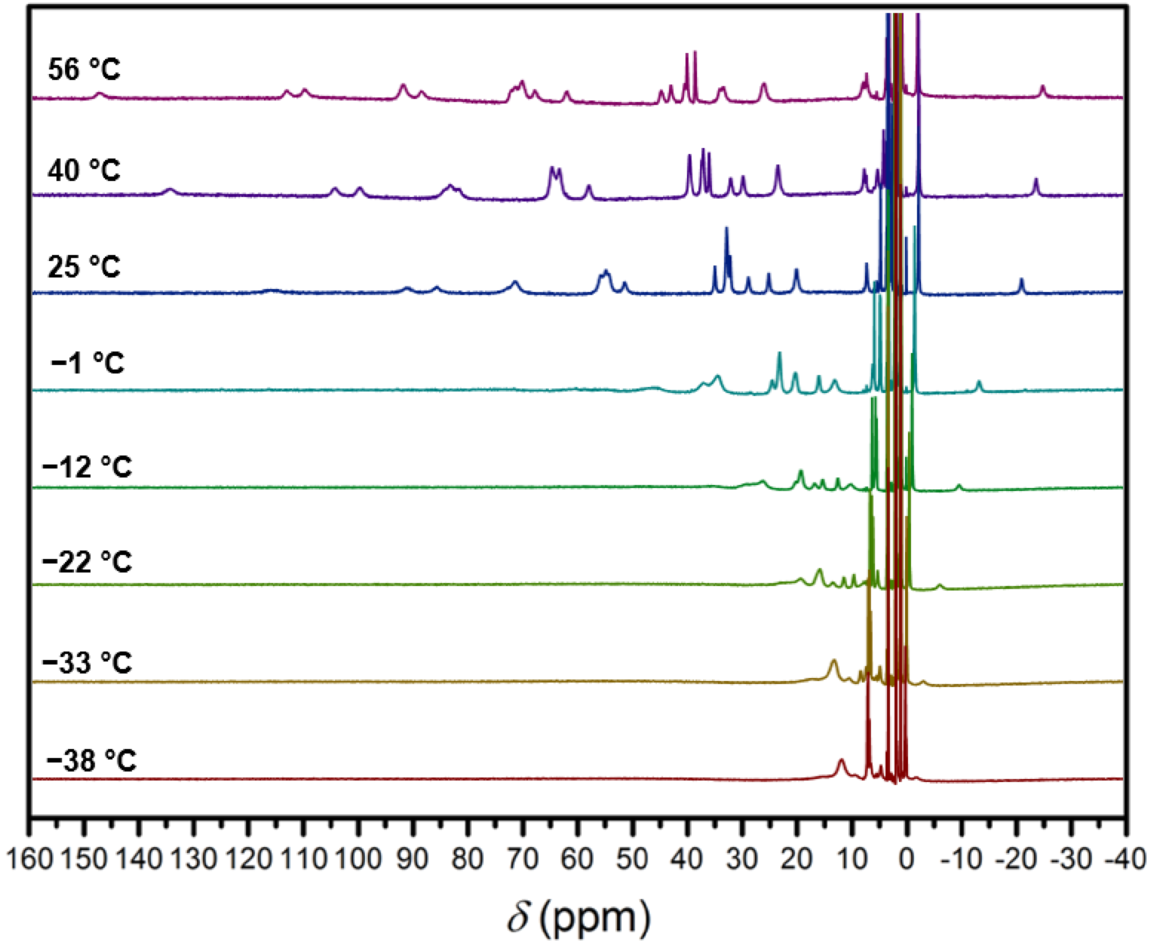

Figure S25 | Variable-temperature ^{1}H NMR spectra of **1a** in CD_3CN at −38 to 56 °C.

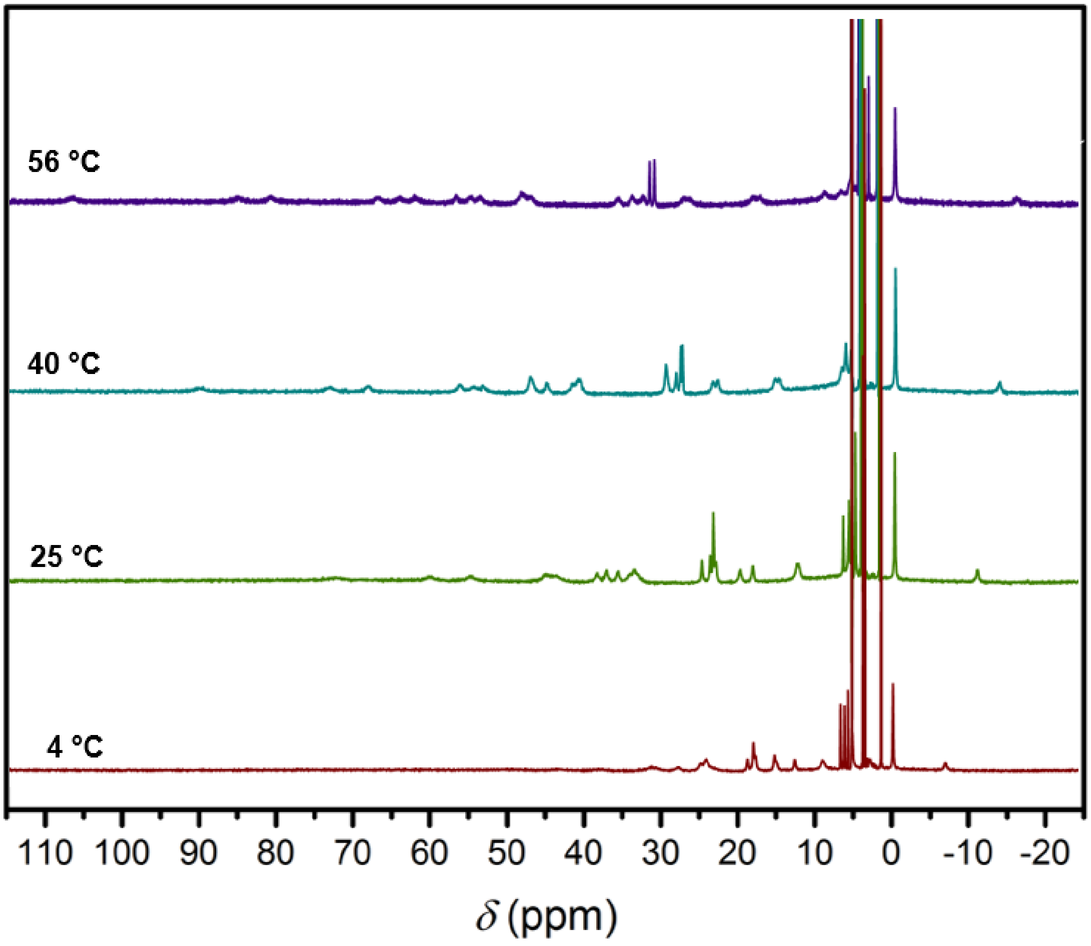

Figure S26 | Variable-temperature ^{1}H NMR spectra of **1a** in D_2O at 4 to 56 °C.

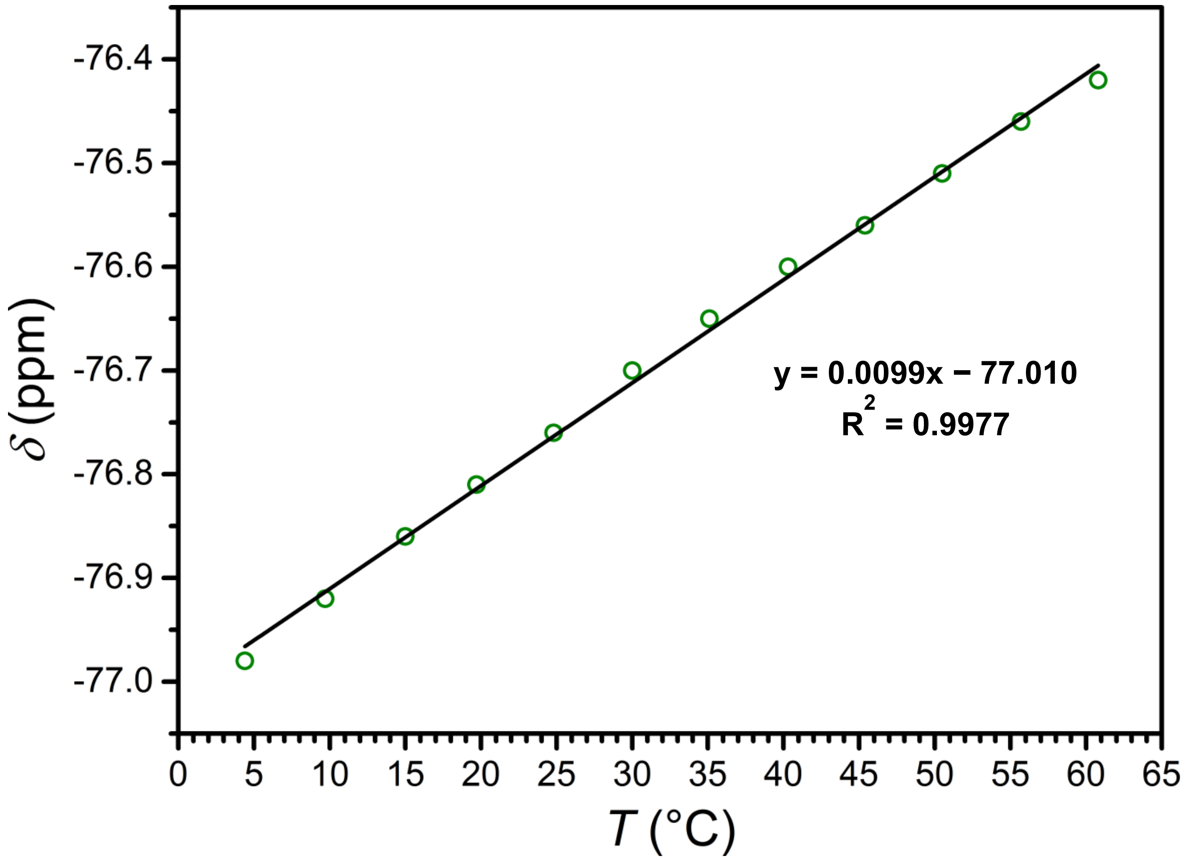

Figure S27 | Plot of the temperature dependence of the ^{19}F NMR chemical shift of trifluoroethanol (TFE) in 2.1 mM H_2O solution. The chemical shift values are referenced to $CFCl_3$. TFE was used as an internal standard for ^{19}F NMR measurements of compounds **1a**, **1b**, **2a**, **2b**, and **3a** in H_2O, where the chemical shift of TFE at each measured temperature was set to the values shown in the graph above and Table S2 below. The black solid line corresponds to a linear fit to the data.

Table S2 | Chemical shifts of ^{19}F NMR resonances of the internal standards used in this study, TFE and NaF, in H_2O.[a]

***T* (°C)**	**^{19}F NMR chemical shift (ppm)**	
	**TFE**	**NaF**
4	−76.98	−118.85
10	−76.92	−118.98
15	−76.86	−119.11
20	−76.81	−119.25
25	−76.76	−119.41
30	−76.70	−119.55
35	−76.65	−119.71
40	−76.60	−119.87
45	−76.56	−120.03
51	−76.51	−120.19
56	−76.46	−120.35
61	−76.42	−120.52

[a] Chemical shift values are referenced to $CFCl_3$.

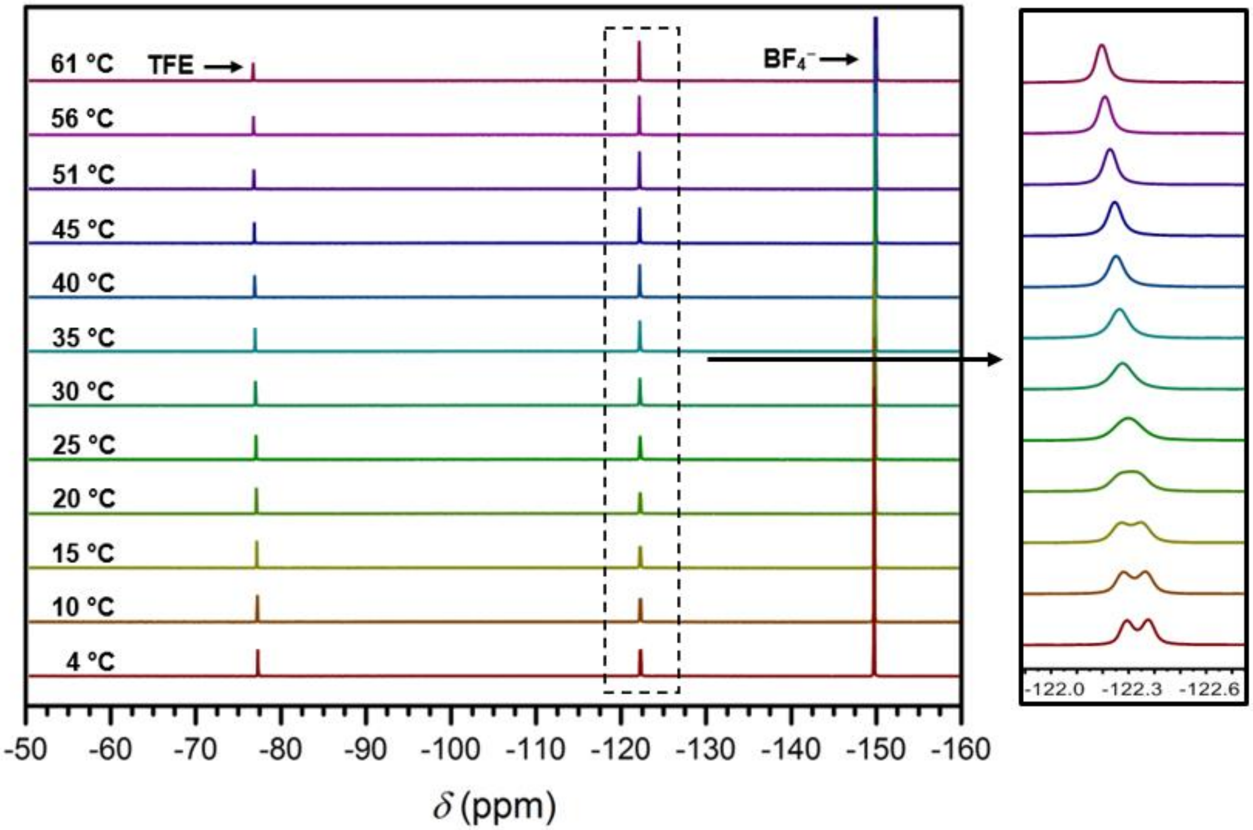

Figure S28 | Left: Variable-temperature ^{19}F NMR spectra of **1b** (7.0 mM) in a 2.1 mM TFE water solution at 4 to 61 °C. Right: Expansion showing the ^{19}F NMR resonances of **1b**, demonstrating the two overlapping resonances observed at 4 °C and the coalescence of the two peaks upon warming.

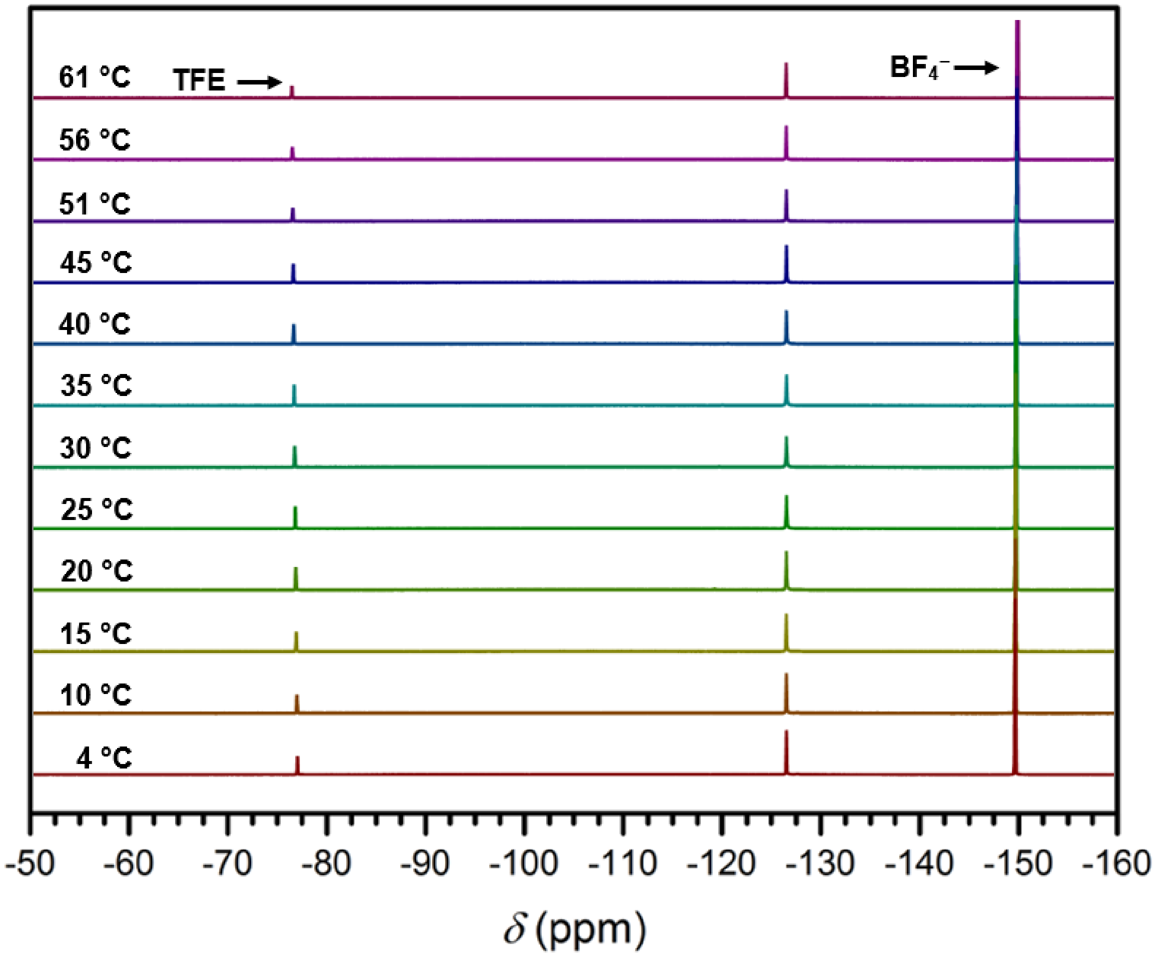

Figure S29 | Variable-temperature ^{19}F NMR spectra of **2b** (10.9 mM) in a 2.1 mM TFE water solution at 4 to 61 °C.

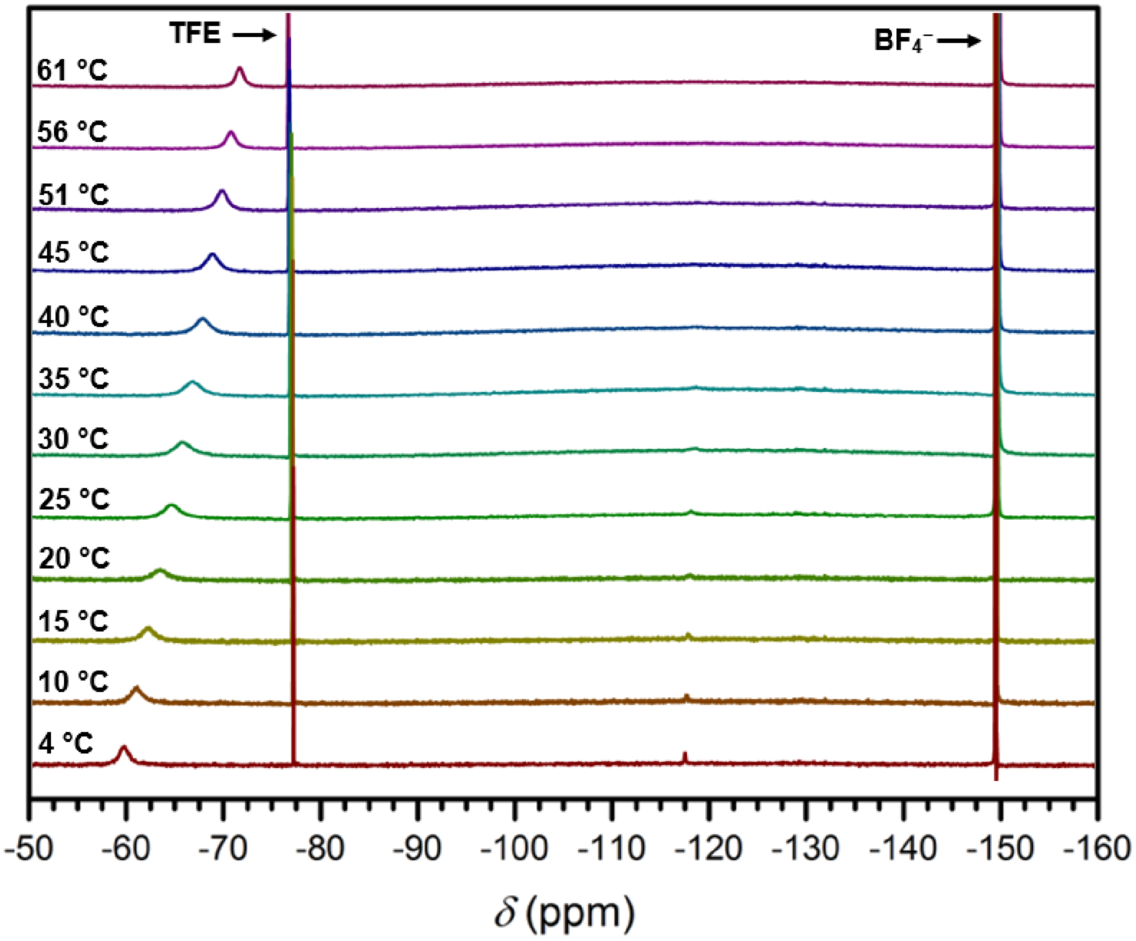

Figure S30 | Variable-temperature ^{19}F NMR spectra of **2a** (12.9 mM) in a 2.1 mM TFE water solution at 4 to 61 °C.

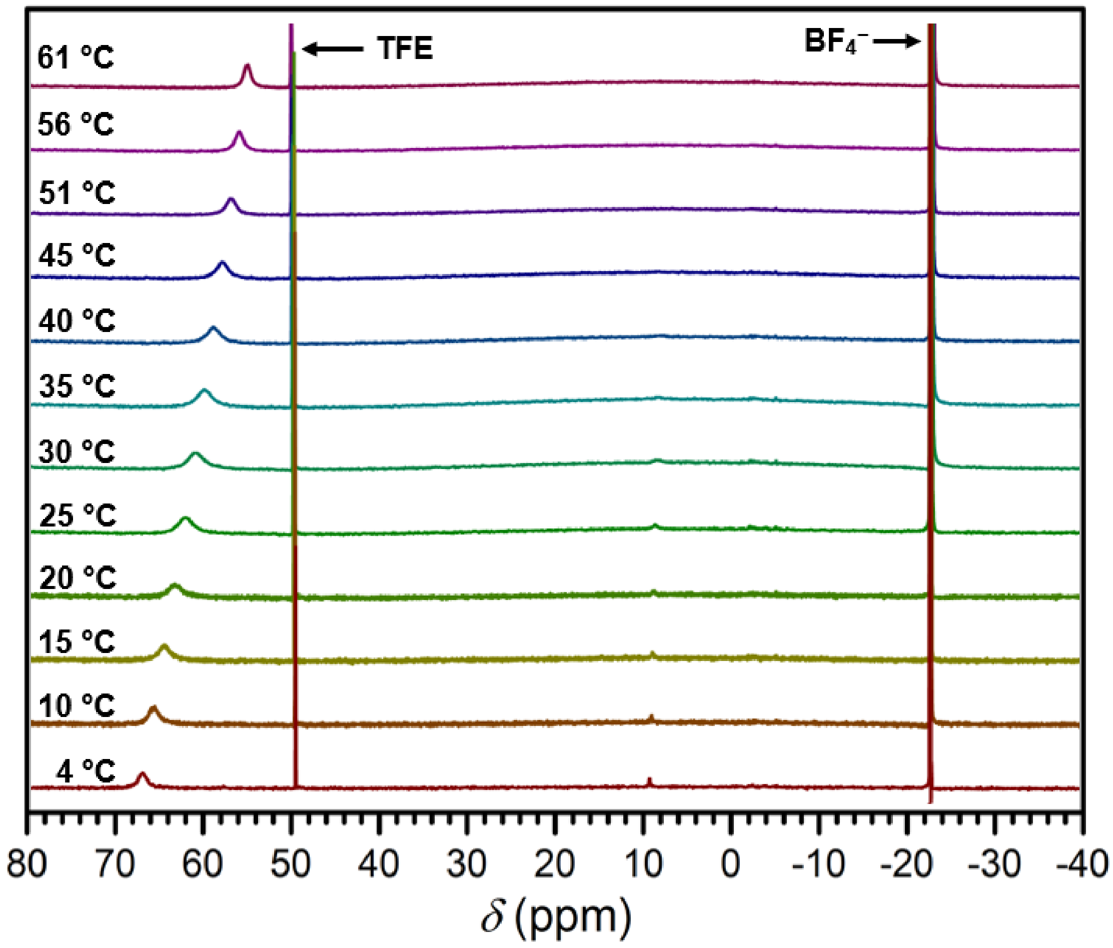

Figure S31 | Variable-temperature ^{19}F NMR spectra of **2a** (12.9 mM) in a 2.1 mM TFE water solution at 4 to 61 °C referenced to its diamagnetic Zn^{II} analogue **2b** at 0 ppm.

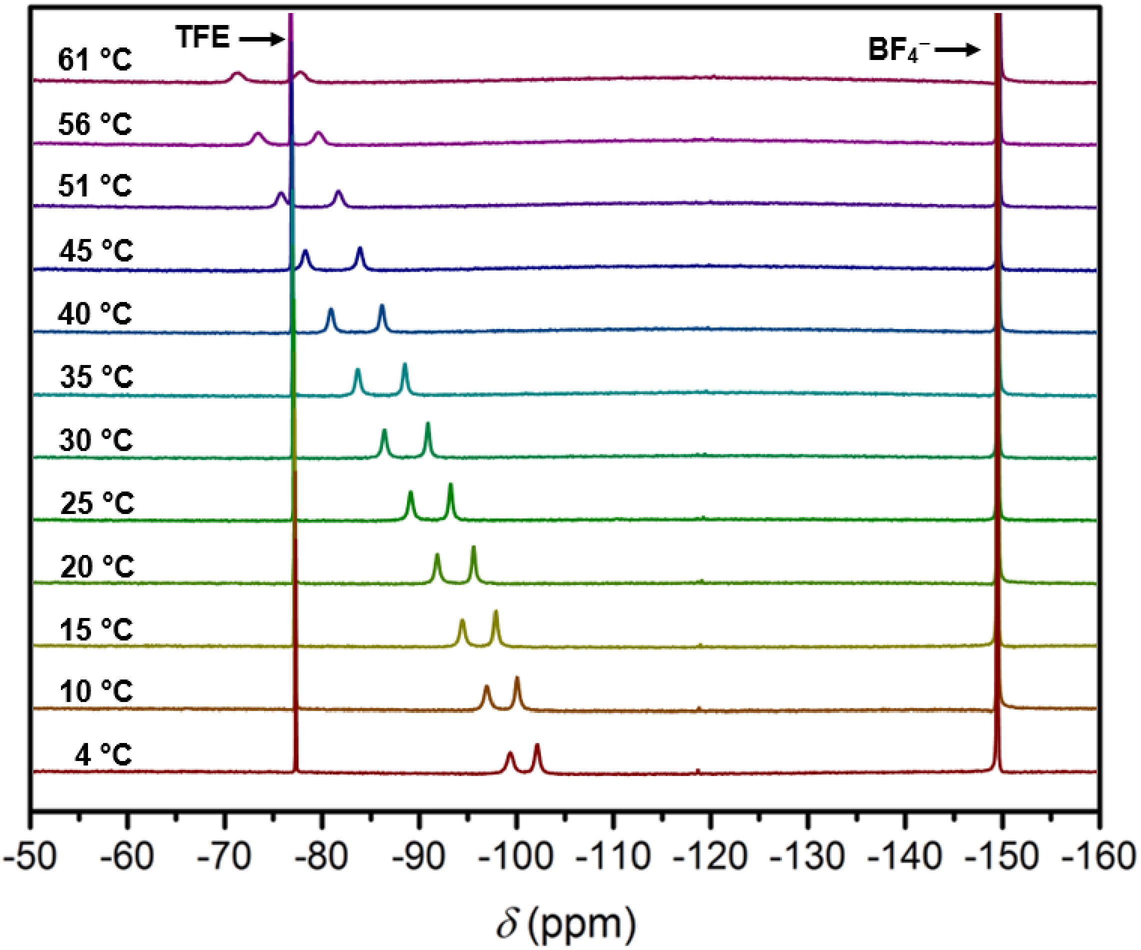

Figure S32 | Variable-temperature ^{19}F NMR spectra of **1a** (14.4 mM) in a 2.1 mM TFE water solution at 4 to 61 °C.

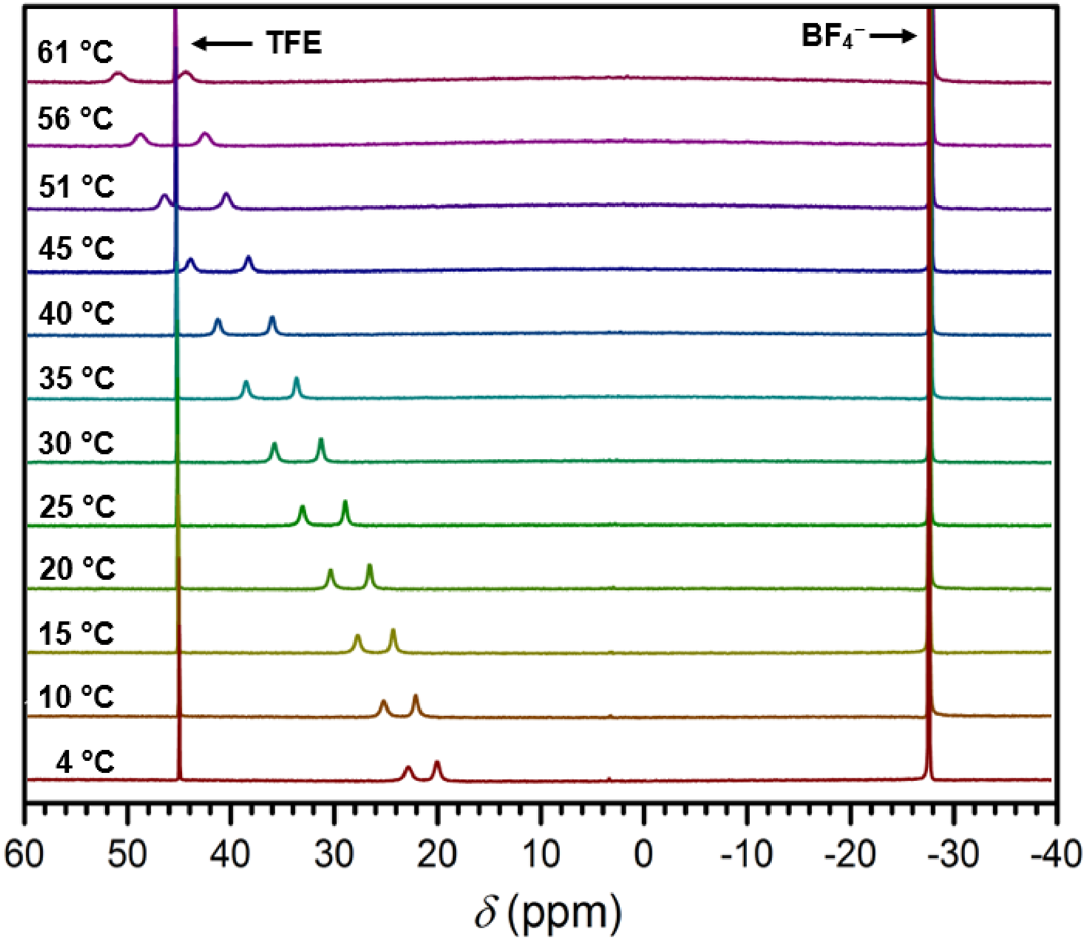

Figure S33 | Variable-temperature ^{19}F NMR spectra of **1a** (14.4 mM) in a 2.1 mM TFE water solution at 4 to 61 °C referenced to its diamagnetic Zn^{II} analogue **1b** at 0 ppm.

Table S3 | Chemical shifts and peak widths of ^{19}F NMR resonances of compounds **1a**, **1b**, **2a** and **2b**, in 2.1 mM TFE water solutions as a function of temperature.

T **(°C)**	**1a**		**2a**	**1b**	**2b**
	**^{19}F NMR chemical shift (ppm)**				
4	−99.25	−102.06	−59.44	−122.33[a]	−126.77
10	−96.86	−99.97	−60.71	−122.32[a]	−126.76
15	−94.31	−97.76	−61.94	−122.32[a]	−126.76
20	−91.68	−95.48	−63.08	−122.32	−126.76
25	−88.91	−93.08	−64.39	−122.30	−126.77
30	−86.17	−90.70	−65.37	−122.28	−126.76
35	−83.39	−88.30	−66.49	−122.26	−126.77
40	−80.65	−85.92	−67.57	−122.25	−126.77
45	−77.97	−83.62	−68.61	−122.25	−126.77
51	−75.45	−81.43	−69.56	−122.23	−126.76
56	−73.02	−79.36	−70.47	−122.21	−126.75
61	−70.92	−77.44	−71.40	−122.20	−126.74
	**Δδ (ppm)**				
	28.33	24.62	−11.96	0.13	0.03
	**Temperature coefficient (CT) (ppm °C^{-1})**[b]				
	0.52(1)	0.45(1)	−0.21(1)	0.002(1)	0.0002(1)
T **(°C)**	**Peak width in FWHM (Hz)**				
4	371	266	561	56	25
10	311	235	635	63	28
15	272	210	745	74	32
20	235	189	854	71	36
25	244	192	955	56	41
30	243	196	990	46	42
35	264	214	960	39	42
40	282	243	868	33	38
45	332	298	761	30	30
51	444	365	614	28	27
56	553	501	510	25	28
61	655	634	421	25	26
T **(°C)**	**|CT|/FWHM (°C^{-1})**[c]				
40	0.87	0.87	0.11	0.03	0.002

[a] Value based on the center of the two overlapping peaks.
[b] CT values are given by the slopes of the linear fits to the data of δ vs T plots.
[c] Calculated for FWHM (ppm) measured at 40 °C.

Table S4 | Chemical shifts of ^{19}F NMR resonances of Fe^{II} compounds **1a** and **2a** in 2.1 mM TFE water solutions as a function of temperature, referenced to their corresponding Zn^{II} analogues **1b** and **2b**.

***T* (°C)**	**1a**		**2a**
	**^{19}F NMR hyperfine shift (ppm)**		
4	23.08	20.27	67.33
10	25.46	22.35	66.05
15	28.01	24.56	64.82
20	30.64	26.84	63.68
25	33.39	29.22	62.38
30	36.11	31.58	61.39
35	38.87	33.96	60.28
40	41.60	36.33	59.20
45	44.28	38.63	58.16
51	46.78	40.80	57.20
56	49.19	42.85	56.28
61	51.28	44.76	55.34

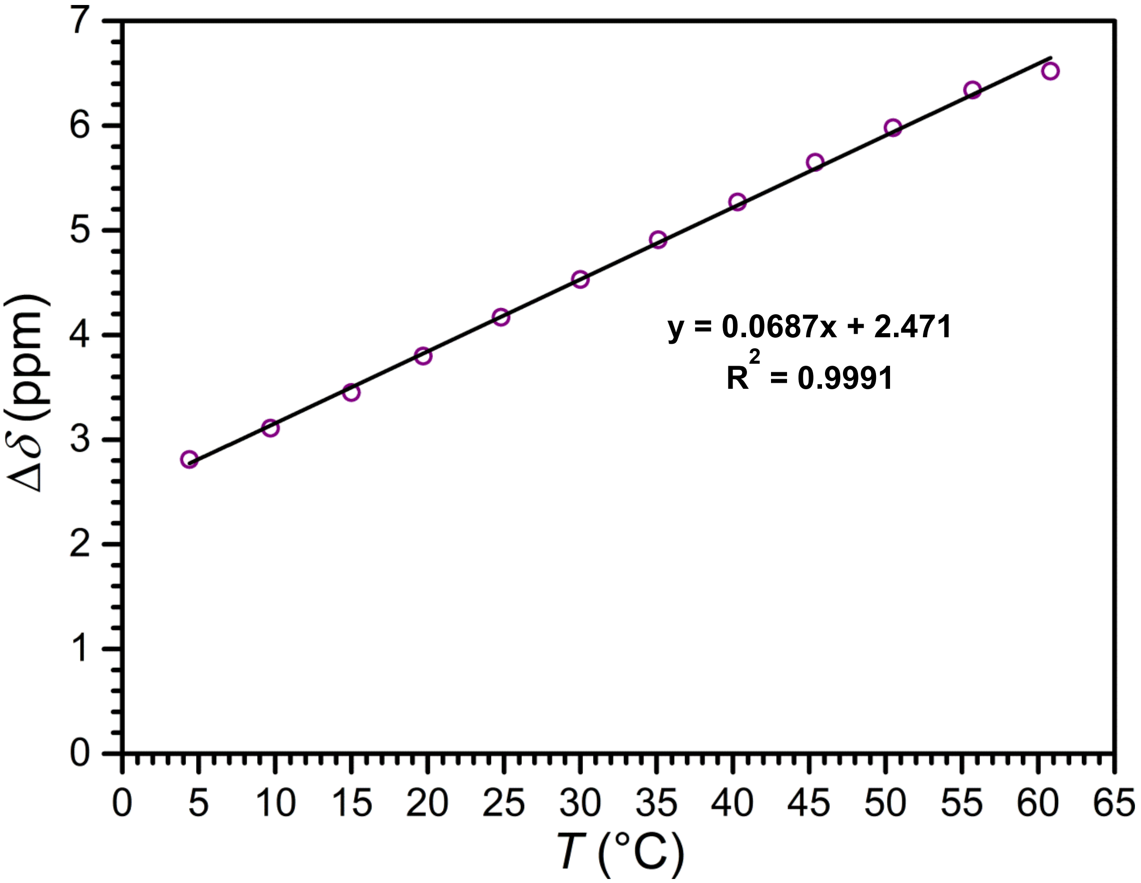

Figure S34 | Plot of the ^{19}F NMR chemical shift separation between the two ^{19}F peaks of **1a** in a 2.1 mM TFE water solution as a function of temperature. The black line corresponds to a linear fit to the data.

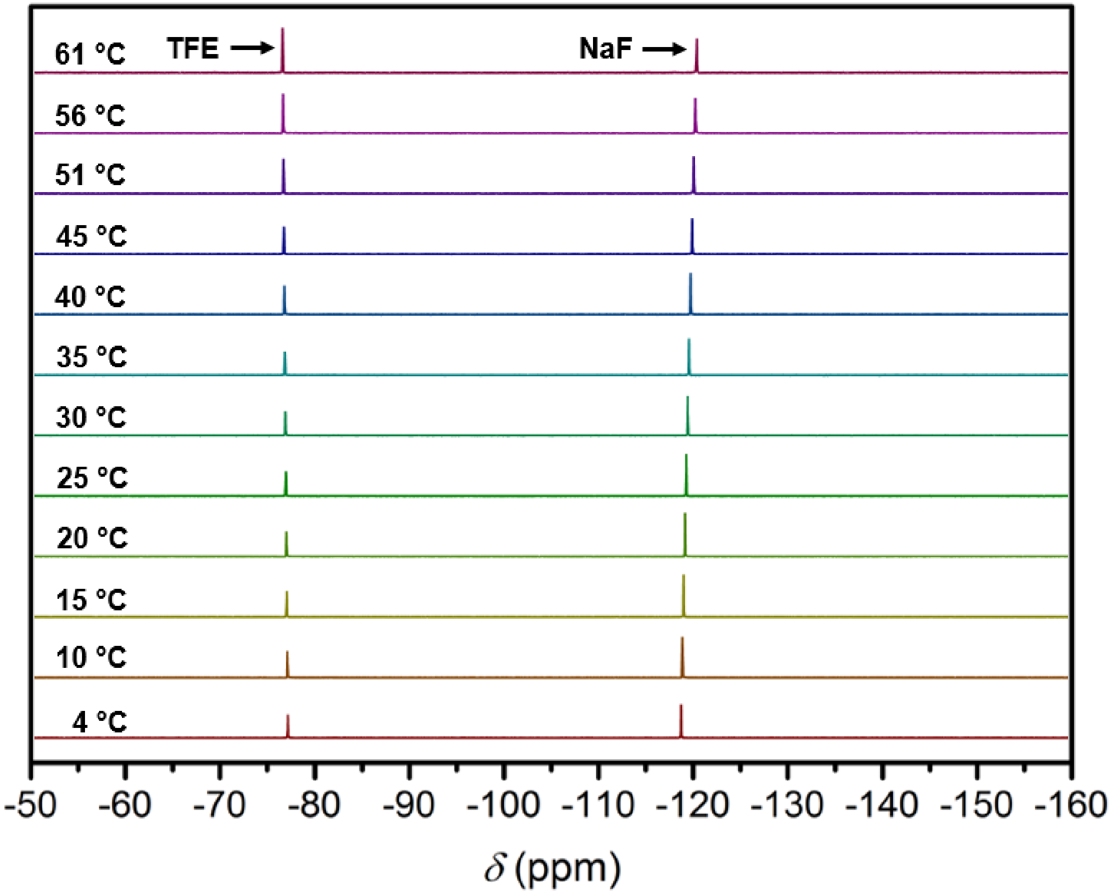

Figure S35 | Variable-temperature ^{19}F NMR spectra of 1.1 mM TFE and 5.2 mM NaF in H_2O at 4 to 61 °C. The chemical shift values are referenced to $CFCl_3$ after being adjusted to the chemical shift of TFE at each measured temperature, as illustrated in Figure S27 and Table S2.

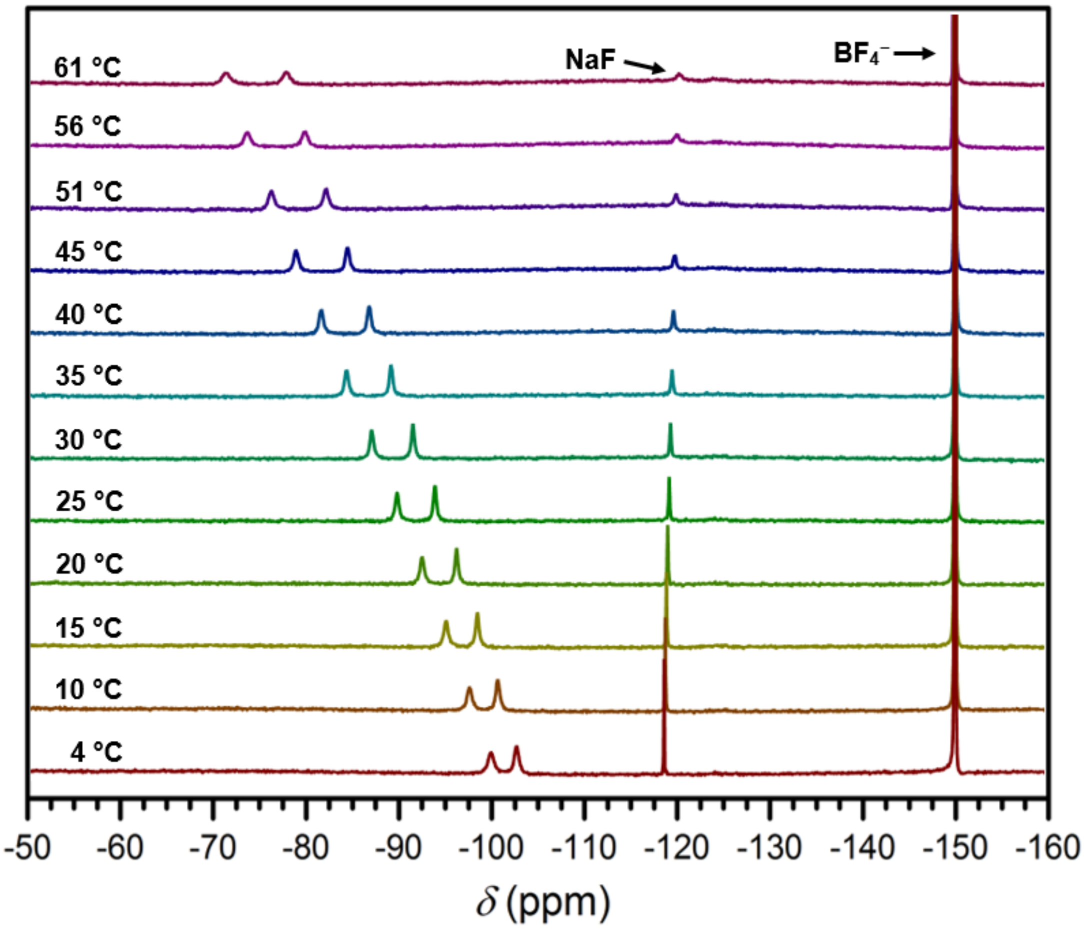

Figure S36 | Variable-temperature ^{19}F NMR spectra of **1a** (13.4 mM) in a 2.1 mM solution of NaF in FBS at 4 to 61 °C. The chemical shift of NaF was adjusted to the chemical shift of TFE at each measured temperature, as illustrated in Figure S27 and Table S2.

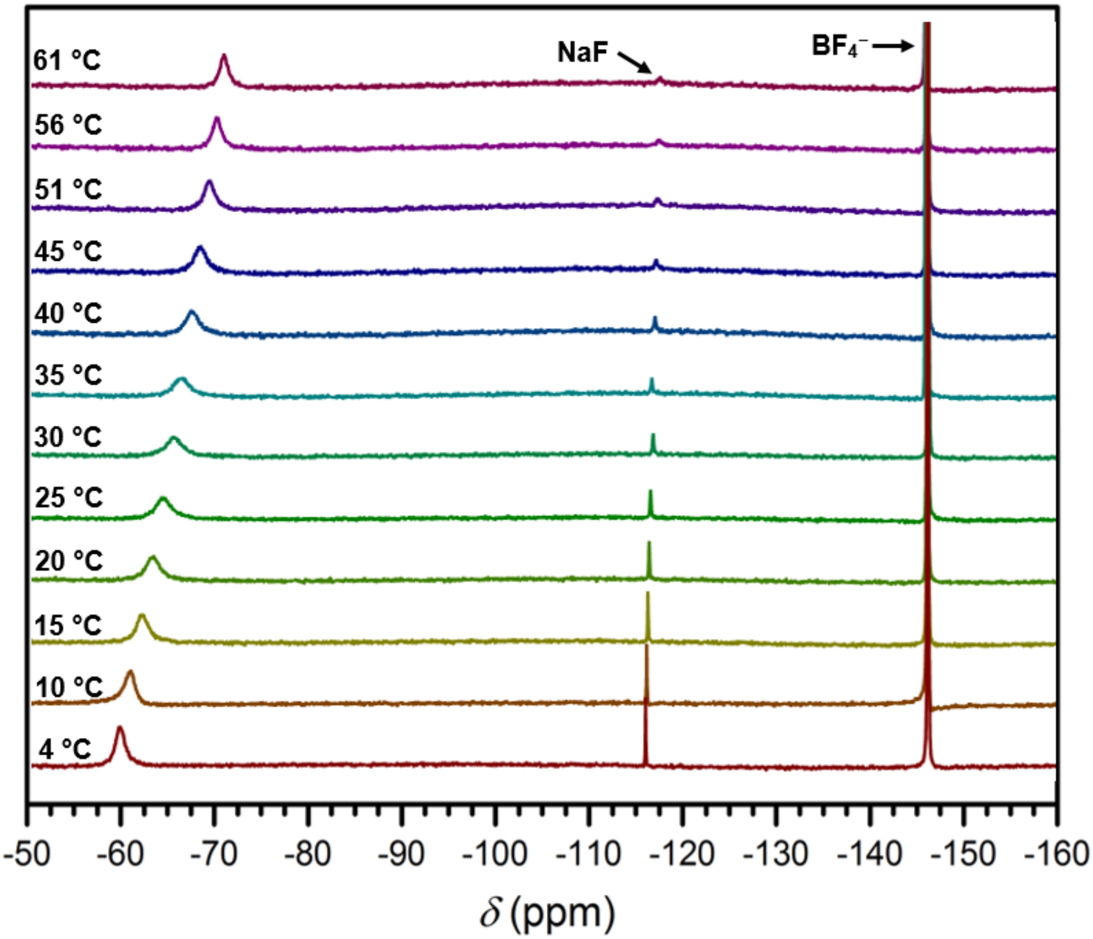

Figure S37 | Variable-temperature ^{19}F NMR spectra of **2a** (15.0 mM) in a 2.1 mM solution of NaF in FBS at 4 to 61 °C. The chemical shift of NaF was adjusted to the chemical shift of TFE at each measured temperature, as illustrated in Figure S27 and Table S2.

Table S5 | Chemical shifts and peak widths of ^{19}F NMR resonances of Fe^{II} compounds **1a** and **2a**, in 2.1 mM solutions of NaF in FBS as a function of temperature.

***T* (°C)**	**1a**		**2a**
	**^{19}F NMR chemical shift (ppm)**		
4	−100.03	−102.82	−59.72
10	−97.70	−100.77	−60.82
15	−95.16	−98.57	−62.20
20	−92.55	−96.30	−63.43
25	−89.84	−93.94	−64.43
30	−87.08	−91.56	−65.72
35	−84.35	−89.18	−66.63
40	−81.59	−86.80	−67.79
45	−78.85	−84.45	−68.74
51	−76.17	−82.10	−69.79
56	−73.56	−79.81	−70.56
61	−71.23	−77.74	−71.40
	**Δδ (ppm)**		
	28.80	25.08	−11.68
	**Temperature coefficient (CT) (ppm °C^{-1})**[a]		
	0.52(1)	0.45(1)	−0.21(1)
***T* (°C)**	**Peak width in FWHM (Hz)**		
4	362	281	582
10	314	241	675
15	275	200	767
20	261	185	869
25	234	182	972
30	228	188	957
35	244	207	1055
40	251	241	872
45	272	254	760
51	342	311	691
56	394	379	588
61	515	467	510
***T* (°C)**	**|CT|/FWHM (°C^{-1})**[b]		
40	0.97	0.88	0.11

[a] CT values are given by the slopes of the linear fits to the data of δ vs T plots.
[b] Calculated for FWHM (ppm) measured at 40 °C.

Table S6 | Chemical shifts of ^{19}F NMR resonances of Fe^{II} compounds **1a** and **2a** in 2.1 mM solutions of NaF in FBS as a function of temperature, referenced to their corresponding Zn^{II} analogues **1b** and **2b**.

T **(°C)**	**1a**		**2a**
	**^{19}F NMR hyperfine shift (ppm)**		
4	22.30	19.51	67.05
10	24.62	21.55	65.94
15	27.16	23.75	64.56
20	29.77	26.02	63.33
25	32.46	28.36	62.34
30	35.20	30.72	61.04
35	37.91	33.08	60.14
40	40.66	35.45	58.98
45	43.40	37.80	58.03
51	46.06	40.13	56.97
56	48.65	42.40	56.19
61	50.97	44.46	55.34

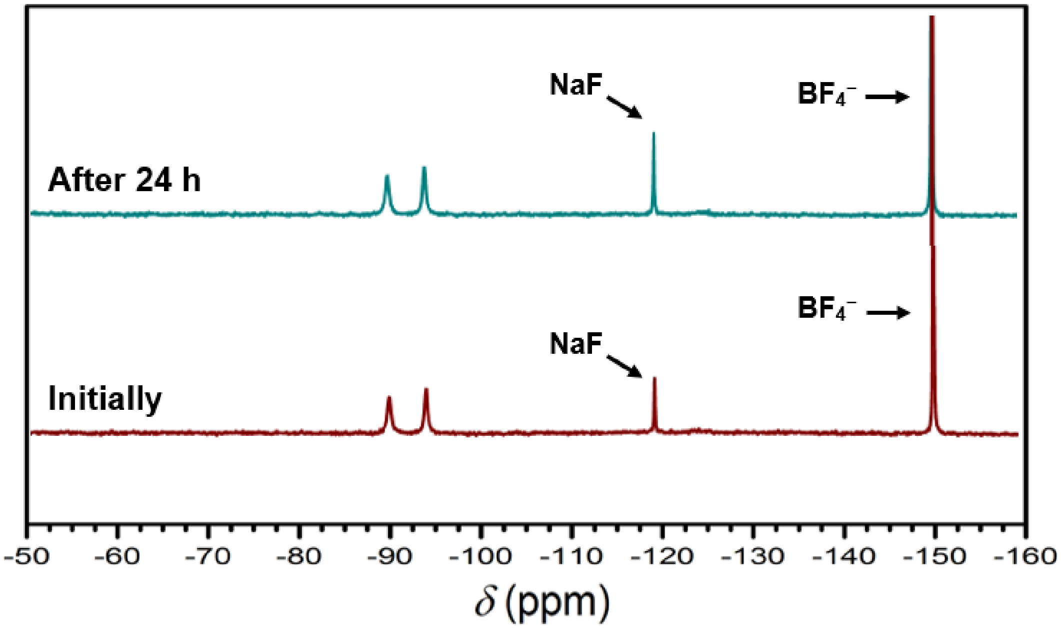

Figure S38 | Comparison of ^{19}F NMR spectra of **1a** (13.4 mM) in a 2.1 mM solution of NaF in FBS recorded at 25 °C. The lower spectrum corresponds to a ^{19}F NMR spectrum of a freshly prepared sample, and the upper spectrum corresponds to a ^{19}F NMR spectrum of the same sample after standing open to air for 24 h at ambient temperature.

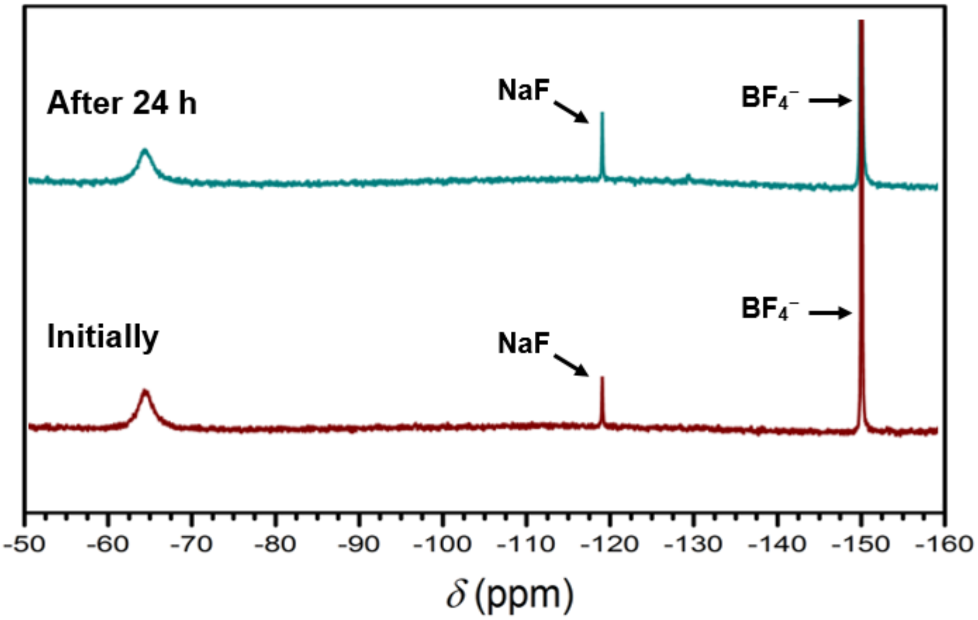

Figure S39 | Comparison of ^{19}F NMR spectra of **2a** (15.0 mM) in a 2.1 mM solution of NaF in FBS recorded at 25 °C. The lower spectrum corresponds to a ^{19}F NMR spectrum of a freshly prepared sample, and the upper spectrum corresponds to a ^{19}F NMR spectrum of the same sample after standing open to air for 24 h at ambient temperature.

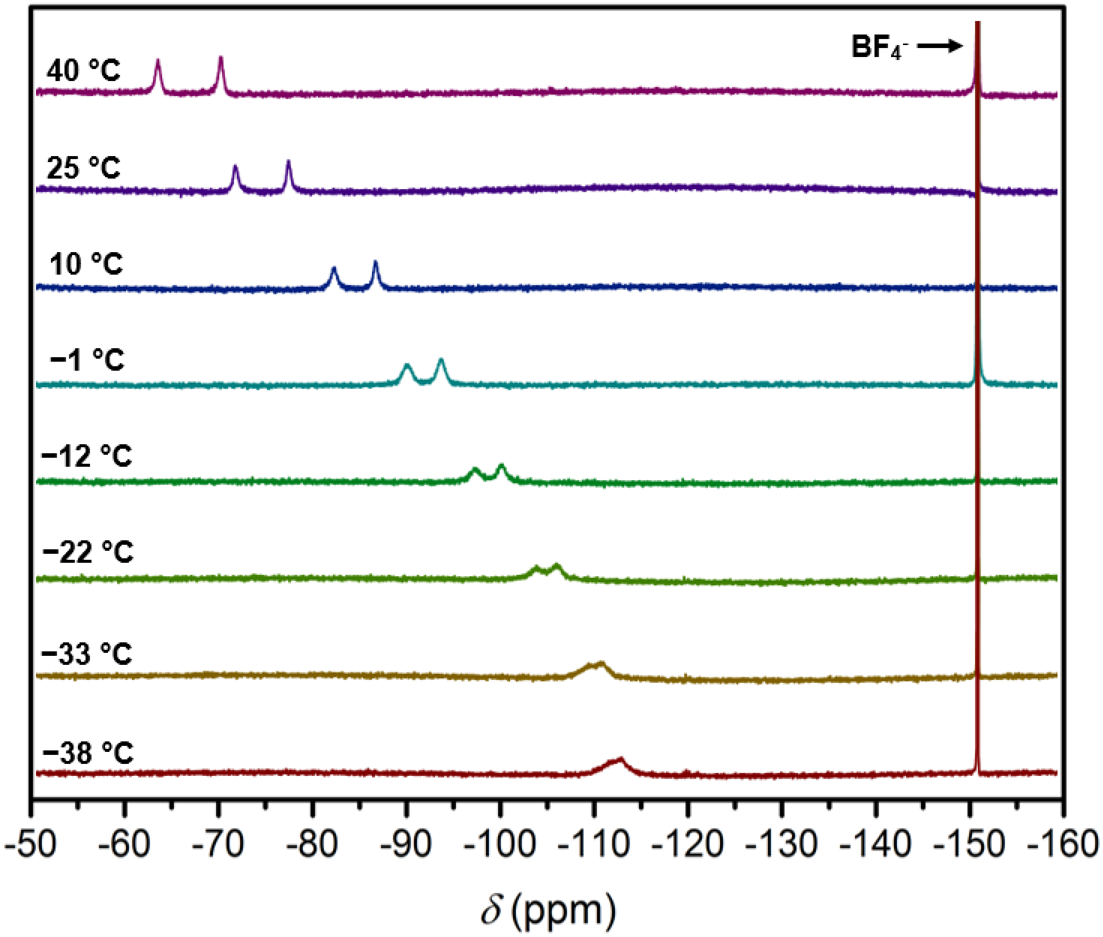

Figure S40 | Variable-temperature ^{19}F NMR spectra of **1a** in CD_3CN at −38 to 40 °C.

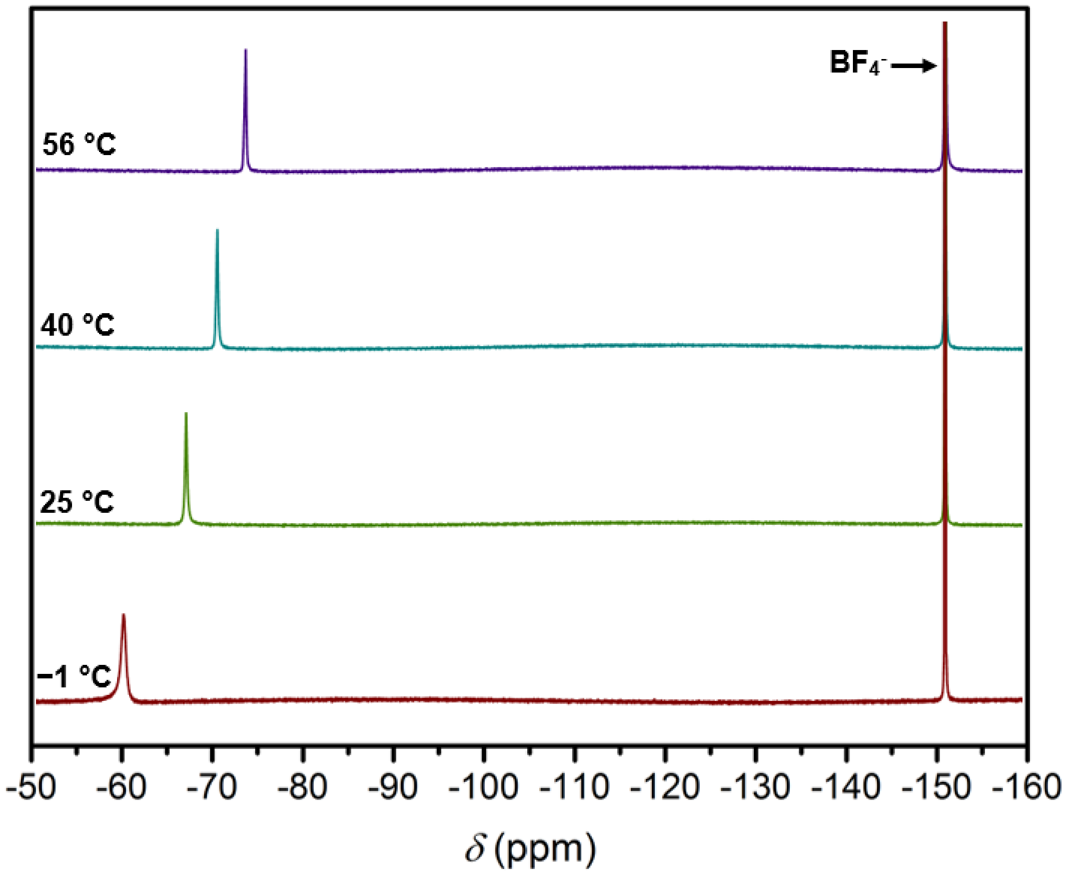

Figure S41 | Variable-temperature ^{19}F NMR spectra of **2a** in CD_3CN at −1 to 56 °C.

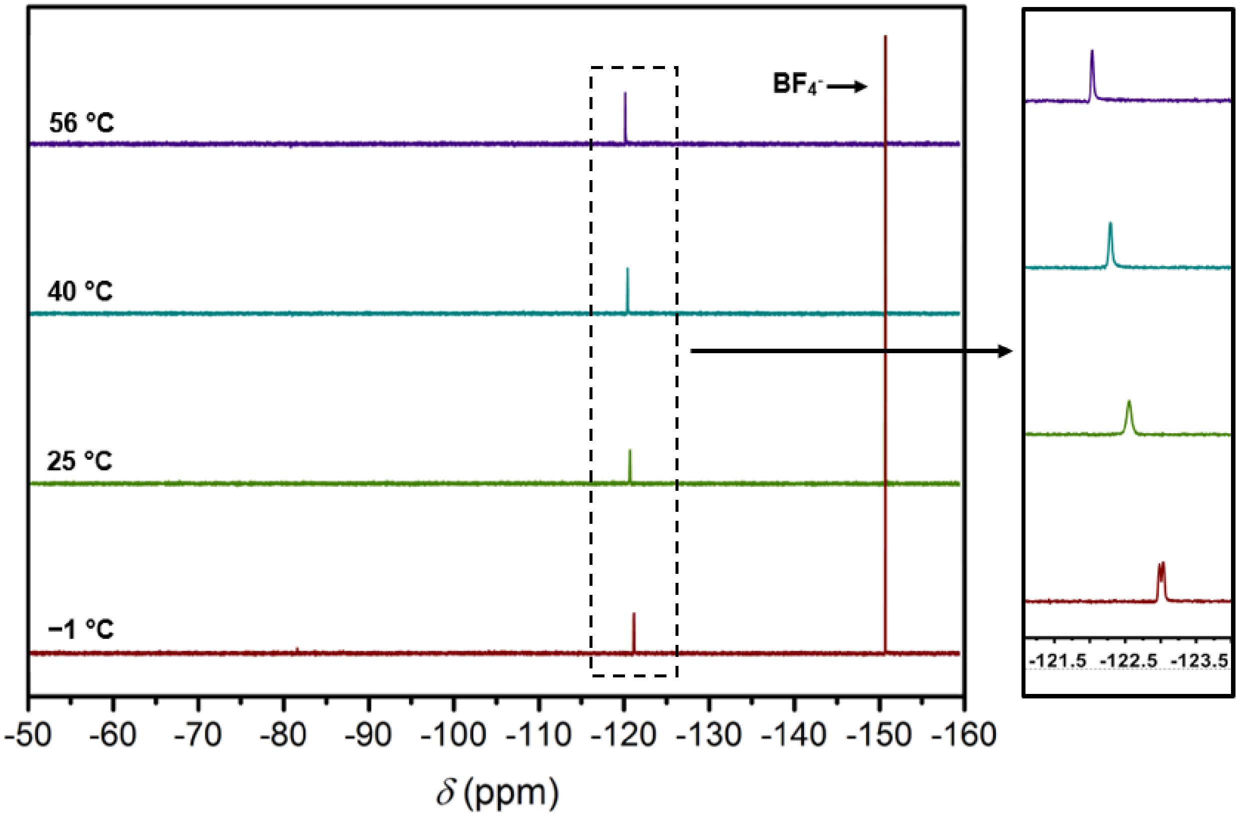

Figure S42 | Left: Variable-temperature ^{19}F NMR spectra of **1b** in CD_3CN at −1 to 56 °C. Right: Expansion showing the ^{19}F NMR resonances of **1b**, demonstrating the two overlapping resonances observed at −1 °C and the coalescence of the two peaks upon warming.

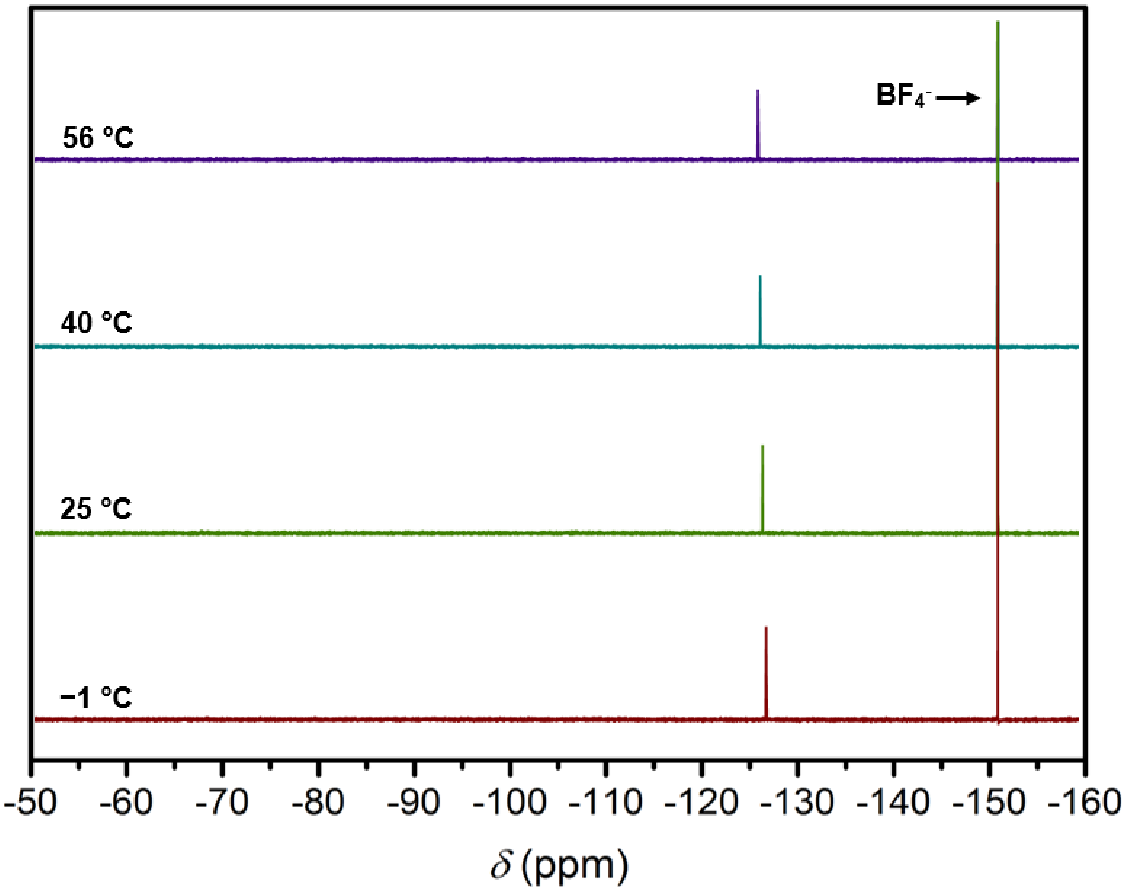

Figure S43 | Variable-temperature ^{19}F NMR spectra of **2b** in CD_3CN at −1 to 56 °C.

Table S7 | Chemical shifts and peak widths of ^{19}F NMR resonances of compounds **1a**, **1b**, **2a** and **2b**, in CD_3CN as a function of temperature.

T **(°C)**	**1a**		**2a**	**1b**	**2b**
	**^{19}F NMR chemical shift (ppm)**				
−38	−112.3[a]		—	—	—
−33	−110.1[a]		—	—	—
−22	−103.80	−105.90	—	—	—
−12	−97.09	−100.03	—	—	—
−1	−89.78	−93.46	−60.06	−123.01[b]	−127.02
10	−81.93	−86.40	—	—	—
25	−71.28	−77.02	−67.02	−122.55	−126.63
40	−62.87	−69.68	−70.48	−122.29	−126.39
56	—	—	−73.63	−122.04	−126.16
	**Δ*δ* (ppm)**				
	40.93[c]	36.22[c]	−13.57[d]	0.97[d]	0.86[d]
	**Temperature coefficient (CT) (ppm °C^{-1})[e]**				
	0.67(2)[c]	0.59(2)[c]	−0.24(2)[d]	0.017(1)[d]	0.015(1)[d]
T **(°C)**	**Peak width in FWHM (Hz)**				
−1	624	504	293	38	10
10	381	296	N/A	N/A	N/A
25	302	246	116	32	17
40	287	270	105	21	20
56	N/A	N/A	150	18	15
T **(°C)**	**|CT|/FWHM (°C^{-1})[f]**				
40	1.10	1.03	1.07	0.38	0.35

[a] Estimated ^{19}F chemical shift because of broad and overlapping resonances, based on the center of the peak.
[b] Value based on the center of the two overlapping peaks.
[c] The temperature range from −22 °C to 40 °C was used for calculations.
[d] The temperature range from −1 °C to 56 °C was used for calculations.
[e] CT values are given by the slopes of the linear fits to the data of *δ* vs *T* plots.
[f] Calculated for FWHM (ppm) measured at 40 °C.

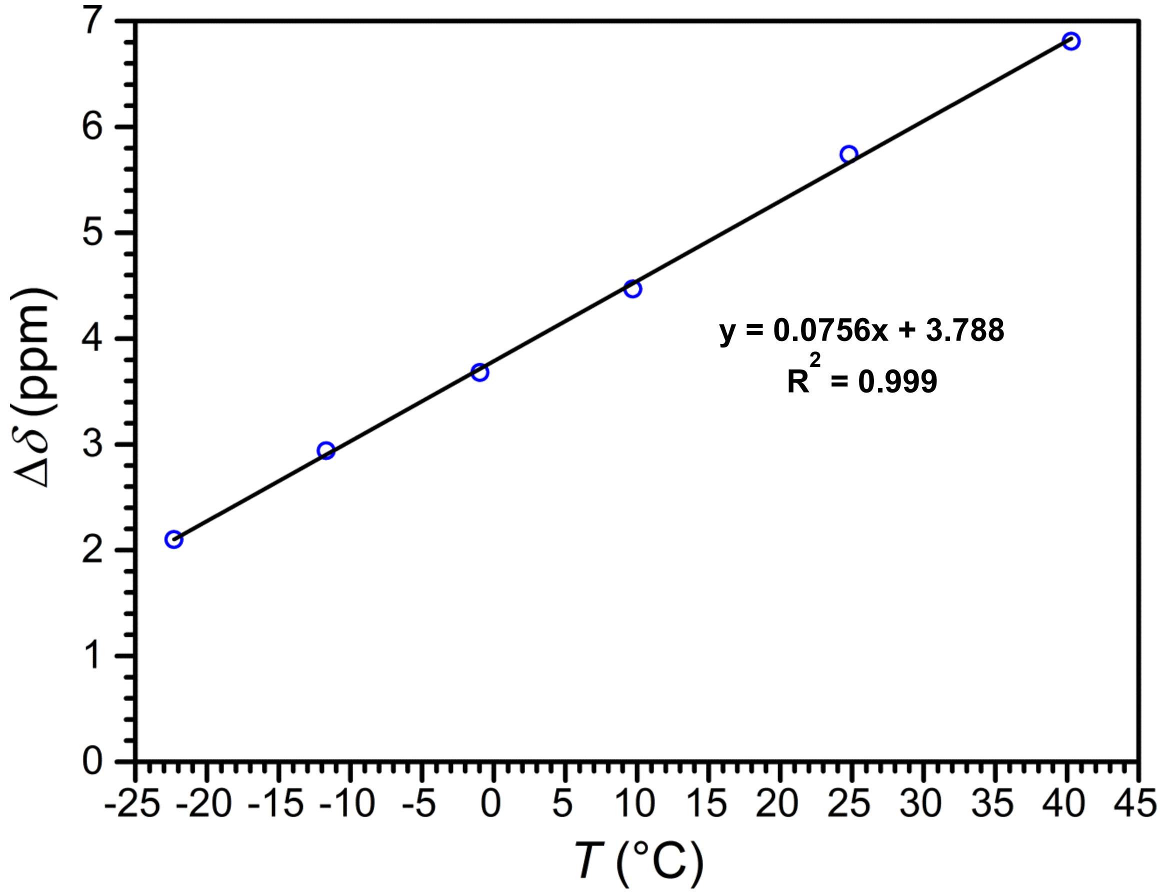

Figure S44 | Plot of the ^{19}F NMR chemical shift separation between the two ^{19}F peaks of **1a** in CD_3CN as a function of temperature. The black solid line corresponds to a linear fit to the data.

References

(1) N. J. Schoenfeldt, A. W. Korinda and J. M. Notestein, *Chem. Commun.*, 2010, **46**, 1640.

(2) D. A. Valyaev, S. Clair, L. Patrone, M. Abel, L. Porte, O. Chuzel and J. L. Parrain, *Chem. Sci.*, 2013, **4**, 2815.

(3) M. Krishnaiah, C. H. Jin, Y. Y. Sheen and D. K. Kim, *Bioorg. Med. Chem. Lett.*, 2015, **25**, 5228.

(4) *SAINT-8.34A Software for the Integration of CCD Detector System*; Bruker Analytical X-Ray Systems, Inc: Madison, WI, USA, 2013.

(5) G. M. Sheldrick, SADABS, version 2.03, Bruker Analytical X-Ray Systems, Madison, WI, USA, 2000.

(6) (*a*) G. M. Sheldrick, SHELXTL, Version 6.12; Bruker Analytical X-ray Systems, Inc.: Madison, WI, 2000; (*b*) G. M. Sheldrich. *Acta Crystallogr., Sect. A: Found. Adv.*, 2015, **71**, 3.

(7) O. V. Dolomanov, L. J. Bourhis, R. J. Gildea, J. A. K. Howard and H. Puschmann, *J. Appl. Crystallogr.*, 2009, **42**, 339.

(8) (*a*) D. F. Evans, *J. Chem. Soc.*, 1959, 2003; (*b*) E. M. Schubert, *J. Chem. Educ.*, 1992, **69**, 61.

(9) G. A. Bain and J. F. Berry, *J. Chem. Educ.*, 2008, **85**, 532.

(10) B. Weber and F. A. Walker, *Inorg. Chem.*, 2007, **46**, 6794.
